# Globular domain structure and function of restriction-like-endonuclease LINEs: similarities to eukaryotic splicing factor Prp8

**DOI:** 10.1186/s13100-017-0097-9

**Published:** 2017-11-07

**Authors:** M. Murshida Mahbub, Saiful M. Chowdhury, Shawn M. Christensen

**Affiliations:** 1 Department of Biology, University of Texas at Arlington, 501 S. Nedderman Drive, Room 337, Arlington, TX 76010 USA; 2 Department of Chemistry and Biochemistry, University of Texas at Arlington, 700 Planetarium Place, Room 130, Arlington, TX 76010 USA

**Keywords:** Transposable element (TE), Line, Non-LTR retrotransposon, Target primed reverse transcription (TPRT), Reverse transcriptase, RNA splicing

## Abstract

**Background:**

R2 elements are a clade of early branching Long Interspersed Elements (LINEs). LINEs are retrotransposable elements whose replication can have profound effects on the genomes in which they reside. No crystal or EM structures exist for the reverse transcriptase (RT) and linker regions of LINEs.

**Results:**

Using limited proteolysis as a probe for globular domain structure, we show that the protein encoded by the *Bombyx mori* R2 element has two major globular domains: (1) a small globular domain consisting of the N-terminal zinc finger and Myb motifs, and (2) a large globular domain consisting of the RT, linker, and type II restriction-like endonuclease (RLE). Further digestion of the large globular domain occurred within the RT. Mapping these RT cleavages onto an updated model of the R2Bm RT indicated that the thumb of the RT was largely protected from proteolytic cleavage. The crystal structure of the large globular domain of Prp8, a eukaryotic splicing factor, was a major template used in building the R2Bm RT model, particularly the thumb region. The large fragment of Prp8 consists not only of a RT similar to R2Bm, but also an RLE and a linker connecting the two regions. The linker sequences adjacent to the RLE in LINEs and Prp8 share a set of two important α-helices and a (presumptive) knuckle/ββα structural motif that are closely associated with the thumb. The RLEs of LINEs and Prp8 share a unique catalytic core residue spacing as well as other key residues.

**Conclusions:**

The protein encoded by RLE LINEs consists of two major globular domains. The larger of the two globular domain contains the RT, linker, and RLE and is similar to the large fragment of the spliceosomal protein Prp8. The similarities are suggestive of possible common ancestry.

**Electronic supplementary material:**

The online version of this article (10.1186/s13100-017-0097-9) contains supplementary material, which is available to authorized users.

## Background

Long INterspersed Elements (LINEs), also called non-LTR retrotransposons, are a major class of retrotransposable elements. LINEs package their transcribed RNA into ribonucleoprotein particles (RNP) using element encoded proteins translated from the mRNA being packaged. LINEs insert their genetic material back into the host genome at a new location by target primed reverse transcription (TPRT) [1–5]. TPRT is initiated by cleavage of one of the target chromosomal strands by an element encoded DNA endonuclease. The free 3′-OH DNA end generated by the DNA endonuclease is used to prime reverse transcription of the element RNA, thus inserting a new DNA copy of element into the host genome.

All LINEs are believed to require the same basic activities to integrate: RNA binding activity, DNA binding activity, DNA endonuclease activity, reverse transcriptase (RT) activity, and completion of integration by second strand synthesis. There are two major groups of LINEs. The two groups share a common RT and a IAP/gag-like CCHC zinc-knuckle. The two groups differ in their open reading frame (ORF) structures, RNA binding domains, DNA binding domains, and DNA endonuclease domains used to form the element RNP and to integrate into the host DNA.

The earlier branching group has a single ORF. The ORF encodes a multifunctional protein with N-terminal zinc finger and Myb motifs, an RT, a gag-knuckle like motif, and a type II restriction-like endonuclease (RLE) with a restriction endonuclease like fold (REL) (reviewed in [6, 7]). This group of LINEs is generally site-specific during integration. The insect R2 element is a well-studied example of this early branching LINE group.

The later branching group has two open reading frames. The second open reading is similar to that of the earlier branching group. It encodes an apurinic-apyrimidinic family endonuclease (APE), a RT, and the gag knuckle-like motif (reviewed in [8–12]). The mammalian L1 element is a well-studied example of this later branching LINE group.

While crystal structures exist for the APE endonuclease and for the protein product of the first ORF of APE LINEs, no crystal or cryo-EM structures exist for the RLE LINEs, nor for the regions common between the two groups of LINEs [13–18]. Our previous paper reported a protein threading model for the restriction-like endonuclease of R2 elements [19]. This paper reports the globular domain structure of R2Bm as probed by limited proteolysis. An updated model of the R2 RT is also presented along with an analysis of the linker region between the RT and the endonuclease. The R2 proteolytic data, in conjunction with sequence-structure alignments of the RT, linker, and RLE, indicate that RLE LINEs share a number of commonalities with the large fragment of Prp8, a highly conserved eukaryotic splicing factor that has a RT domain and an RLE domain, beyond those already discovered and discussed [20–22].

## Results

### Mapping and sequencing LysC protease resistant fragments of R2Bm protein

**Fig. 1:**
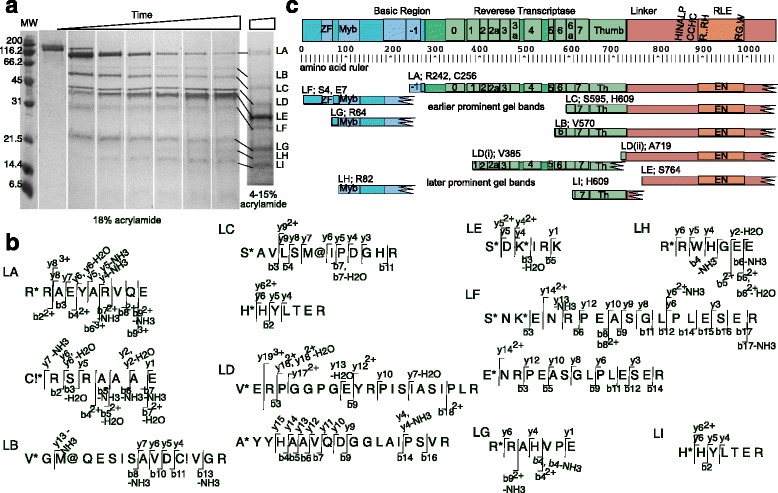
Mapping and sequencing LysC protease resistant fragments of R2Bm protein. **a** R2Bm protein was digested with LysC protease and analyzed by SDS-PAGE. Major observed bands were designated LA-LI. The triangle represents a time course of LysC digestion. The molecular weight (MW) marker values are given. **b** Identification of proteolytic fragments of R2Bm protein. Bands from panel A were cut out, further processed, and analyzed by nano-LC-ESI-MS/MS sequencing. The N-terminal of the band producing fragment was identified by acetylation. Internal peptides were sequenced as well. The y and b ions that identified the N-terminal end are indicated. Symbols: * = acetylation; @ = oxidation;! = carbamidomethylation. The spectrum is given in the supplemental data. **c** Map of the band purified R2Bm fragments. A detailed diagram of the R2Bm open reading frame (ORF) is given along with an amino acid ruler. The boundaries of the R2Bm proteolytic resistant fragments LA-LI are mapped below, along with the amino acid and primary sequence position of the first amino acid of the fragment. The C-terminal ends were not exactly pinpointed but were roughly determined using the apparent MW from the SDS-PAGE gel and by the coverage of internal peptides sequenced by nano-LC-ESI-MS/MS. The major earliest and latest appearing gel bands are roughly grouped together in the map. Abbreviations: zinc finger (ZF) and restriction-like endonuclease (RLE)

In order to probe the globular domain structure of R2Bm, R2Bm protein was subjected to limited proteolysis by one of several proteases. LysC, which cleaves on the C-terminal side of lysine residues, was one of these proteases. There are 42 lysine residues in the expressed and purified R2Bm protein. Aliquots from the digestion reaction were pulled at different time points and the reactions terminated. The digestion profile of R2 protein cleaved by LysC at the different time points were analyzed by SDS-PAGE (Fig. 1a). At least nine major bands (LA-LI) were observed. Some of these bands appeared early in the time course (e.g., LA, LC, LF, and LG), while other bands appeared at later time points (e.g., LE, LH, and LI). Collectively, these bands represent protease resistant R2Bm fragments. The protease resistant fragments were excised from the gel, acetylated, and then digested to completion with trypsin. The peptides resulting from the trypsin digest were sequenced by nano-LC-ESI mass spectrometry. The original N-terminal end(s) of the protease resistant fragment (i.e., those ends resulting from LysC cleavage) were identifiable as they had been acetylated. The N-terminal ends resulting from LysC cleavage in bands LA-LI are reported in Fig. 1b. The y and b ion series that allowed the N-terminal peptide identification are given. The MS/MS spectrum in support of the peptide identification are provided in the Additional file 1: S1A. The internal peptides resulting from further trypsin cleavage of the LysC resistant bands were similarly sequenced by MS/MS (Additional file 1: S1B).

The approximate C-terminal end of LysC protease resistant fragments LA-LI were determined by sequencing of the internal peptides and by the apparent molecular weight of the original protease resistant bands on SDS PAGE gels given the experimentally determined N-terminal end. The peptide sequencing data derived from bands LA-LI have been mapped back onto the linear domain structure of R2Bm and are summarized in Fig. 1c. A more detailed amino acid break down of the different subdomains of the R2Bm ORF can be found in Additional file 2: S6 and in Fig. 5. Please note that the ORF and numbering is for the R2 protein generated from our R2 protein expression construct (ΔNR2Bm) which is slightly amino-terminally truncated compared to the genbank entry for R2Bm.

Full-length R2Bm (118 kD) was quickly processed by LysC to form a large ~89 kD LA band and shorter ~29 kD LF and ~22 kD LG bands. The LF band was found to have a fragment with alternative N-terminal ends that mapped near the beginning of the R2Bm ORF, at amino acid residues four and seven—a serine (S4) and a glutamic acid (E7), respectively. Internal peptides of the LF fragment included the ZF and Myb domains and ended within −1, a conserved basic region involved in RNA binding [23]. The fragment from band LG was similar to the LF fragment, except that fragment LG was ~60 amino acids shorter. Fragment LG had an N-terminal end that mapped to amino acid R64 of the R2Bm ORF, removing most of the ZF from the fragment. The C-terminal end of the LG fragment appeared to be similar to LF.

The polypeptide that constituted the large ~89 kD LA band had two alternative N-terminal ends, R242 and C256. The LA fragment spanned from −1 to the end of the ORF. The LA fragment contained the entire RT, the endonuclease, and the linker region connecting the RT and endonuclease domains.

Another large prominent band appeared along with band LA at the earlier time points: band LC. The fragment from band LC consisted of part of the RT, starting within RT6, at amino acids S595 and H609, and ending at the end of the R2Bm ORF. Like the LA fragment, the LC fragment contained the endonuclease domain in addition to the RT.

 Band LB was present at low amounts across the time series in Fig. 1a. At different protease ratios, however, band LB was only prominent at earlier time points (data not shown). Fragment LB had about 30 more amino acids of the RT than did fragment LC. Fragment LB is likely processed into fragment LC.

At later time points, fragments LA, LB, and LC were further processed. Band LD consisted of two non-overlapping fragments, LD(i) and LD(ii), of about the same size. In the 18% gel the LD fragments ran as a single band, while on the gradient gel a doublet was observed (Fig. 1a). The first LD fragment, LD(i), consisted of the bulk of the RT, from V385, which was located in RT1, through most of the thumb. The second LD fragment, LD(ii) started near the end of the thumb at amino acid A719 and continued through the end of the ORF. Fragment LD(ii) contained the endonuclease and the linker region that connects the endonuclease to the RT.

Fragment LC gets cleaved at K763 to generate bands LE and LI. Band LI consisted of the N-terminal portion of fragment LC with an N-terminal end of H609. The fragment in band LE had an N-terminal end of S764 and contained the linker and RLE. Band LE was a major late appearing band that accumulated over time. Fragments LC and LD(ii) are likely both processed into fragment LE.

Band LH consisted of a fragment with an N-terminal end located at the beginning of the Myb domain at amino acid R82. The polypeptide appeared to be derived from fragments LF and/or LG but was further truncated at the N-terminal end.

As fragments from the RT and the ZF/Myb regions of the ORF were processed into smaller polypeptides, those polypeptides became difficult to resolve and visualize on SDS-PAGE, especially on preparative gels. Depending upon the gel percentage and band location, an excised gel slice can still contain signal from bands just above or below that area. In the later time points, the background between bands increases due to non-banding polypeptides. We did not trust our ability to identify bands and N-terminal ends below about 18 kD.

### Mapping and sequencing of GluC protease resistant fragments of R2Bm protein

**Fig. 2:**
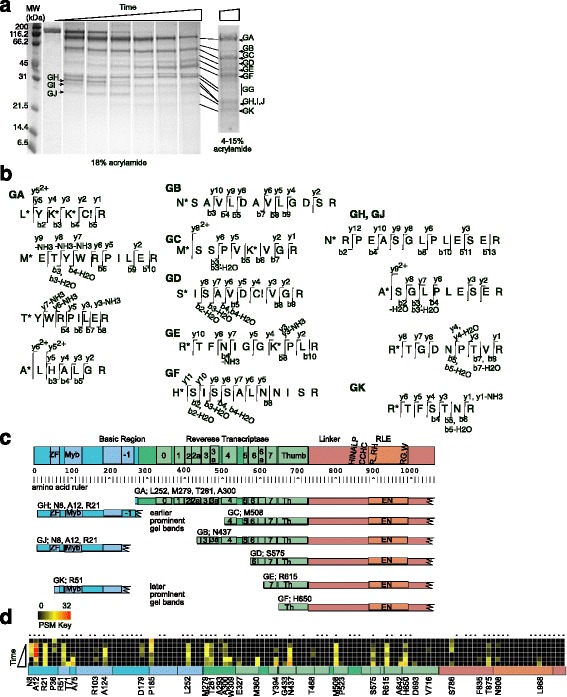
Mapping and sequencing GluC protease resistant fragments of R2Bm protein. Symbols and abbreviations are as in Fig. 1. **a** R2Bm protein was digested with GluC protease and analyzed by SDS-PAGE. Major observed bands were designated GA-GK. **b** Identification of proteolytic fragments of R2Bm protein. Bands from panel A were cut out, further processed, and analyzed by nano-LC-ESI-MS/MS sequencing. The N-terminal of the band producing fragment was identified by acetylation. **c** Map of the band purified R2Bm fragments. The major GluC generated R2Bm fragments detected in panel A are mapped below the ORF diagram and rulers. **d** Heatmap of GluC cleavages found in non-fractionated digestion reactions of R2Bm protein across time. Each column of boxes represents a GluC cleavage site. GluC cleaves after an E residue, indicated by a dot above the column, or a D residue (no dot). The positions of the amino terminal ends generated by observable GluC cleavages are given below the boxes. The number of peptide spectral matches (# PSM) are color coded as shown in the key. The R2Bm ORF is diagramed below the heatmap. Each row is a different time point, with the top row being the zero time point (no GluC) and the bottom row being eight hours. The triangle represents increasing time of GluC digestion

The second protease used to probe globular domain structure of R2Bm was GluC. GluC cleaves on the C-terminal side of glutamic acid residues, and to a lesser extent (100-fold) on aspartic acid residues. There are 69 glutamic acid residues and 47 aspartic acid residues in the R2Bm protein. Aliquots were pulled from the digestion reaction at different time points and terminated. The digestion profile of R2Bm protein cleaved by GluC at the different time points was analyzed by SDS-PAGE (Fig. 2a). The protease resistant bands visualized on the SDS-PAGE were labeled GA-GK. The A-K designators, however, do not necessarily equate to an equivalent LysC resistant R2 fragment, as the designators are by order of apparent-molecular-weight and not by R2 ORF region. The protease resistant fragments were excised from the gel, processed, and sequenced by nano-LC-ESI mass spectrometry. The y and b ion series that allowed the N-terminal peptide identification are given for each band (Fig. 2b and Additional file 3: S2). A map of the internal peptides found in each band are reported in Additional file 4: S3.

GluC, like LysC, quickly cleaved the R2Bm protein into a large fragment of about 87 kD (band GA) and a small fragment of about 30 kD (band GH). The large fragment, GA, consisted of the RT, the linker, and the RLE (Fig. 2c). The small fragment consisted of the N-terminal region of the R2Bm protein. The protein fragments isolated from bands GB-GF were, to a first approximation, further truncations of the GA fragment, where the truncating cleavages were located within the RT. The most prominent of these fragments and bands were GC, GE, and GF. Bands GE and GF appeared late in the time course. The fragments isolated from bands GJ and GK were, to a first approximation, further truncations of band GH. As band GH disappeared, band GJ became more prominent. As band GJ disappeared, band GK appeared. The two bands marked GG were prominent on the 18% acrylamide gel because of a band compression artifact. The GG area contained faint bands and a diffuse smear on the gradient gel. The lower of the two bands appeared to be GluC, while the upper band could not be ascertained. Band GI also could not be ascertained.

There were a number of alternative N-terminal ends found for fragment GA: L252, M279, T281, and A300 (Fig. 2b, c). Fragments GH and GJ also were found to have several alternative N-terminal ends: N8, A12, and R21. In order to aid in interpreting the N-terminal ends of the protease resistant R2Bm fragments (especially early and late cleavage determinations) and to attain a more comprehensive accounting of cleavages that did not give rise to readily observable bands, an experiment was performed where GluC cleavages were detected at a given time point without separating individual proteolytic fragments. Instead of fractionating the fragments, the terminated protease reaction was run into the SDS-PAGE gel for only a few millimeters. A fairly large section of gel near the wells was then excised and processed for cleavage detection (Fig. 2d). This technique of running the reaction minimally into the gel is a near equivalent to direct detection in solution (i.e., no gel fractionation). For technical reasons (see materials and methods), however, it was necessary to have the proteolysis reaction processed through a gel slice. Each column of boxes below the ORF map is a potential GluC cleavage site (D/E), or rather the amino acids immediately following a GluC cleavage site that would become acetylated if the preceding D or E residue were cleaved. Each progressive row is a (longer) time point with identified cleavages reported as a heat map of peptide spectral match (psm) values for each site for each time point. In the heatmap data, there appeared to have been several pre-existing R2Bm N-terminal ends present in the R2Bm protein preparation as N-terminal signals at positions P36, P185, and S786 were detected in the zero time point on the heatmap. No major bands on the SDS-page gels, however, were attributable to these fragments.

Comparing the heatmap results (Fig. 2d) with the data derived from the SDS-PAGE bands (Fig. 2b) provided an extra window into the relative cleavability and timing of several important cleavage sites. It appeared that the major early cleavage events were near the start of the RT. Cleavage at E278 was the most robust cleavage event and gave rise to fragments GA and GH. The cleavage event in domain −1 at position E251 was also a major cleavage event. Cleavage at E251 peaked midway through the digestion reaction as band GJ become prominent. Cleavage at E251 occurred in the full protein as well as in a C-terminal truncation of fragment GH. The T281 and A300 N-terminal ends of band GA appeared to be the result of later cleavage events (at E280 and E299, respectively) that further truncated the original GA fragment.

Another major cleavage event in Fig. 2d was an early event located at E507. Amino acid E507 is within RT4, and cleavage at this location resulted fragment GC. Two other prominent cleavage locations in Fig. 2d, E614 and E649, were later cleavage events and gave rise to fragments GE and GF, respectively. Amino acid E614 is located in RT6, and E649 is located at the beginning of the thumb of the RT. The N-terminal ends of fragments GC and GF were confirmed by Edman degradation (data not shown). Other cleavages were observed within the RT in Fig. 2d, not all of which gave rise to major stable fragments visible on the SDS-PAGE gel. Interior RT fragments (i.e., those not associated with the linker and RLE) were either heterogeneous in nature or unstable such that bands were not observed on an SDS-PAGE gel.

The N-terminal ends of the GH and GJ fragments, like GA, were ragged. The GH and GJ fragments had N-terminal ends of N8, A12, and R21. While all three positions were robust in Fig. 2d, cleavage at E11 to generate the A12 end was the most prominent. It should be noted, however, that the original N-terminal end of the R2 protein was not tracked as the combination of proteases used in generating the peptides for MS/MS sequencing generated peptides too small to be readily detected. There was likely a time dependent shortening of the N-terminal ends in the E7-E50 region of the GH to GJ to GK progression that we were unable to fully quantify. A list of all cleavage sites and early/late data is given in Additional file 5: S7.

### Protein threading model of the R2Bm RT and mapping of the protease cleavages onto the model

**Fig. 3:**
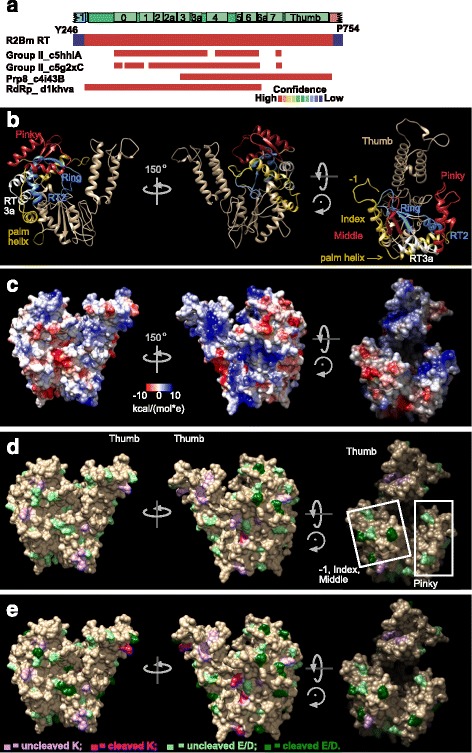
Modeling of the R2Bm RT domain and mapping of the proteolytic cleavages onto the RT model. **a** R2Bm RT model construction and confidence report from Phyre2. 5hhl: crystal structure of the RT domain of the group II intron encoded protein from *Eubacterium rectale*. 5g2xC: the maturase protein in the cryo-EM structures of a spliced *Lactococcus lactis* group II A intron RNP. 4i43B: the splicing factor Prp8 protein large domain crystal structure. 1khv: the crystal structure of rabbit hemorrhagic disease virus RNA-dependent RNA polymerase. **b** Ribbon model representation of R2Bm RT with several key regions highlighted. The pinky finger (RT0) is colored red, as is the middle finger of RT4. The region spanning from a portion of the −1 to RT0 is in yellow. This region includes a remnant of the −1 loop, the index finger α-helix, and the α-helix that traverses palm. The ring finger (RT1) is in blue, as is the RT2 α-helix. **c** Coulombic surface rendering of the R2Bm model. **d** Early proteolytic cleavage sites mapped onto the R2Bm RT model. Dark green coloring marks glutamic acid and aspartic acid residues that were cleaved. Pale green marks glutamic acid and aspartic acid residues that were not cleaved. Pink coloring marks lysine residues that were cleaved. Pale purple marks lysine residues that were not cleaved: See key in panel E. **e** Early plus later proteolytic cleavage sites mapped onto the R2Bm RT model. Markings are as in panel D

It has been nearly 20 years since a model of the R2 RT has been generated using homology modeling and protein threading [24]. The updated RT model shown in Fig. 3 (and Additional file 6: S4) was constructed using the Phyre 2.0 protein modeling server [25]. The model spanned amino acid residues Y246-P754 of the expressed ΔNR2Bm protein and spanned from the end of −1 through the thumb of the RT [19]. The initial residues, Y246-E263, and the final residues, R736-P754, were modeled ab initio by the modeling program. Residues V264-V735, however, were modeled with high homology confidence using four known protein structures as templates: 5hhl (chain A), 5g2X (chain C), 4i43 (chain B) and 1khv (chain A) (Fig. 3a) [21, 22, 26, 27]. The first two templates are group II intron RTs: the cryo-EM structure of lactococcal group II intron LtrA protein and the crystal structure of the *Eubacterium rectale* group II intron RT. The third template is the RT found in the eukaryotic splicing factor Prp8. The fourth template is the caliciviral RNA dependent RNA polymerase. Only the high confidence regions of the R2Bm RT were kept in the final model; the ab initio regions were deleted from the 3D depictions presented in Figs. 3b-e.

The region between −1 and RT0 (V264-P322) was modeled solely from the RNA dependent RNA polymerase (RdRP) but was of high confidence. The region from RT0 through RT2a (I323-R449) was built using the two group II intron RTs and the RNA dependent RNA polymerase. The RT3-RT6 area (K450-L602) was modeled using the group II intron structures, RNA dependent RNA polymerase, and Prp8. The area between RT6 and RT7 was modeled only from Prp8. RT7 was modeled by the group II intron structures as well as Prp8. The thumb was modeled using only the Prp8 crystal structure as a template.

A ribbon diagram of the R2Bm RT model is presented in Fig. 3b. The R2Bm RT assumed the canonical hand-like configuration, with fingers, palm, and thumb regions, and was overall similar to RdRP [28–31]. A word of caution is warranted, homology models are not crystal or cryo-EM structures. The models are comparatively quite crude and resemble their individual templates. That said, homology modeling tools have improved greatly and model with high confidence can be quite informative when one lacks a high resolution structure. The thumb region (1305-1375) was very long and prominent in R2Bm. The −1, index finger (276-288), and middle finger formed one of two bulbous regions as in RdRP. The pinky finger (RT0) formed the second bulbous region. Just behind the index and middle finger was the ring finger (RT1 β-strands). The RT2 α-helix was positioned behind RT0. The region spanning from −1 to RT0 (yellow in the ribbon diagram) includes the index finger (276-288) and the palm-traversing α-helix (298-314).

The index finger and RT0 are connected by the palm traversing α-helix, a feature shared between RdRP, Prp8, and, apparently, LINE polymerases. Telomerases have the index finger α-helix but lack RT0 (the pinky finger) as well as the palm-traversing α-helix (structural overlays of R2Bm RT with PDB ID 3du5 data not shown) [32]. In group II intron RTs, the index finger and palm traversing helix are not present (PDB ID 5hhl and 5g2x) [22, 26]. Group II intron RTs do, however, have an RT0 and an extension to the RT0 termed NTD, both positioned on the pinky finger side [22, 26].

The index finger region is important for the polymerization functions. A monoclonal antibody directed against the vicinity of the index finger of the hepatitis C virus RdRP was found to inhibit both primer-dependent and de novo RNA synthesis [33].

The pinky finger region is also important for polymerization. The RT0 of R2Bm and group II intron RTs share a set of antiparallel α-helices connected by a loop [22, 26, 34, 35]. In RdRP the RT0 homologue is the “G-loop,” or “motif G.” The G-loop functions in template-RNA binding and translocation [28, 36]. A monoclonal antibody directed against the G-loop was found to be inhibitory to primer-dependent RNA synthesis but not de novo RNA synthesis [33]. The RT0 domain of RLE LINEs contains a PGPD motif in the loop. The PGPD motif, when mutated in R2Bm, abolished template jumping activity of the RT and reduced, to some extent, overall polymerization activity [23]. Template jumping activity is also observed in RdRP, Mauriceville retroplasmid, and group II intron RTs [37–39]. Mutation of the PGPD motif in R2Bm also reduced the binding to the 5′ and 3′ PBM RNAs [23]. The group II intron protein’s RT0 and its extension (the NTD) are involved in binding DIVa of the group II intron RNA [22, 40, 41]. The interaction between RT0 and DIVa is required for positioning the intronic-RNA-template for reverse transcription (TPRT), but it is not strictly essential for splicing [40].

RLE LINEs, telomerase, and group II introns possess RNA binding domains upstream (N-terminal) of the reverse transcriptase. In the case of the group II intron protein, the N-terminal domain is an extension of RT0 and resides on that side of the RT (the pinky finger side). The extended RT0 and IFD bind to DIVa of the intron RNA [22, 40]. In R2, the RNA binding domain −1 is on the opposite side of the fingers from RT0. The remnants of domain −1 is on the index finger side. Mutations in −1 abolished 5′ and 3′ PBM RNA binding [23]. Telomerases also contain an RNA binding region upstream of the RT that is involved in binding RNA [32, 42, 43].

A coulombic surface map is presented in Fig. 3c. The R2Bm RT adopts an overall shape of a curved wedge with the backside of the thumb being the sharp edge. One of the two comparatively flat sides is the thumb-to-RT0 face. This face has a small central acidic patch surrounded by mostly hydrophobic residues in the model. The other fairly flat side is the thumb to index finger side and is predominantly basic. The third side is rounded. It spans from the index finger to RT0 and has a central vertical streak of acidic residues running through a central streak of (mostly) hydrophobic residues. The streaks are centered below the ring finger. The hydrophobic regions, and perhaps the acidic patches/streaks within them, are potential areas of further protein-protein interactions.

The R2Bm RT model was used for mapping the earlier cleavages (Fig. 3d) as well as all of the cleavages (early plus later, Fig. 3e) for both LysC and GluC proteases. LysC cleaves on the C-terminal side of K residues. There are 18 K residues in the R2Bm RT model, six of which are cleaved to some degree. Cleavage in the ab initio regions are included in the cleavage count, although the ab initio sequences have been deleted from the 3D models in the figure. GluC cleaves on the C-terminal of E residues and much less often on the C-terminal side of D residues. There are 30 E residues in the R2Bm RT, 14 of which are cleaved to some degree. There are 26 D residues in the RT, six of which are weakly cleaved. Most of the early cleavages mapped to the −1 ab initio regions (not shown), the index finger, and the tip of the middle finger. There was also a cleavage on the basic face between the thumb and −1. Some of the next cleavages were also on the basic face as well as on the RT0 protrusion and on the knife edge (the backside) of the thumb. Most of the prominent thumb was protected from cleavage. Later cleavages were found on the secondary structures just behind where the first cleavages were (i.e., the regions behind the index finger α-helix) and on the flat hydrophobic thumb-to-RT0 face inside the acidic patch.

### The large fragment of the eukaryotic splicing factor Prp8 and restriction-like endonuclease bearing LINEs share a common set of sequence motifs and structure

RTs share a common set of sequence domains, numbered 1-7, and a thumb region [34, 35, 44–47]. The thumb usually contains a three-helix bundle. In addition to the thumb and RT1 through RT7, the RT of LINEs contains insertions: 0, 2a, 3a, and 6a. Several of these insertions are present in other eukaryotic RTs (Additional file 7: S5A-D and [34, 35, 44–46]). The RT domain of Prp8 is very similar to that of LINEs, having 0, 2a, 3a, 4a, and 6a insertions. The telomerase RT encodes 2a, and 3a. The RT of group II intron proteins encodes 0, 2a, 3a, 4a, and 7a.

The area between the reverse transcriptase and the RLE in RLE LINEs is the linker region. The linker in RLE LINEs was predicted to be predominantly α-helical with six major helices, with some groups having 2-3 additional helices (Additional file 7: S5). A weak scoring helix also was often observed in the highly-conserved (presumptive) gag-knuckle (see below). The region downstream of the RT in APE LINEs were more diverse, with 5 -14 predicted helical regions. The crystal structure and EM-structures of Prp8 have about 13 helices. β-strands were less prevalent in the linker of RLE LINEs than in APE LINEs (about 0-2 vs 4-6). Among the RLE LINEs, only Utopia may contain comparatively high number of linker β-strands. Several clades of APE LINEs encode an RNaseH domain downstream of the RT (reviewed in [9]).

**Fig. 4:**
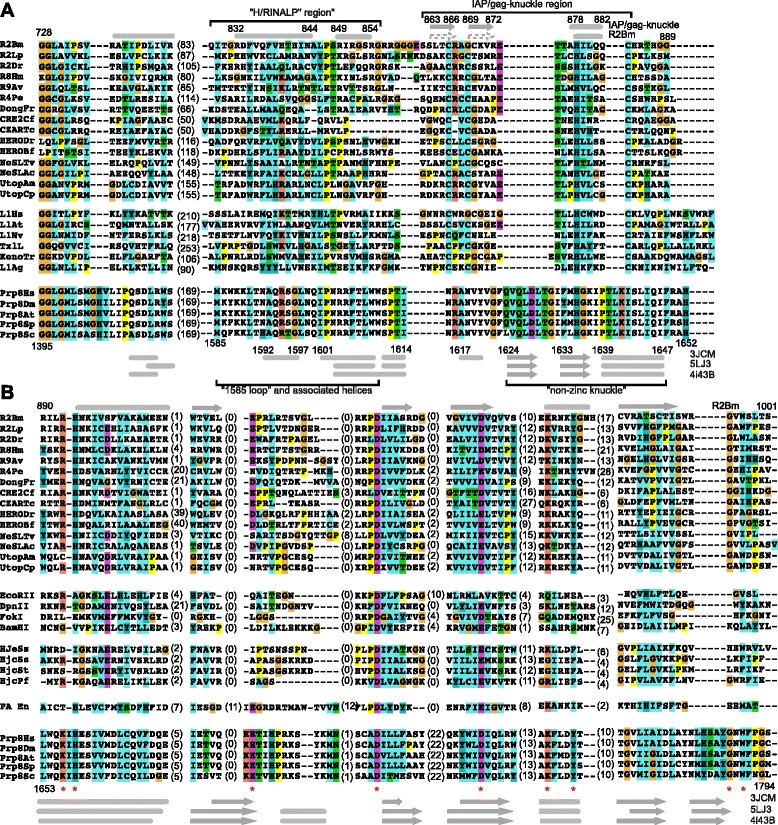
Structure based alignments of linker and RLE regions. **a** Multiple sequence alignment and Ali2D prediction of the most conserved region of the linker. The Ali2D predicted secondary for R2Bm protein are marked above the R2Bm sequence. The canonical ββα structure of a gag-knuckle/IAP is also presented. The secondary structure of Prp8 is given below the Prp8 sequences. The Prp8 secondary structures are from several reported crystal and cryo-EM structures: 3JCM, 5LJ3, 4i43B. Rounded bars are α-helices and arrows are β-strands. **b** Multiple sequence alignment RLE from LINEs, type II restriction enzymes, archeal Holliday junction resolvases, influenza PA endonuclease, and Prp8. Abbreviations: R2Bm = *Bombyx mori* (M16558.1); R2Lp = *Limulus polyphemus* (AF015814.1); R2Dr = *Danio rario* (34392533); R8Hm = *Hydra magnipapillata*; R9Av = *Adenata vaga* (ACV95454.1); R4Pe = *Parascaris equorum* (AAB02297.1); DongFr = *Fugu rubripes*; CRE2Cf = *Crithidia fasciculata*; ZARTc = *Trypanosoma cruzi*; HERODr = *Danio rario*; HEROBf = *Branchiostoma floridae*; NeSLTv = *Trichomonas vaginalis*; NeSLAc = *Acanthamoeba castellanii*; UtopAm = *Alligator mississippiensis* Utopia; UtopCp = *Crocodylus porosus* Utopia; Prp8Hs = *Homo sapiens* (NP_006436.3); Prp8Dm = *Drosophila melangaster* (NP_610735.1); Prp8At = *Arabidopsis thaliana* (Q9T0I6); Prp8Sc = *Saccharomyces cerevisiae* (P33334); Prp8Sp = *Schizosaccharomyces pombe* (O14187); PA En = Influenza virus PA endonclease (3HW4). R8Hm, DongFr, CRE2Cf, CZARTc, HERODr, HEROBf, NeSLTv, NeSLAc, UtopAm, and UtopCp sequences were collected from Repbase [69]. Holiday junction resolvases Ssol Hje (1ob8) and Ssol Hjc (1hh1) are from *Sulfolobus solfataricus*. Holiday junction resolvase Stok Hjc (2eo0) is from *Sulfolobus tokodaii* str. 7 Pfur Hjc (1gef) is from *Pyrococcus furiosus*

A multiple alignment and Ali2D secondary structure prediction is presented for the most conserved portion of the linker for RLE LINEs, APE LINEs, and Prp8 (Fig. 4a). Near the end of the linker region of RLE LINEs is the highly conserved IAP/gag-like CCHC zinc-knuckle motif with a spacing of CX_2-3_CX_7-8_HX_4_C (Fig. 4a). In R2Bm the IAP/gag-like CCHC zinc-knuckle is located at amino acids 863-883. The spacing of the cysteines and histidine in the motif is similar to that of IAP domains, although a bit smaller, or gag-knuckles, although a bit larger [48]. IAP domains form a ββα structure around zinc ion [48]. A gag-knuckle is β-strand followed by a knuckle (a sharp turn) with a less structured finish (e.g., coil with bends) [48]. The zinc ion is coordinated by the C and H residues of the motif [48]. The β-strands and α-helix are generally short. The canonical structure for an IAP domain is indicated above the R2Bm sequence listed in Fig. 4a as is the predicted (Ali2D) secondary structure for the linker region of RLE LINEs. For many RLE LINEs a short α-helix was predicted near the H residue (Additional file 7: S5). A β-strand was occasionally predicted near the first C residue using JPRED for RLE LINEs (data not shown). In many of the RLE LINE clades (e.g., R2, Dong, NeSL, and Utopia) there was a conserved R residue (R867) between the first two C residues.

APE LINEs, although lacking a downstream RLE, have a linker region that also ends with the IAP/gag-like CCHC zinc-knuckle. For APE LINEs, the area near the H was often predicted to be a β-strand.

It is not clear if Prp8 had a IAP/gag-like CCHC zinc-knuckle at one time or not. No CCHC motif exist in Prp8. Where the knuckle would be is not reliably aligned by sequence alignment programs to the LINE IAP/gag-like CCHC zinc-knuckle, or rather that there are several ways to align the region. In Fig. 4a, the CX_2-3_C (866-810 of R2Bm) of the zinc knuckle motif has been aligned with NRAN(1615-1620) in Prp8. In this configuration the R(1616) of Prp8 aligns to a conserved R found at the start of the zinc knuckle (R867 in R2Bm) in a number of the RLE LINEs. Residue 1620 (Y) in Prp8 then aligns with the conserved Y in Utopia (position 870 in R2Bm). In Prp8 there are two conserved H residues which could potentially line up with the H of the LINE CCHC motif: H1635 and H1652. In Fig. 4a we aligned the H1635 to the H of the LINE CCHC motif as H1652 was too close to the endonuclease. An alternative and perhaps better method of aligning Prp8 and LINEs in this area is by structure. Prp8 contains a non-zinc knuckle with a ββα structure that is positioned at 1624-1647. The Prp8 non-zinc knuckle might be a structural equivalent to the LINE IAP/gag-like CCHC zinc-knuckle.

In addition to the knuckle, there are two helices upstream of the knuckle in both Prp8 and LINEs that align well in sequence alignments. The two predicted α-helices upstream of knuckle in LINEs tended to be separated by LP. In R2 elements the sequence at the end of the first helix was highly conserved, being KXRINALP(840-847) or similar. In R2Bm the sequence was HTHINALP (see also reference [24]). The two helices prior to the knuckle appear to be present in both RLE and APE LINEs. In the APE LINE L1Hs, the sequence upstream of the gag knuckle that PROMALS3D aligned to the R2 KXRINALP was TMRYHLTP of HMKKCSSSLIAREMQIKTTMRYHLTP. The HMKKC is not shown in the alignment. Conversion of HMKK to AAAA and SSS to AAA reduced retrotransposition activity [5]. In the alignment SSS is below position 828 of R2Bm. A recombinant C-terminal 180 amino acid containing peptide (from SSS to the end of the ORF) bound RNA nonspecifically, but a mutation of the CCHC motif within the peptide did not affect RNA binding [49]. In the full-length protein, however, mutations of the conserved cysteines of the CCHC motif affected RNP formation and knocked out retrotransposition activity in cell culture assays [5, 50]. In Prp8, the non-zinc knuckle is predicted to make contact with mRNA in the U4/U6.U5 tri-snRNP complex [51].

The helices upstream of Prp8’s knuckle include an important loop (1585 loop, sometimes called the α-finger) that is important for binding RNA [51, 52]. The R2RLE LINE KXRINALP(840-847) helix equivalent in Prp8 was located at Prp8 residues 1592-1602 of the 1585 loop. The loop and helix region was found to be dynamic in Prp8 [21, 51–53]. In the U4/U6.U5 tri snRNP (cryo-EM structure 3JCM) the area forms a loop (QFKK, 1586-1589) plus an α-helix (HAQRTG, 1592-1597) [51]. The loop residues contact RNA and the Dib1 protein and were involved in branch point selection [51, 52]. After branching (cryo-EM structure 5LJ3), the area is not helical [54]. In the crystal structure (c4i43B), which lacks RNA, this area is unresolved and thus is likely unstructured [21].

RLE LINEs, like R2Bm, encode a restriction-like DNA endonuclease downstream of the IAP/gag-like CCHC zinc-knuckle (Fig. 4b). The DNA endonuclease found in RLE LINEs was found to have a fairly canonical αβββαβ restriction endonuclease-like fold, although it had a unique variant of the PD-(D/E)XK catalytic core [19]. The catalytic K, which is usually near the D/E residue in the third β-sheet, was found to be located much farther away in LINE RLE. The catalytic K in the LINE RLE is the first K in the KX_2_KY motif. The second K is less conserved across R2 elements and across RLE LINEs. The motif is located in the second α-helix [19]. The Y of the KX_2_KY, when mutated, also reduces cleavage [19]. The catalytic K in Prp8 is located in an identical position as the RLE of LINEs. The Y residue is also present in Prp8 and is identically positioned relative to the catalytic K. The second K of the LINE KX_2_KY is not present. The similarities between the Prp8 RLE and the LINE RLE go beyond the endonuclease fold and the positioning of the catalytic residues. At the far end of the endonuclease fold, just beyond the fourth β-strand, is a mutually conserved GXW motif. At the other end of the RLE fold—at the beginning of the first α-helix—is a conserved H residue and a conserved K residue. In R2Bm the equivalent is RH. Mutating the RH residues in R2Bm severely reduces DNA binding and DNA cleavage [19]. At the end of the first β-strand of both Prp8 and LINE RLEs is a conserved D/E that also appears to be unique to these two groups. Except for a 22 amino acid insertion between β-sheets 2 and 3 of Prp8, both Prp8 and LINE endonucleases are about the same size. The Prp8 endonuclease appears to have the amino acid residues needed for the cleavage activity, but the residues do not appear to be involved in metal coordination in the crystal structure; rather, these residues stabilize the polypeptide loop blocking the active site [21].

**Fig. 5:**
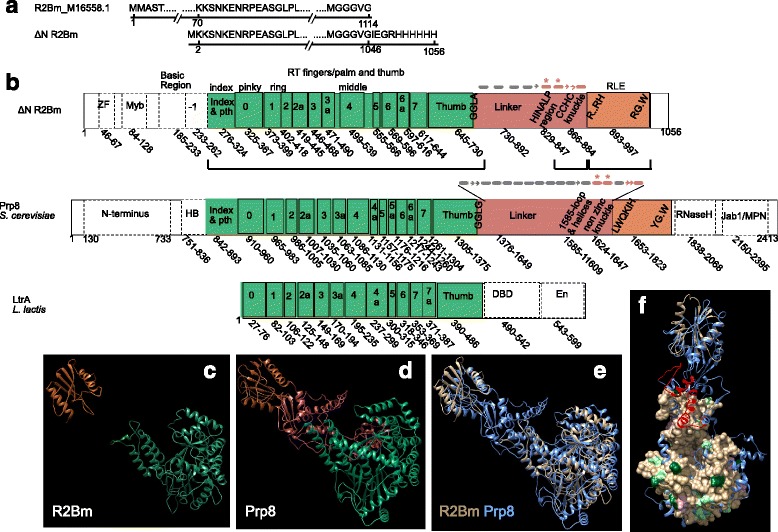
A Comparison of domain architechture of R2Bm, Prp8, and LtrA group II intron proteins. **a** The amino acid equivalences between ΔNR2Bm and that of the full length R2Bm ORF (genbank entry M16558.1) is such that the 2nd amino acid of the ΔNR2Bm ORF is 70th amino acid of full length R2Bm. **b** The ORF structure of ΔNR2Bm, *S. cerevisiae* Prp8, and *L. lactis* LtrA are presented as a colored bar diagrams. The RT is green, the linker is a maroon, and the RLE is orange. The RT sequence-motif blocks are indicated with approximate primary amino acid numbers. The locations of the fingers are also shown. The bar diagrams are not drawn to scale. The domains and numbering for Prp8 and LtrA group II intron are largely from [22, 52, 70, 71]. Brackets indicate regions of R2Bm that align well with corresponding region of Prp8. The RT and the RLE align well in sequence and by structure modeling with Prp8. In the linker, the sequence of (and around) the colored α-helices (rounded bars) with an asterisk align when well when anchored by the reverse transcriptase and the RLE. The colored β-strands and the α-helix lacking an asterisk do not align well but (may) form a knuckle that is structurally similar. The secondary structure is predicted in R2Bm, but known in Prp8 (3JCM). **c** Ribbon diagrams of the R2Bm RT and RLE models. The ribbons have been colored as in the the cooresponding bar diagram. **d** Ribbon diagrams of the Prp8 cryo-EM structure (3JCM, RT to RLE) is shown in matching color bar diagram. **e** Ribbon overlay of R2Bm (tan) with Prp8 large fragment (blue). **f** The R2Bm reverse transcriptase model as colored in Fig. 3e, a ribbon model of the R2Bm RLE and the large fragment of Prp8 (blue with red 1585 loop and knuckle). Abbreviations: palm traversing helix (pth); DNA binding domain (DBD); Endonuclease (EN). All other abbreviations and symbols are as in previous figures

Comparative bar diagrams comparing R2Bm, Prp8, and LtrA ORF structure are presented in Fig. 5b. The RT, Linker, and RLE are highlighted in green, maroon, and orange, respectively. The areas of the ORF that have been aligned, homology modeled, or deemed structurally equivalent are also indicated. The secondary structure present in the linker of R2Bm and Prp8 is depicted. The areas of the linker that align in sequence (HINALP/1585-loop regions) or by structure (knuckle) are indicated in maroon. Ribbon diagrams are also given for RT-RLE for R2Bm (Fig. 5c) and Prp8 (Fig. 5d) using the same color scheme as the bar diagrams. A structural overlay (ribbon diagrams) of R2Bm RT and RLE onto Prp8 RT-RLE is presented in Fig. 5e. A second overlay between R2Bm and Prp8 is presented in Fig. 5f in which the R2Bm RT is a surface model and is colored an in Fig. 3e. Also colored in Fig. 5f is the Prp8 non-zinc knuckle and the 1585 loop region (both red). The Prp8 knuckle and 1585 loop regions are positioned near the top of the thumb. The knuckle also is positioned near the RLE. It sits between the thumb and RLE. The 1585 loop is oriented toward the fingers of the RT. The R2Bm RT equivalents (HINALP region and knuckle) may also be closely associated with the thumb. Having the linker associated with the thumb would explain, in part, why the thumb is protected from cleavage.

## Discussion

The R2Bm protein was found to be comprised of two major globular domains: the ZF/Myb/−1 N-terminal domain and the RT/linker/RLE superdomain. The index finger of the RT and the −1 region were the most accessible areas for protease cleavages to occur, indicating that these regions might represent flexible conformational-switch areas that may help coordinate the nucleic acid binding and cleavage activities of the two globular domains. The ZF/Myb/−1 region is present among all of the early branching LINE elements with a variable number of ZFs and Myb motifs [55]. The primary variability in the RT/linker/RLE superdomain was in the linker, with Cre elements often having a deletion relative to R2 and Utopia having an insertion. The linker was predicted to be largely α-helical across all RLE LINEs and, we hypothesize, closely associated with both the RT thumb and the RLE, similar to Prp8. The linker of R2 contained several highly conserved sequence motifs and secondary structures, most notably the presumptive-ββα-forming IAP/gag-like CCHC zinc-knuckle. Just upstream of the knuckle in R2 elements are several well aligned and predicted α-helices separated by the highly conserved KXRINALP(840-847) motif. The two appear to be present in both RLE and APE LINEs. Mutations in this region affected retrotransposition in L1Hs [5, 50].

The linker of Prp8 was also found to have an non-zinc knuckle structure and an upstream dynamic loop plus helix region (1585 loop region) important for interacting with nucleic acids [51–53]. In Prp8 the 1585 loop region sits on top of the RT thumb and is oriented toward the fingers of the RT. It would appear tha the region immediately upstream of the knuckle in RLE LINE, APE LINEs, and Prp8 might be structurally conserved and, to a degree, functionally conserved. If the helices preceding the knuckle in LINEs were positioned as in Prp8, it is easy to envision the region participating in binding to element RNA or target DNA.

The gag knuckle-like motif and associated upstream helices might promote switching between polymerase active and endonuclease active conformations of the R2 protein in response to binding insertion reaction intermediates. Our proteolysis study was done in the absence of nucleic acids. In the absence of RNA, the R2 protein would be expected to adopt a conformation that would have characteristics of the conformation involved in second-strand cleavage. It is possible that in the presence of RNA or DNA our results would differ from those presented as the nucleic acid might block some sites from being cleaved while presenting other newly accessible sites due to protein conformational changes induced by nucleic acid binding.

The RT of Prp8 has been noted to share similarities to RdRP and to the RTs encoded by mobile group II introns and LINEs [26, 34]. However, because Ppr8 and the group II intron protein both function as splicing maturases, the similarity to group II introns has been stressed. The RLE LINE RT, however, appears to be more similar to Prp8 given the presence of an index finger and a palm traversing helix although our phylogenetic studies (not reported) using the RT were inconclusive. In addition to the RT both Prp8 and RLE LINEs have a RLE. While it has been noted in the literature that the large fragment of Prp8 contains an RLE, the connection to LINEs had not been presented beyond noting that LINEs also contain an RT and an RLE. Using sequence and structure comparisons, we may have been able to infer insights that are not yet forthcoming in phylogenetic trees. In this paper, we have shown that the LINE RT, linker, and RLE share more points of commonality to the large fragment of Prp8 than does the group II intron maturase.

## Conclusions

The protein encoded by RLE LINEs was shown to consist of two major globular domains. The larger of the two globular domain contained the RT, linker, and RLE and was found to be similar to the large fragment of the spliceosomal protein Prp8. The RLE, RT, and linker of LINEs and Prp8 shared a greater degree of structural and sequence similarity to each other than to the maturase of mobile group II introns.

## Methods

### Protein expression and purification

R2Bm protein was expressed and purified as previously described [19]. Briefly, the R2Bm protein used in this study was ΔNR2Bm. The ΔNR2Bm construct removes the variable N-terminal found in R2 elements (amino acid 2 of ΔNR2Bm = amino acid 70 of genbank entry M16558.1) and adds a six histidine tag on the C-terminal end of the protein [19]. The ΔNR2Bm expression construct was put into BL21 *Escherichia coli* cells. Five hundred milliliter cultures were grown in LB broth, expressed with IPTG, lysed, and the soluble material purified over a Talon affinity column (Clontech #635501). The R2Bm protein was eluted off the column in 50 mM HEPES pH 7.5, 100 mM NaCl, 50% glycerol, 0.1% triton X-100, 150 mM imidazole. Proteins were stored in elution buffer supplemented with 1 mM DTT (final concentration) at −20 °C. R2 protein was quantified by SYPRO Orange (Sigma #S5692) staining of samples run on SDS-PAGE relative to a BSA standard (Biorad #500-0202). All quantitations were done using Fiji software analysis of digital photographs [56].

### Limited proteolysis of R2Bm protein and processing of the polypeptides

Limited digestion of purified R2 protein was carried out in the absence of nucleic acids using a trace amount of GluC (NEB, #P8100S) or LysC (Promega, #V1671) protease. Digestion was stopped using SDS loading buffer (to a final concentration of 50 mM Tris-Cl, pH 8.8 final; 4% SDS; 10 mM DTT) and heated. Proteolytic fragments were carbamidomethylated (55 mM final, Alfa aesar, #A14715) in the loading buffer in the dark at room temperature for 30 min prior to loading onto a precast (Biorad criterion polyacrylamide gel, 18% and 4-15%) SDS-PAGE gel [57]. The resolved protein fragments were stained with colloidal coomassie blue (Invitrogen, #LC6025). Prominent bands from across the proteolytic time course were excised from the gel, cut into 1 mm pieces, and destained using 25 mM NH_4_HCO_3_/50% Acetonitrile (ACN). Gel pieces were shrunk with 100% ACN (VWR, #BDH6002-4) and dried by Speed Vac (Eppendorf) [58–60].

The primary amines, including the amino-terminal end of the proteolytic fragments, were acetylated in the gel slice using 15% acetic anhydride (Sigma, #320102) for five hours at room temperature within the individual excised gel fragments [61, 62]. Acetylation was stopped by adding 1 M NH_4_HCO_3_ (Sigma, #40867) solution [61, 62]. After 20 min, the gel pieces were shrunk by 100% ACN.

The dried gel pieces were swelled in 25 mM NH_4_HCO_3_ containing trypsin or GluC for 1.5 h at 4 °C and any unabsorbed NH_4_HCO_3_ solution was then discarded [58–60]. The gel pieces were covered with 25 mM NH_4_HCO_3_ and the in-gel digestion was carried out overnight at 37 °C. Peptides from in-gel digestion reaction were collected in the supernatant. Additional extractions with 0.1% formic acid (FA) (Sigma, #399388) and 50% ACN/0.1% FA were also collected and added to the supernatant [58–60]. The supernatant was dried in a Speed Vac and purified over C18 zip tip using standard procedures [58–60].

To catch any major cleavage sites that did not result in isolatable SDS-page bands, limited proteolysis reactions were run on an SDS-PAGE gel for a very short time so as to not resolve bands, rather keeping them clustered near the well. The top portions of these lanes were excised and processed as above so as to remove triton and otherwise prepare the polypeptides for mass spectrometry, thus avoiding the precipitation of the larger R2Bm protein fragments that occurs if the polypeptide processing (for mass spectrometry) was done in solution instead of in-gel. This abbreviated in-gel procedure is roughly equivalent to a direct “in-solution” detection of cleavage sites.

### Mass spectrometry and Edman degradation

The eluted peptides were resuspended in 0.1% FA for sequencing by nanoLC-ESI-MS/MS using a Thermo Scientific LTQ Velos Pro ion trap mass spectrometer. R2 peptides were identified using Thermo Proteome Discoverer software (version 2.0); a database of R2Bm protein fragments was created, and a peptide was assigned as either N-terminal end or internal peptide based on the position of acetyl groups in the peptide sequence [61]. The internal peptides generated after trypsin (second) digestion will lack an acetyl group at the N-terminal end, as acetylation is performed prior to the second protease digestion step.

Amino-terminal sequencing of the separated proteolytic fragments was used to map the protease cleavage sites back onto the primary sequence of R2 and thus delimit globular domain boundaries. The internal peptides were also identified from the MS/MS spectrum. The internal peptide coverage and sequence were used to help verify the peptide location within the R2 ORF and to act as a rough estimation of the C-terminal boundary of the fragment, along with SDS-PAGE estimation of the fragment’s molecular weight. The Glu-C cleavage heatmap was generated using Gitools [63].

For detection by Edman degradation, an SDS-PAGE gel was electrophoresed onto a PVDF membrane. Excised bands on the PVDF membrane were sent to UT Southwestern proteomics core for Edman sequencing.

### 3D modeling and multiple sequence alignments

The Phyre 2.0 protein fold recognition server was used to model the RT domain of R2Bm protein [25]. The intensive mode with default parameters of Phyre 2.0 were used. Different lengths of R2Bm sequence upstream and downstream of RT domain were submitted for modeling to find the sequence window that modeled the best. Model visualization was aided by UCSF Chimera package [64].

The PROMALS3D server was used for structure based alignment with minor manual adjustments [65–67]. Seventy five LINE sequences were aligned first in PROMALS3D server that included 31 RLE LINE and 44 APE LINEs. Using this LINE alignment as constraint, an extended alignment was built with three nMat proteins, five group II introns, seven RVT genes and 10 Prp8 proteins. The secondary structure was plotted on the multiple sequence alignment using the Ali2D program of the MPI bioinformatic tool kit [67, 68].

## Additional files

Additional file 1: Lys C mapping data. (ZIP 983 kb)

Additional file 2: R2Bm sequence and domain boundaries. (DOCX 28 kb)

Additional file 3: Glu C mapping data (amino terminal identification). (PDF 287 kb)

Additional file 4: Glu C mapping data (internal peptides). (PDF 541 kb)

Additional file 5: List of Glu C and Lys C sites with early vs. later designations. (XLSX 482 kb)

Additional file 6: Pdb file of R2Bm RT model. (PDB 307 kb)

Additional file 7: Alignment files. (ZIP 5764 kb)

## Abbreviations

APE: Apurinic-apyrimidinic family endonuclease
LINE: Long interspersed element (also called non-LTR retrotransposon)
ORF: Open reading frame
RLE: Restriction-like endonuclease
RNP: Ribonucleoprotein particles
RT: Reverse transcriptase
TPRT: Target primed reverse transcriptase
