# Supplementary material for: Globular domain structure and function of restriction-like-endonuclease LINEs: similarities to eukaryotic splicing factor Prp8
Source: Mob DNA. 2017 Nov 7;8:16. doi: 10.1186/s13100-017-0097-9 (PMC5678591; doi:10.1186/s13100-017-0097-9)

Supplementary data S1A

Globular Domain Structure and Function of Restriction-Like-Endonuclease LINEs: Similarities to Eukaryotic Splicing Factor Prp8

M. Murshida Mahbub[1], Saiful M. Chowdhury[2]*, and Shawn M. Christensen1*

Figure: MS/MS spectra for N-term end peptides of LysC bands. The MS/MS spectra of peptide ions were matched to corresponding hypothetical sequences. Individual matched y-ions are indicated in blue and b-ions are indicated in red. The x-axis units are m/z and the y axes represent relative abundance normalized to the most intensive fragment ion.

LA(i) n-term end peptide = R*RAEYARVQE; z = 3+;
XCorr = 2.34; #PSMs= 3; [M+3H]3+ = 440.70; Theo. [M+H]1+ = 1319.6814; * = acetyl

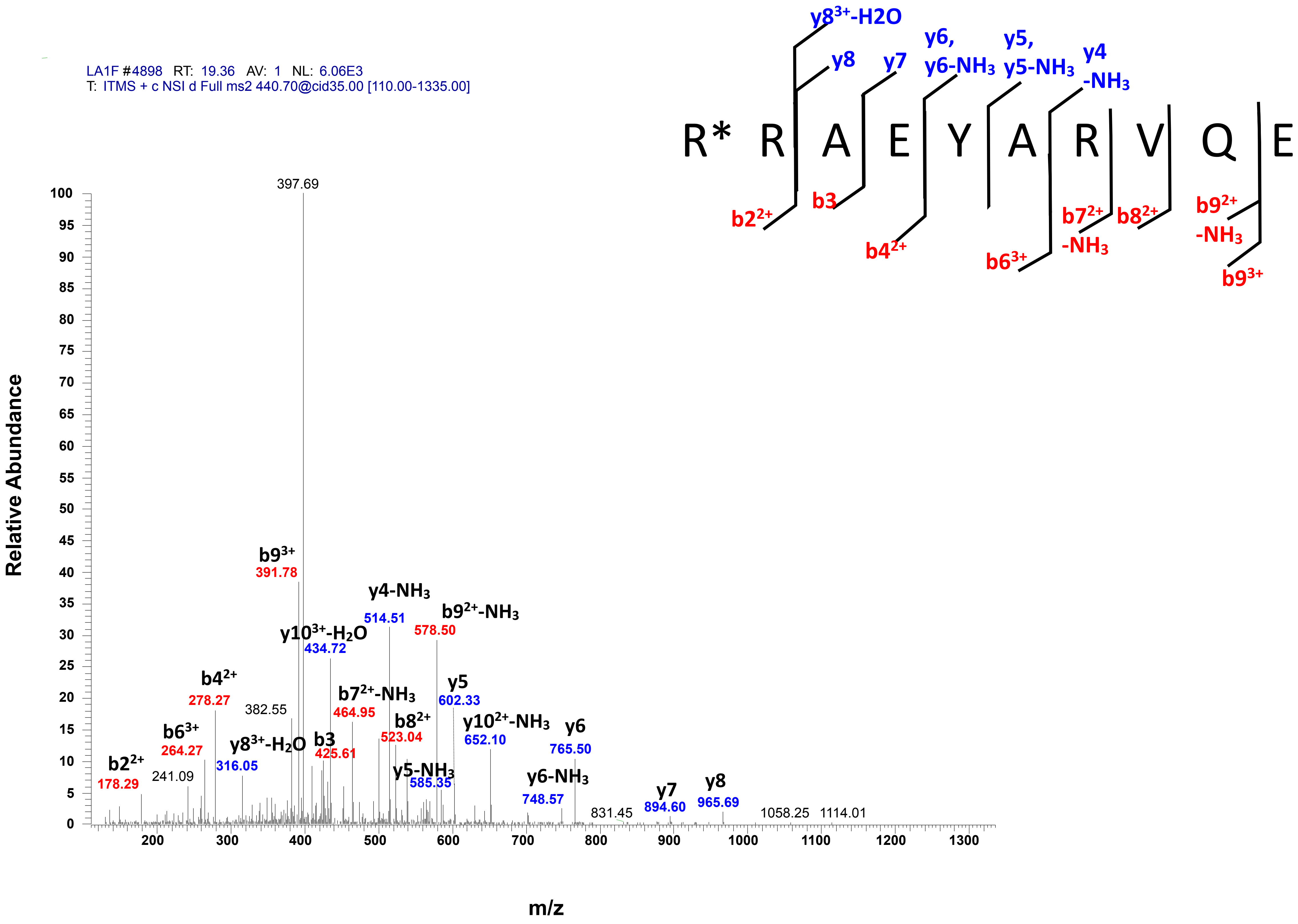

LA(ii) n-term end peptide = C!*RSRAAAE; z = 2+; XCorr = 2.72; #PSMs= 1; [M+2H]2+ = 481.59; Theo. [M+H]1+ = 962.44 ; * = acetyl; ! = carbamidomethyl

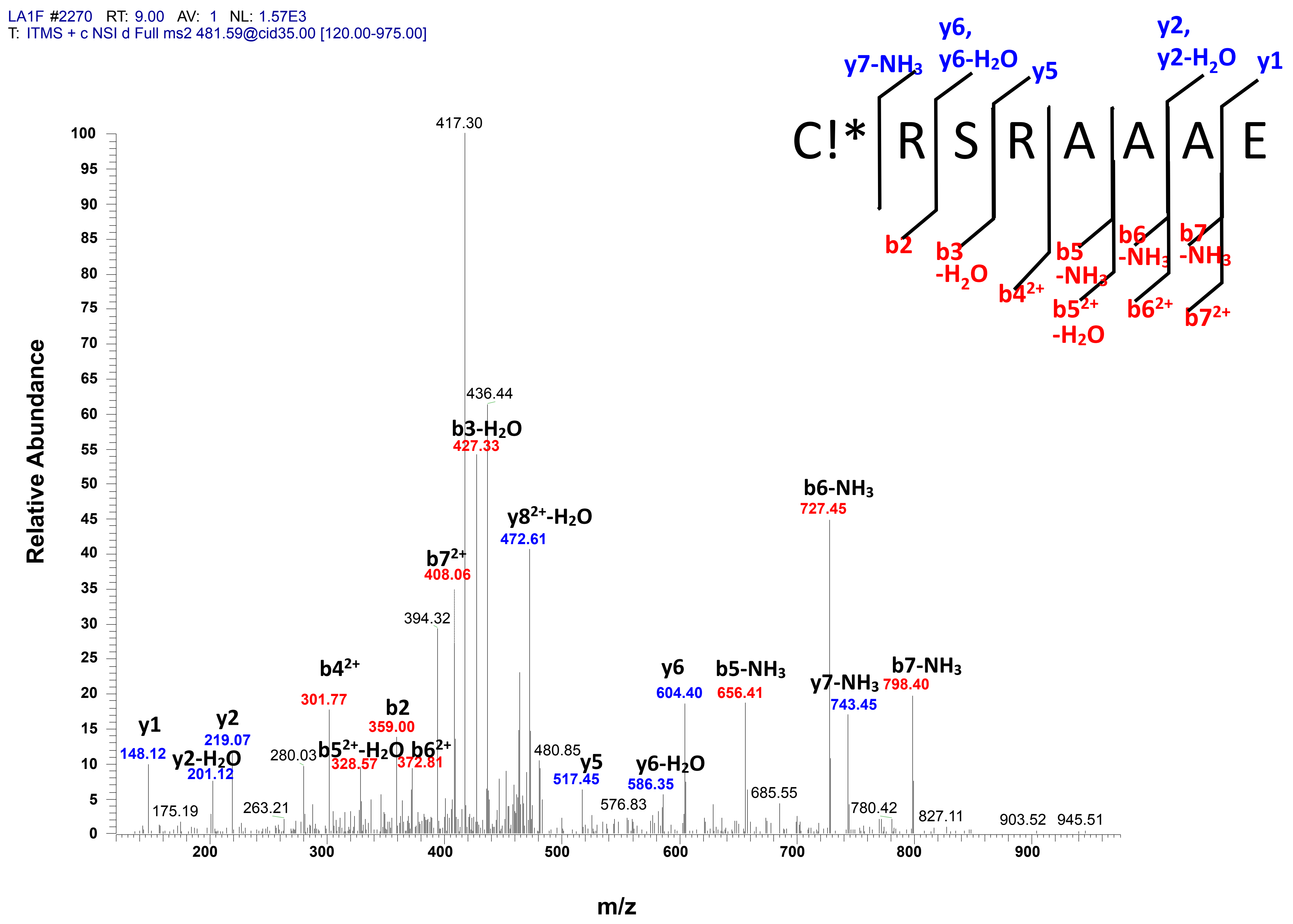

LB n-term end peptide = V*GM@QESISAVDC!VGR; z = 2+;
XCorr = 2.58; #PSMs= 1; [M+2H]2+ = 833.38; Theo. [M+H]1+ = 1665.75 ; * = acetyl; @ = oxidation; ! = carbamidomethyl

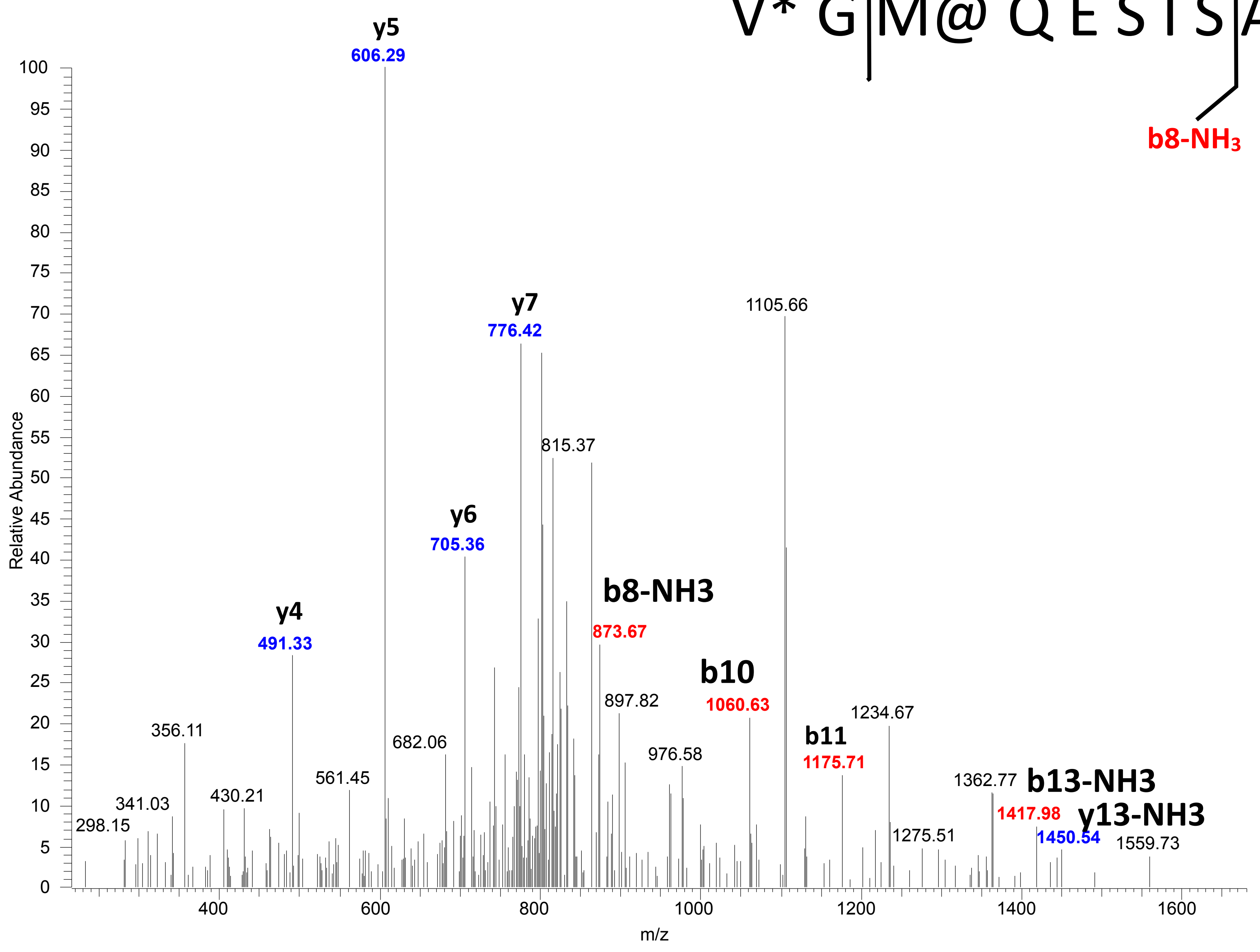

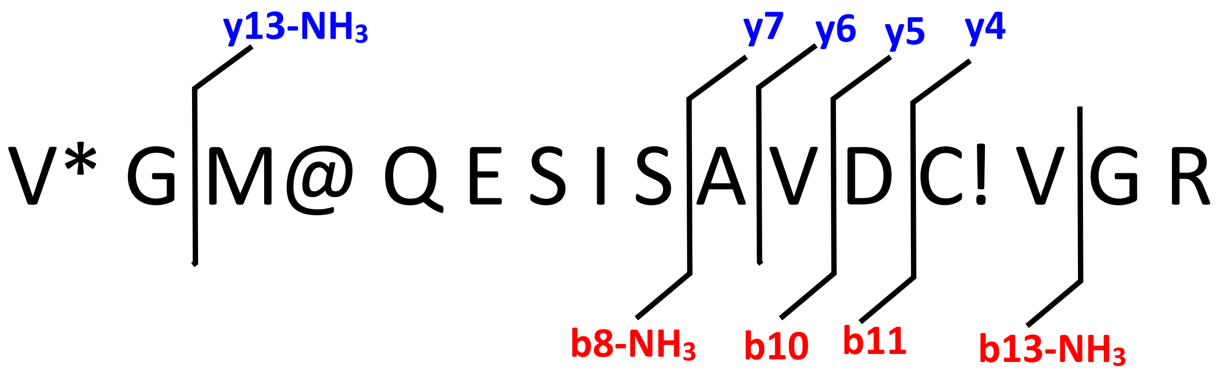

LC(i) N-term peptide: S*AVLSM@IPDGHR; z= +2; Xcorr = 3.22; Theo. [M+H]1+ = 1340.6521; [M+2H]2+ = 671.08; #PSMs = 3; * = acetyl; @ = oxidation

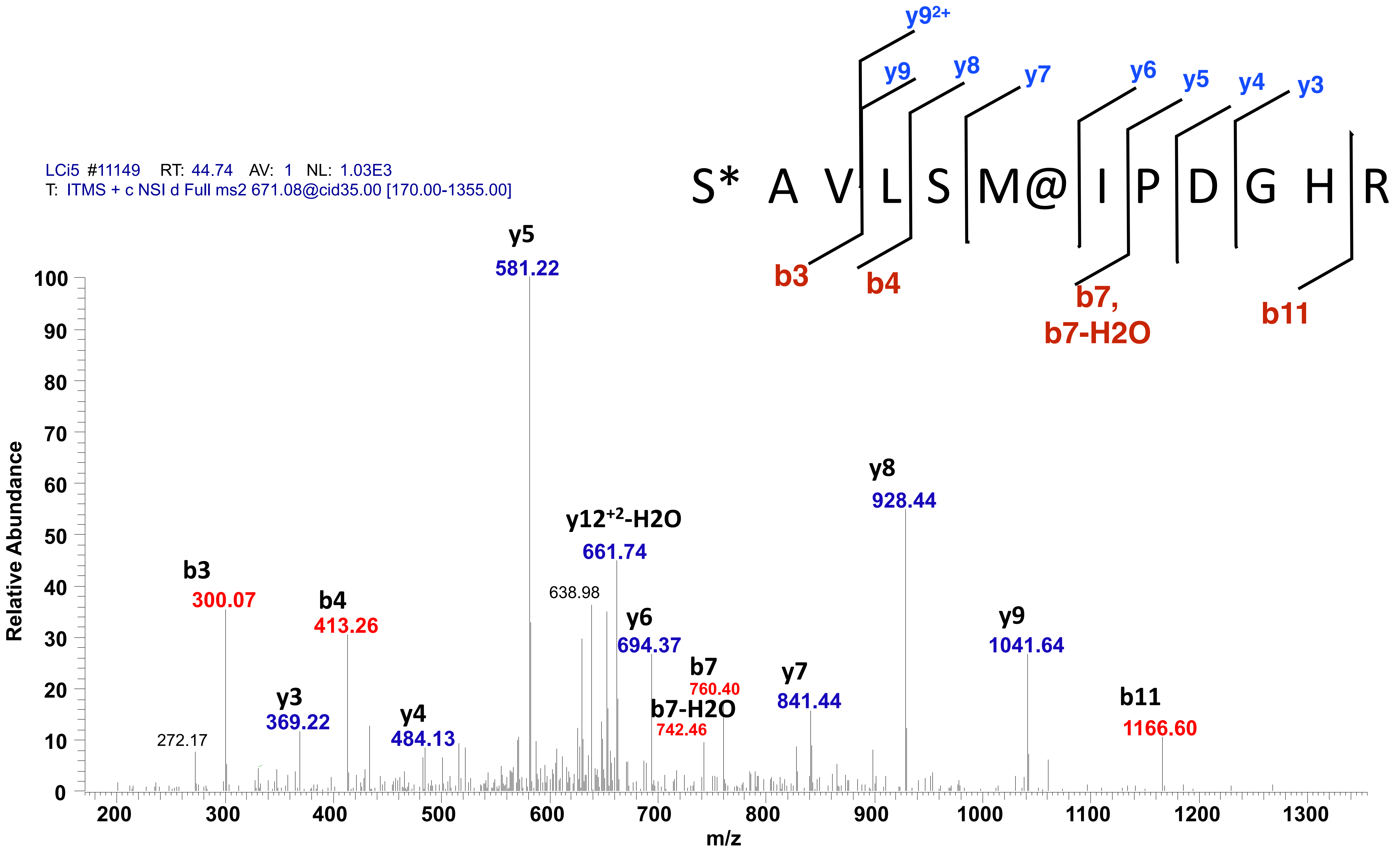

LC(ii) N-term peptide: H*HYLTER; z= +2; Xcorr = 2.067; Theo. [M+H]1+ = 997.4744; [M+2H]2+ = 499.44; #PSMs = 2; * = acetyl

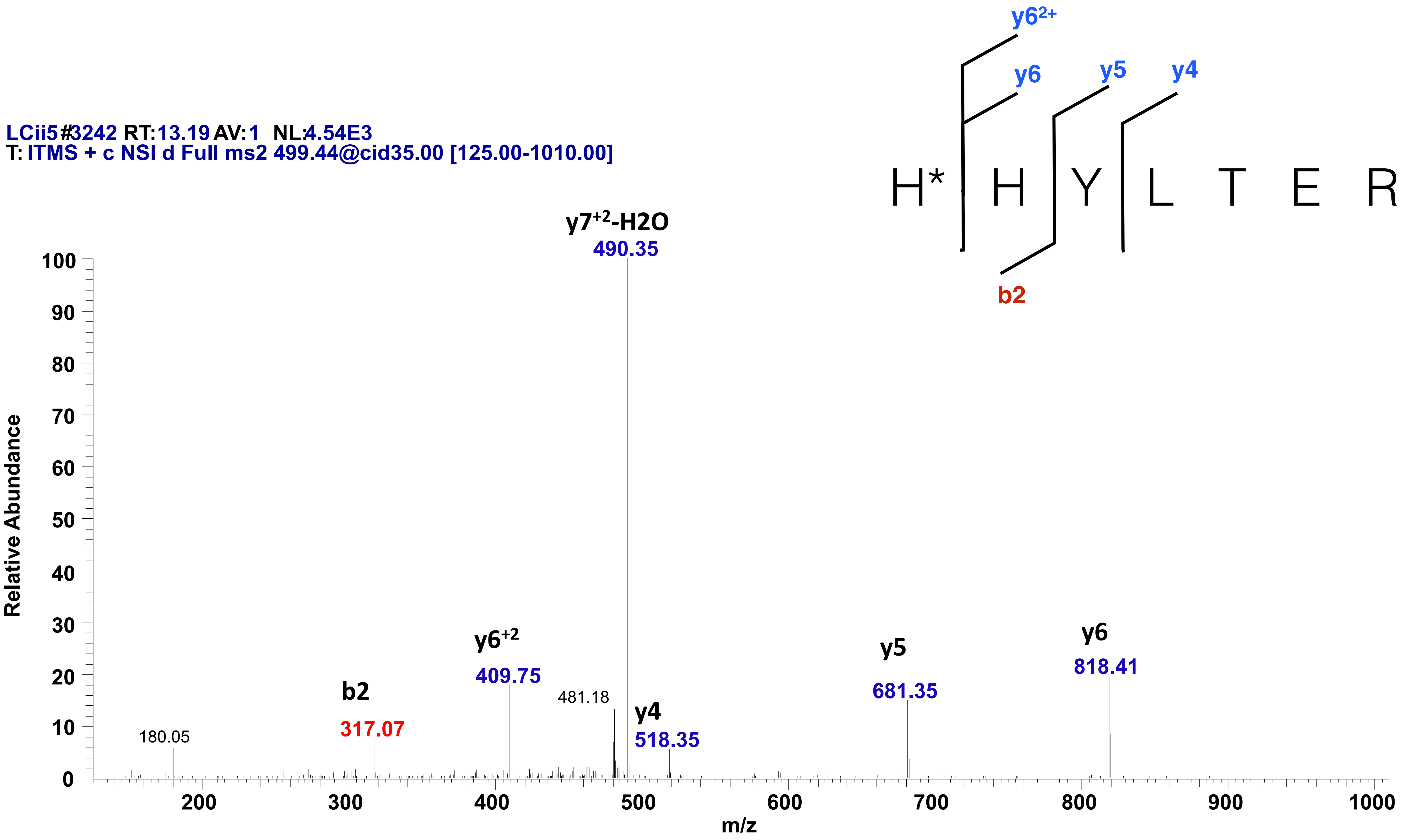

LD(i) N-term peptide:V*ERPGGPGEYRPISIASIPLR; z= +3; Xcorr = 3.08; #PSM = 1;
Theo. [M+H]1+ = 2305.24; [M+3H]3+ = 769.49; * = acetyl

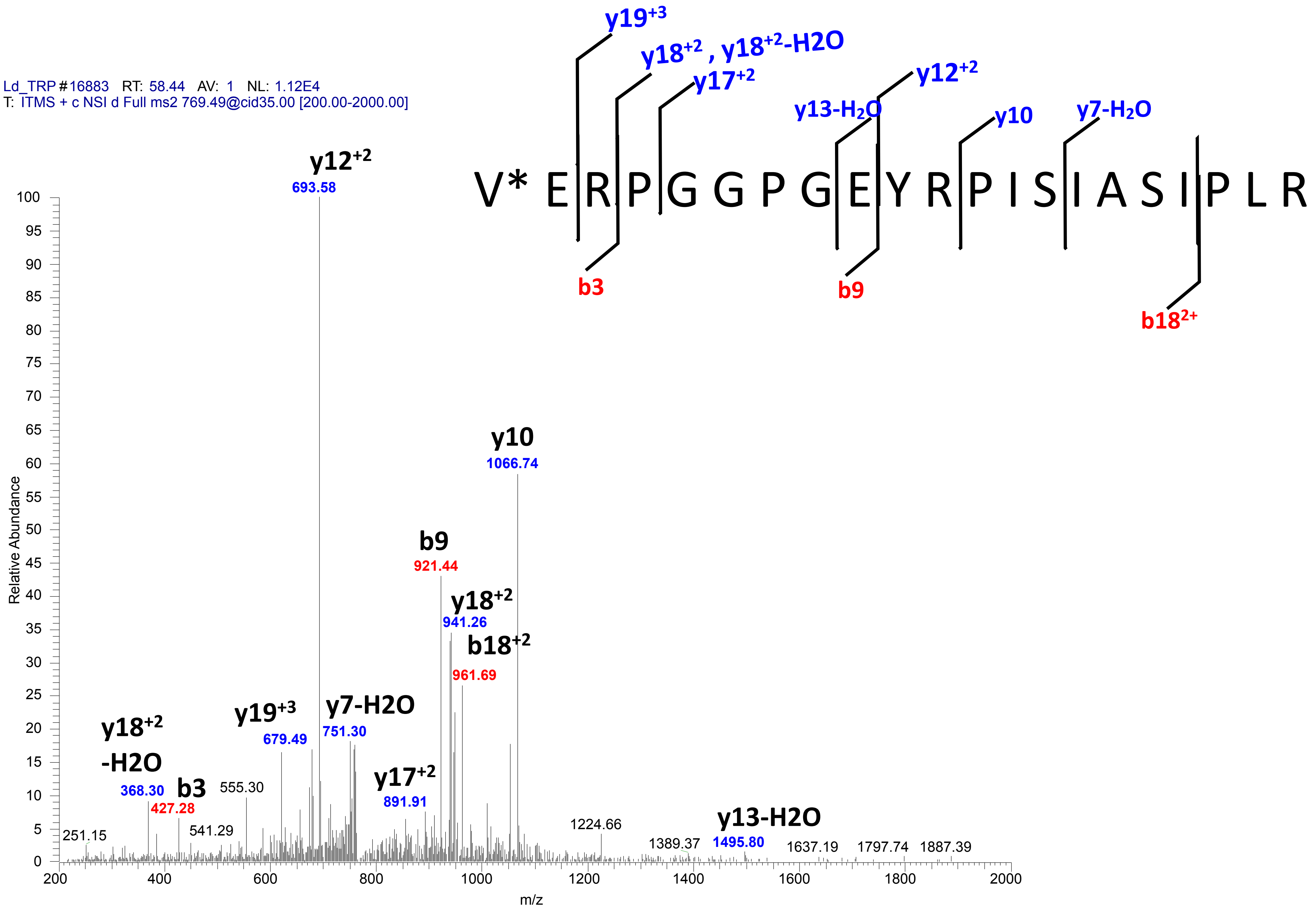

LD(ii) N-term peptide: A*YYHAAVQDGGLAIPSVR; z= +2; Xcorr = 6.08; #PSM = 3 Theo. [M+H]1+ = 1929.97; [M+2H]2+ = 965.67; * = acetyl

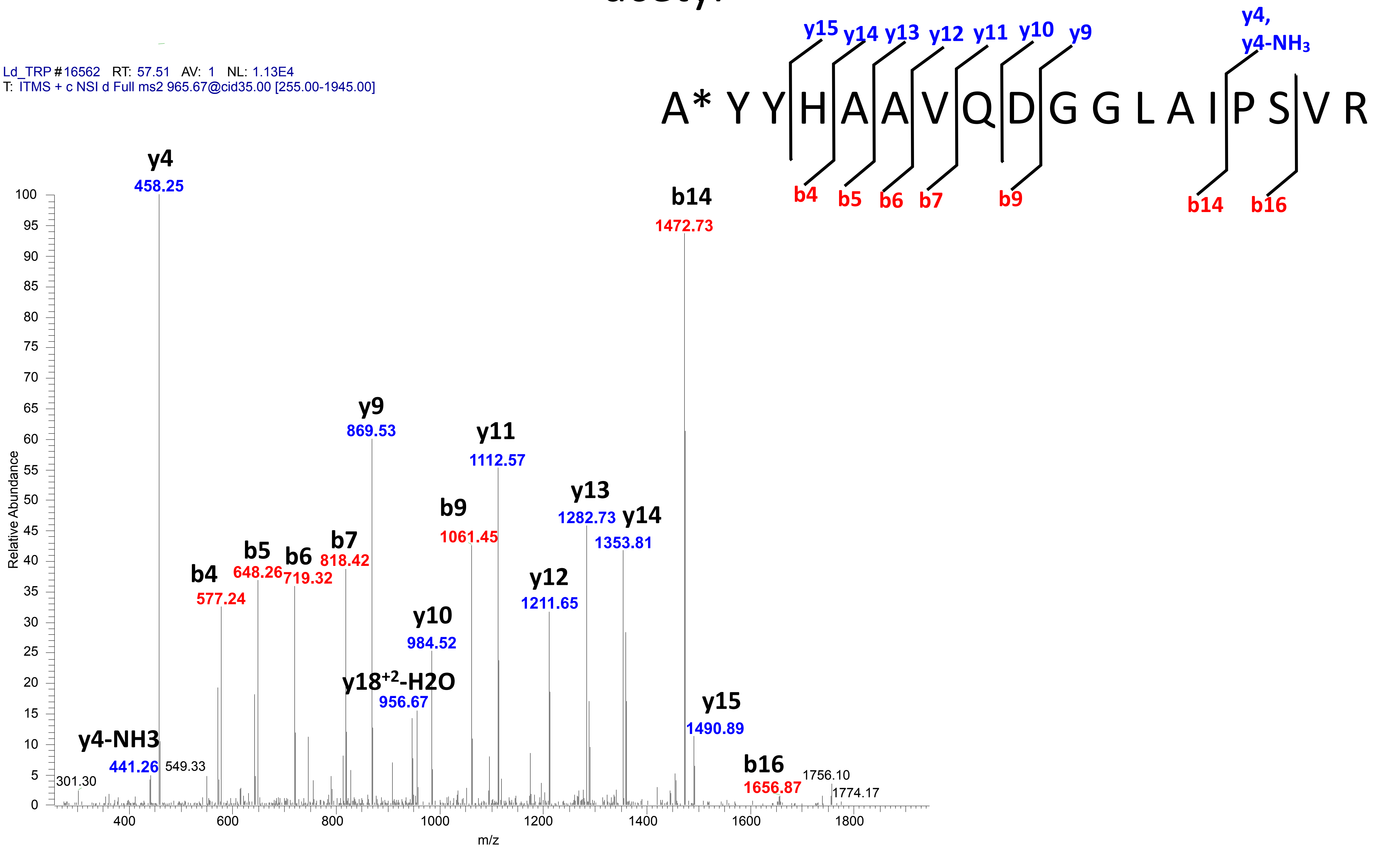

LE N-term peptide: S*DK*IRK; z= +2; Xcorr = 1.505; Theo. [M+H]1+ = 830.4518; [M+2H]2+ = 415.14; #PSMs = 3; * = acetyl

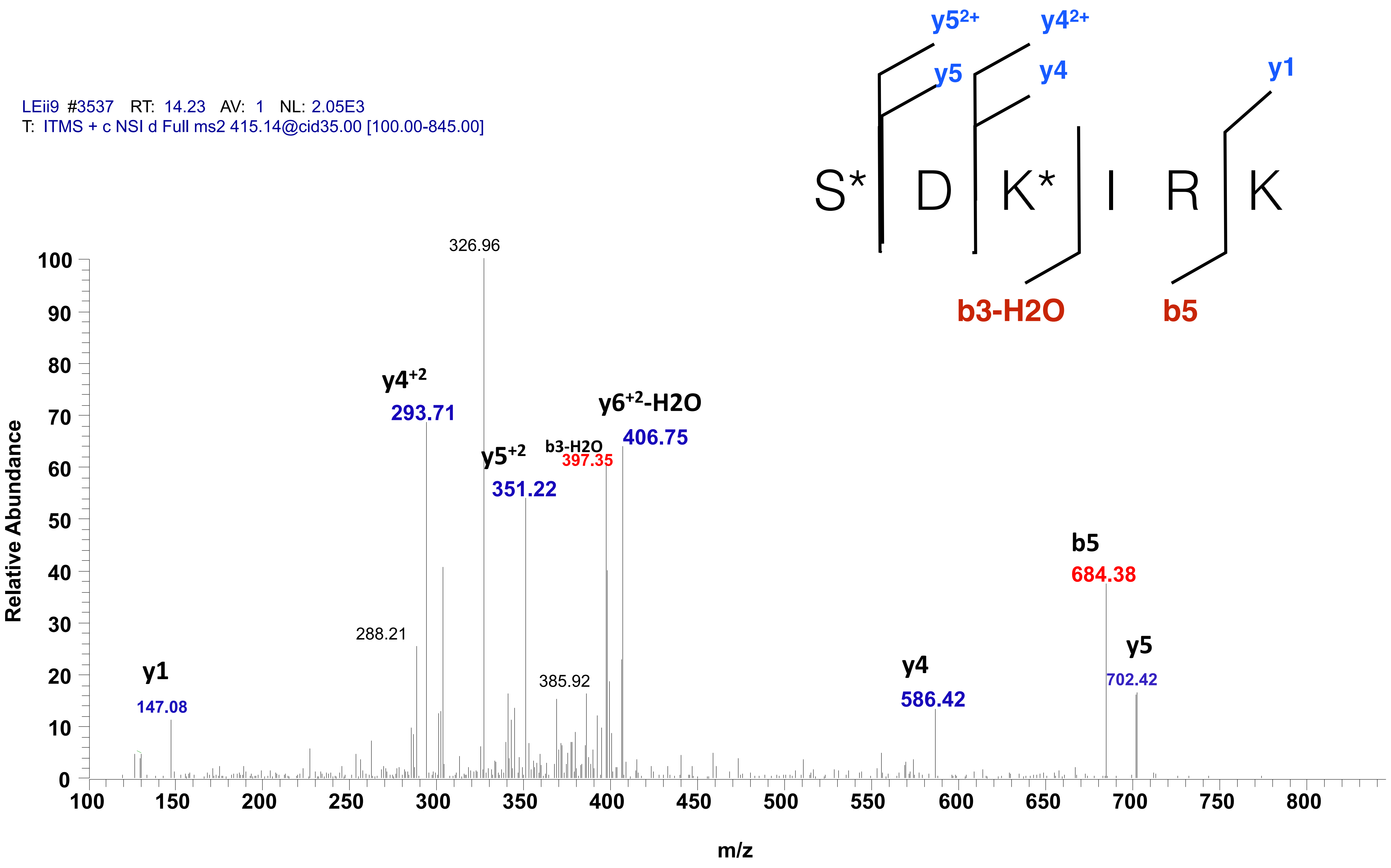

LF (i) N-term peptide: S*NK*ENRPEASGLPLESER; z= +2; Xcorr = 5.4277; Theo. [M+H]1+ = 2096.9995; [M+2H]2+ = 1049.20; #PSMs = 17; * = acetyl

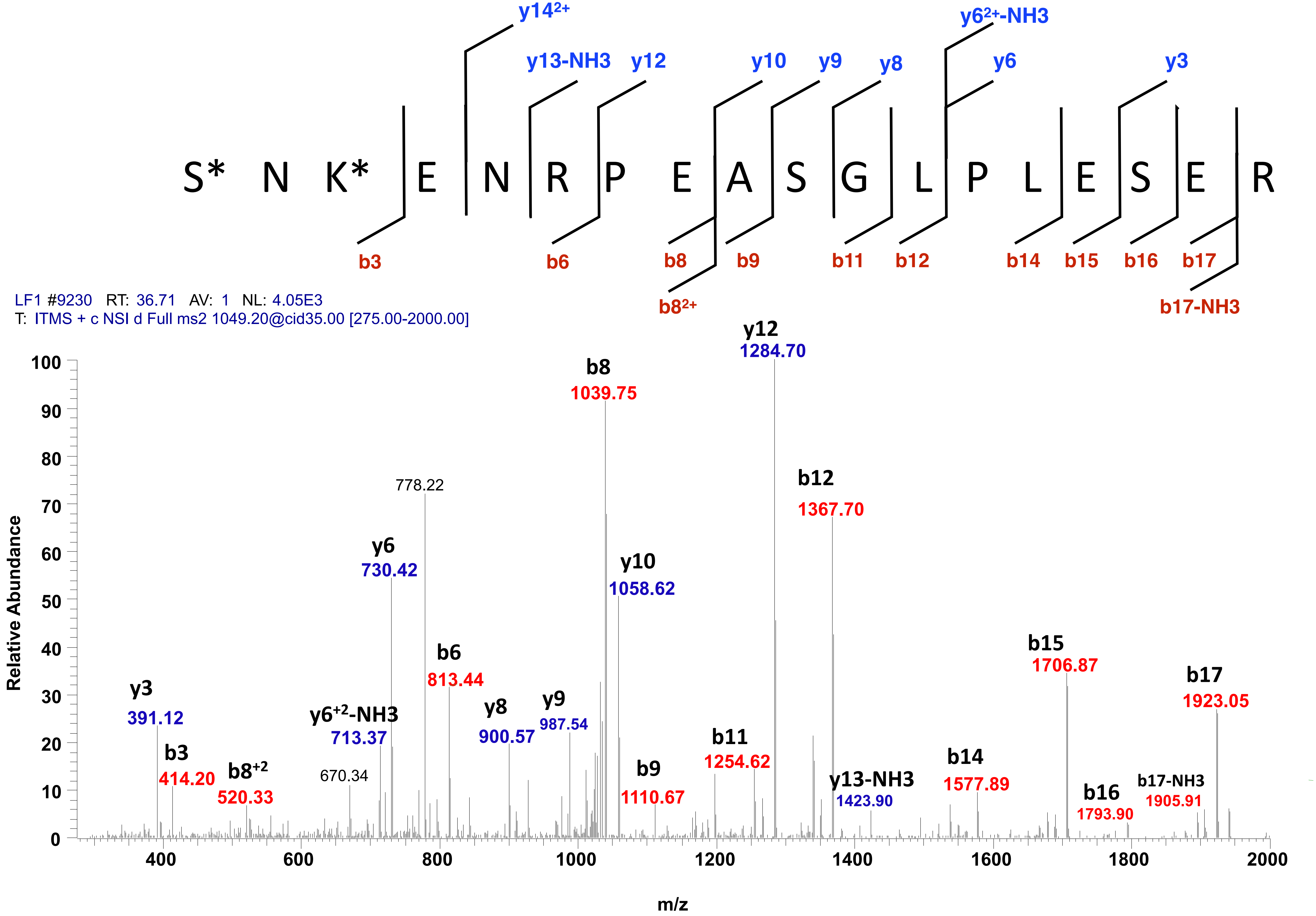

LF(ii) N-term peptide: E*NRPEASGLPLESER; z= +2; Xcorr = 4.09; Theo. [M+H]1+ = 1725.8296; [M+2H]2+ = 863.62; #PSMs = 8; * = acetyl

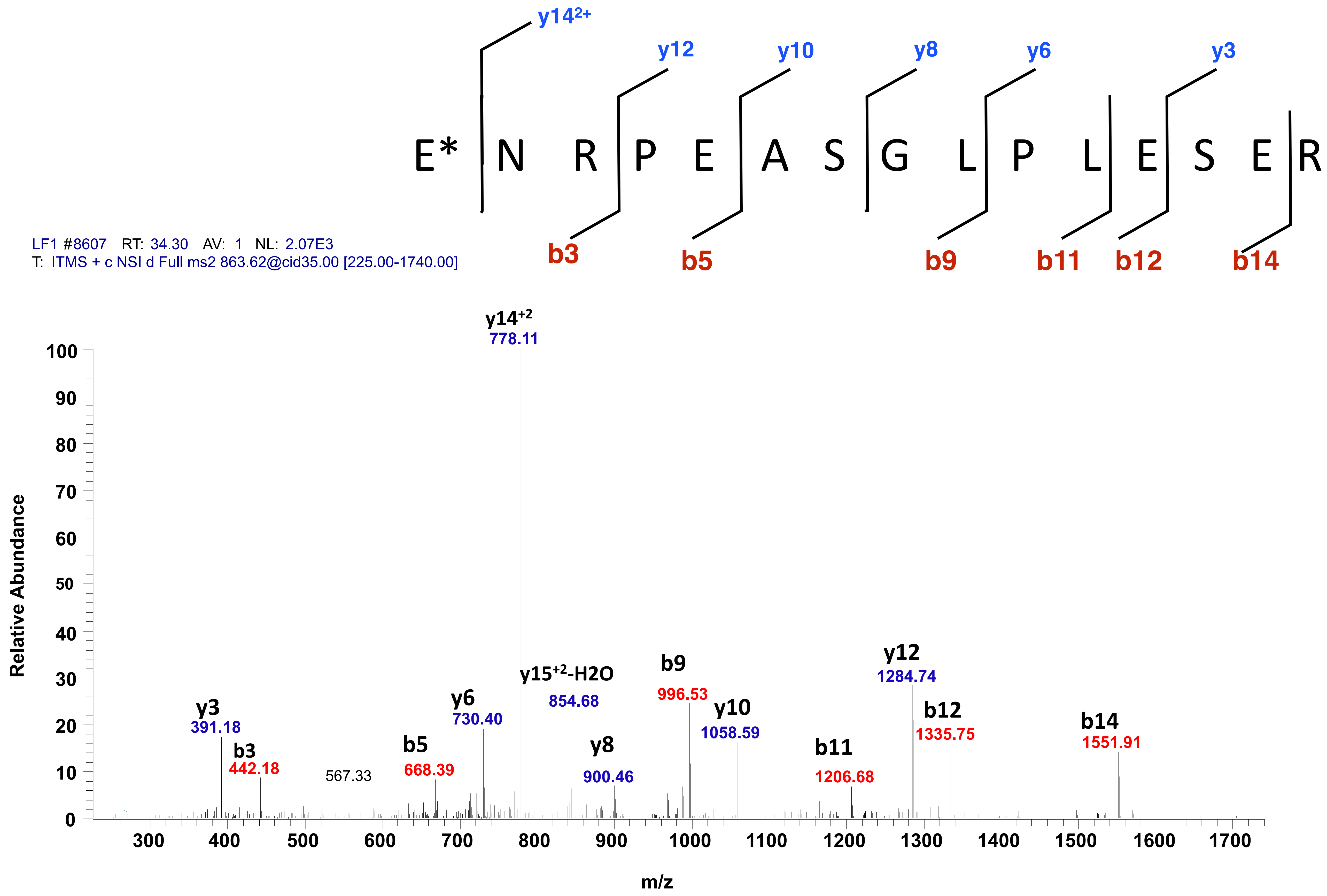

Band LG N-term peptide: R*RAHPVE; z= +2; Xcorr = 1.14; #PSMs = 1; Theo. [M+H]1+ = 906.48; [M+2H]2+ = 453.72; * = acetyl

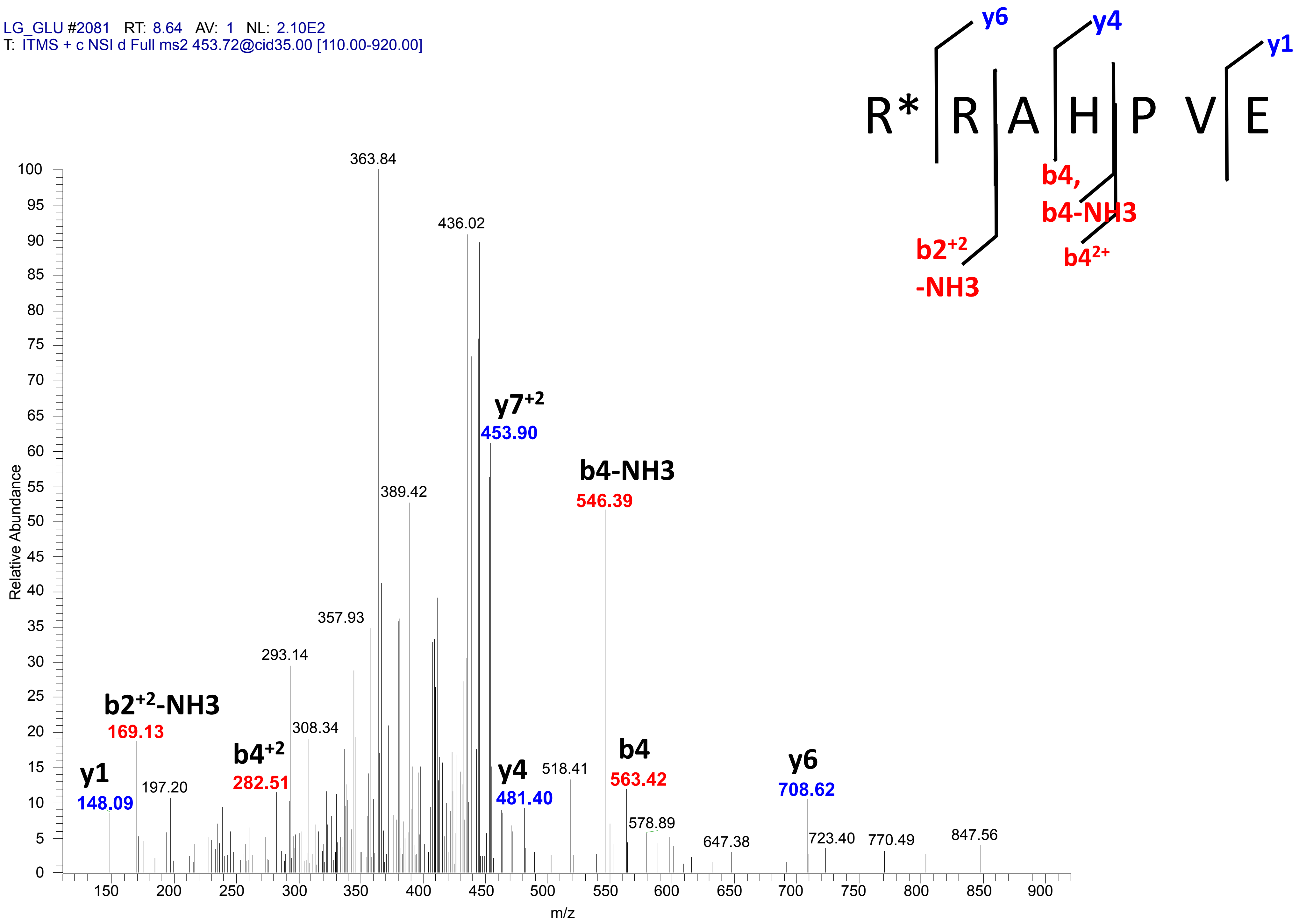

The fragmentation show less confidence but there are two arginines and one histidine in the peptide sequence. Ammonia loss is seen as expected. The internal peptide sequence coverage also validates this peptides as N-terminal end of Band LG.

LH N-term peptide: R*RWHGEE; z= +2; Xcorr = 1.65; Theo. [M+H]1+ = 1011.46; [M+2H]2+ = 506.22; #PSMs = 3; * = acetyl

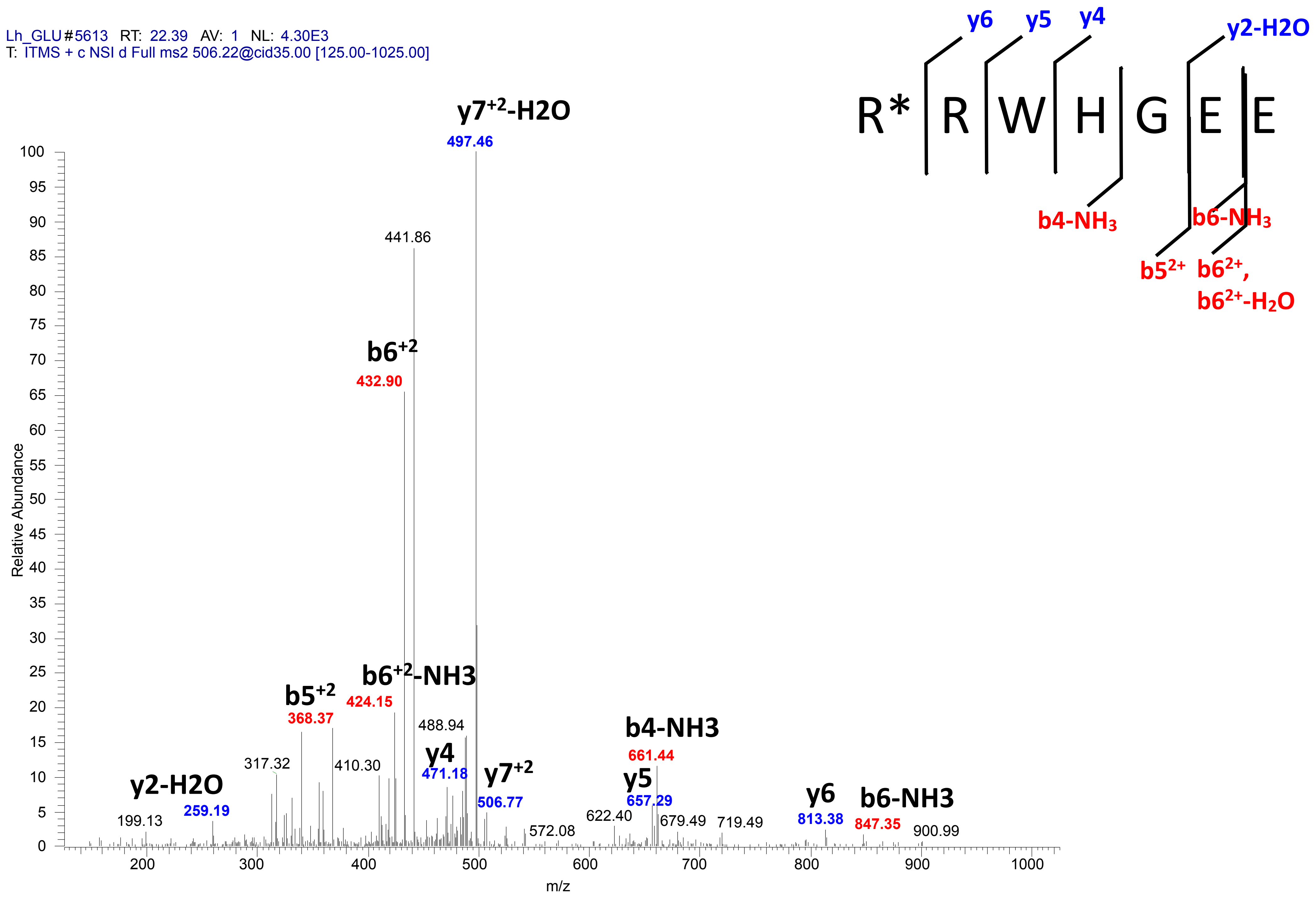

LI N-term peptide: H*HYLTER; z= +2; Xcorr = 2.07; Theo. [M+H]1+ = 997.47; [M+2H]2+ = 499.12; #PSMs = 3; * = acetyl

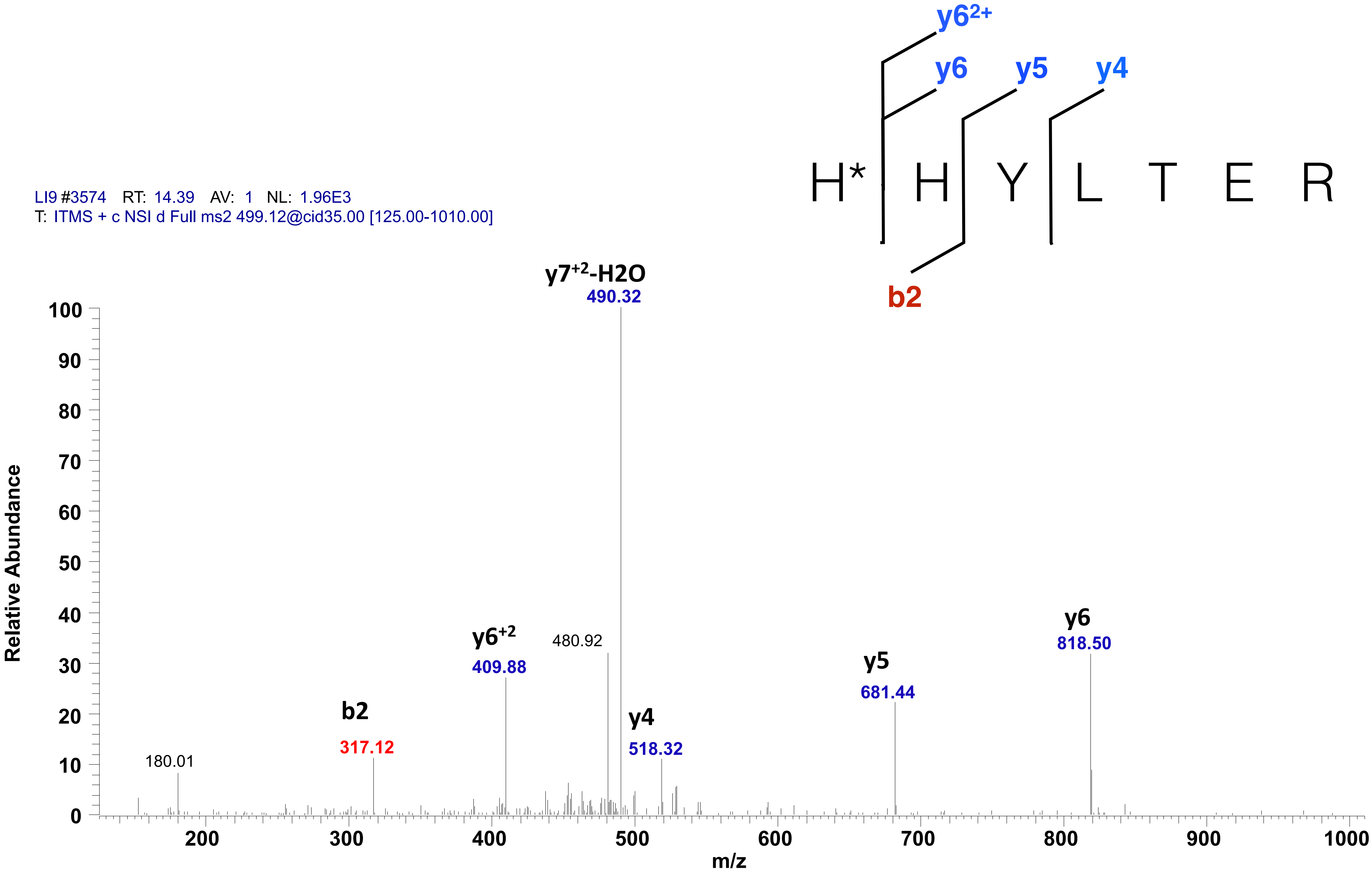

Supplement: Supplementary file 1 — Lys C mapping data. (ZIP 983 kb) [file 13100_2017_97_MOESM1_ESM.zip › supplemental_S1A.pdf]
