# Supplementary material for: Globular domain structure and function of restriction-like-endonuclease LINEs: similarities to eukaryotic splicing factor Prp8
Source: Mob DNA. 2017 Nov 7;8:16. doi: 10.1186/s13100-017-0097-9 (PMC5678591; doi:10.1186/s13100-017-0097-9)
Supplement: Supplementary file 1 — Lys C mapping data. (ZIP 983 kb) [file 13100_2017_97_MOESM1_ESM.zip › supplemental_S1B.pdf]

Supplemental data S1B

Globular Domain Structure and Function of Restriction-Like-Endonuclease LINEs: Similarities to Eukaryotic Splicing Factor Prp8

M. Murshida Mahbub[1], Saiful M. Chowdhury[2]*, and Shawn M. Christensen[1]*

Figure: Internal peptide mapping of LysC resistant R2 fragments (LA to LH). Highlighted red texts are N-terminal end peptides for corresponding R2 fragments. Simple green texts represent peptide sequences obtained from LysC-trypsin digestion in a single run of ESI-MS/MS of the corresponding band. Green and italic texts indicate the peptide sequences obtained from LysC-trypsin digestion of the bands from second or following run of ESI-MS/MS. Underlined green texts represent LysC-GluC generated peptides. Blue texts are peptides common between two runs of ESI.

Number of ESI runs on the processed bands:

LA band: 2 ESI. 1 lysc-trp. 1 lysc-gluc. blue is common between 1 lysc-trp & 1 lysc-gluc

LB band: 3 ESI. 2 lysc-trp. 1 lysc-gluc. blue is common between 2 lysc-trp

LC band: 2 ESI. 2 lysc-trp. blue is common between 2 lysc-trp

LD band: 3 ESI. 2 lysc-trp. 1 lysc-gluc. blue is common between 2 lysc-trp

LE band: 2 ESI. 2 lysc-trp. blue is common between 2 lysc-trp

LG band: 3 ESI. 2 lysc-trp. 1 lysc-gluc. blue is common between 2 lysc-trp

LH band: 2 ESI. 1 lysc-trp. 1 lysc-gluc. blue is common between 1 lysc-trp & 1 lysc-gluc

Band LA

```
MKKSNKENRP EASGLPLESE RTGDNPTVRG
SAGADPVGQD APGWTCQFCE RTFSTNRGLG
VHKRRAHPVE TNTDAAPMMV KRRWHGEEID
LLARTEARLL AERGQCSGGD LFGALPGFGR
TLEAIKGQRR REPYRALVQA HLARFGSQPG
PSSGGCSAEP DFRRASGAEE AGEERCAEDA
AAYDPSAVGQ MSPDAARVLS ELLEGAGRRR
ACRAMRPKTA GRRNDLHDDR TASAHKTSRQ
KRRAEYARVQ ELYKKCRSRA AAEVIDGACG
GVGHSLEEME TYWRPILERV SDAPGPTPEA
LHALGRAEWH GGNRDYTQLW KPISVEEIKA
SRFDWRTSPG PDGIRSGQWR AVPVHLKAEM
FNAWMARGEI PEILRQCRTV FVPKVERPGG
PGEYRPISIA SIPLRHFHSI LARRLLACCP
PDARQRGFIC ADGTLENSAV LDAVLGDSRK
KLRECHVAVL DFAKAFDTVS HEALVELLRL
RGMPEQFCGY IAHLYDTAST TLAVNNEMSS
PVKVGRGVRQ GDPLSPILFN VVMDLILASL
PERVGYRLEM ELVSALAYAD DLVLLAGSKV
GMQESISAVD CVGRQMGLRL NCRKSAVLSM
IPDGHRKKHH YLTERTFNIG GKPLRQVSCV
ERWRYLGVDF EASGCVTLEH SISSALNNIS
RAPLKPQQRL EILRAHLIPR FQHGFVLGNI
SDDRLRMLDV QIRKAVGQWL RLPADVPKAY
YHAAVQDGGL AIPSVRATIP DLIVRRFGGL
DSSPWSVARA AAKSDKIRKK LRWAWKQLRR
FSRVDSTTQR PSVRLFWREH LHASVDGREL
RESTRTPTST KWIRERCAQI TGRDFVQFVH
THINALPSRI RGSRGRRGGG ESSLTCRAGC
KVRETTAHIL QQCHRTHGGR ILRHNKIVSF
VAKAMEENKW TVELEPRLRT SVGLRKPDII
ASRDGVGVIV DVQVVSGQRS LDELHREKRN
KYGNHGELVE LVAGRLGLPK AECVRATSCT
ISWRGVWSLT SYKELRSIIG LREPTLQIVP
ILALRGSHMN WTRFNQMTSV MGGGVGIEGR
HHHHHH
```

Band LB

```
MKKSNKENRP EASGLPLESE RTGDNPTVRG
SAGADPVGQD APGWTCQFCE RTFSTNRGLG
VHKRRAHPVE TNTDAAPMMV KRRWHGEEID
LLARTEARLL AERGQCSGGD LFGALPGFGR
TLEAIKGQRR REPYRALVQA HLARFGSQPG
PSSGGCSAEP DFRRASGAEE AGEERCAEDA
AAYDPSAVGQ MSPDAARVLS ELLEGAGRRR
ACRAMRPKTA GRRNDLHDDR TASAHKTSRQ
KRRAEYARVQ ELYKKCRSRA AAEVIDGACG
GVGHSLEEME TYWRPILERV SDAPGPTPEA
LHALGRAEWH GGNRDYTQLW KPISVEEIKA
SRFDWRTSPG PDGIRSGQWR AVPVHLKAEM
FNAWMARGEI PEILRQCRTV FVPKVERPGG
PGEYRPISIA SIPLRHFHSI LARRLLACCP
PDARQRGFIC ADGTLENSAV LDAVLGDSRK
KLRECHVAVL DFAKAFDTVS HEALVELLRL
RGMPEQFCGY IAHLYDTAST TLAVNNEMSS
PVKVGRGVRQ GDPLSPILFN VVMDLILASL
PERVGYRLEM ELVSALAYAD DLVLLAGSKV
GMQESISAVD CVGRQMGLRL NCRKSAVLSM
IPDGHRKKHH YLTERTFNIG GKPLRQVSCV
ERWRYLGVDF EASGCVTLEH SISSALNNIS
RAPLKPQQRL EILRAHLIPR FQHGFVLGNI
SDDRLRMLDV QIRKAVGQWL RLPADVPKAY
YHAAVQDGGL AIPSVRATIP DLIVRRFGGL
DSSPWSVARA AAKSDKIRKK LRWAWKQLRR
FSRVDSTTQR PSVRLFWREH LHASVDGREL
RESTRTPTST KWIRERCAQI TGRDFVQFVH
THINALPSRI RGSRGRRGGG ESSLTCRAGC
KVRETTAHIL QQCHRTHGGR ILRHNKIVSF
VAKAMEENKW TVELEPRLRT SVGLRKPDII
ASRDGVGVIV DVQVVSGQRS LDELHREKRN
KYGNHGELVE LVAGRLGLPK AECVRATSCT
ISWRGVWSLT SYKELRSIIG LREPTLQIVP
ILALRGSHMN WTRFNQMTSV MGGGVGIEGR
HHHHHH
```

Band LC

```
MKKSNKENRP EASGLPLESE RTGDNPTVRG
SAGADPVGQD APGWTCQFCE RTFSTNRGLG
VHKRRAHPVE TNTDAAPMMV KRRWHGEEID
LLARTEARLL AERGQCSGGD LFGALPGFGR
TLEAIKGQRR REPYRALVQA HLARFGSQPG
PSSGGCSAEP DFRRASGAEE AGEERCAEDA
AAYDPSAVGQ MSPDAARVLS ELLEGAGRRR
ACRAMRPKTA GRRNDLHDDR TASAHKTSRQ
KRRAEYARVQ ELYKKCRSRA AAEVIDGACG
GVGHSLEEME TYWRPILERV SDAPGPTPEA
LHALGRAEWH GGNRDYTQLW KPISVEEIKA
SRFDWRTSPG PDGIRSGQWR AVPVHLKAEM
FNAWMARGEI PEILRQCRTV FVPKVERPGG
PGEYRPISIA SIPLRHFHSI LARRLLACCP
PDARQRGFIC ADGTLENSAV LDAVLGDSRK
KLRECHVAVL DFAKAFDTVS HEALVELLRL
RGMPEQFCGY IAHLYDTAST TLAVNNEMSS
PVKVGRGVRQ GDPLSPILFN VVMDLILASL
PERVGYRLEM ELVSALAYAD DLVLLAGSKV
GMQESISAVD CVGRQMGLRL NCRKSAVLSM
IPDGHRKKHH YLTERTFNIG GKPLRQVSCV
ERWRYLGVDF EASGCVTLEH SISSALNNIS
RAPLKPQQRL EILRAHLIPR FQHGFVLGNI
SDDRLRMLDV QIRKAVGQWL RLPADVPKAY
YHAAVQDGGL AIPSVRATIP DLIVRRFGGL
DSSPWSVARA AAKSDKIRKK LRWAWKQLRR
FSRVDSTTQR PSVRLFWREH LHASVDGREL
RESTRTPTST KWIRERCAQI TGRDFVQFVH
THINALPSRI RGSRGRRGGG ESSLTCRAGC
KVRETTAHIL QQCHRTHGGR ILRHNKIVSF
VAKAMEENKW TVELEPRLRT SVGLRKPDII
ASRDGVGVIV DVQVVSGQRS LDELHREKRN
KYGNHGELVE LVAGRLGLPK AECVRATSCT
ISWRGVWSLT SYKELRSIIG LREPTLQIVP
ILALRGSHMN WTRFNQMTSV MGGGVGIEGR
HHHHHH
```

Band LD

MKKSNKENRP EASGLPLESE RTGDNPTVRG
SAGADPVGQD APGWTCQFCE RTFSTNRGLG
VHKRRAHPVE TNTDAAPMMV KRRWHGEEID
LLARTEARLL AERGQCSGGD LFGALPGFGR
TLEAIKGQRR REPYRALVQA HLARFGSQPG
PSSGGCSAEP DFRRASGAEE AGEERCAEDA
AAYDPSAVGQ MSPDAARVLS ELLEGAGRRR
ACRAMRPKTA GRRNDLHDDR TASAHKTSRQ
KRRAEYARVQ ELYKKCRSRA AAEVIDGACG
GVGHSLEEME TYWRPILERV SDAPGPTPEA
LHALGRAEWH GGNRDYTQLW KPISVEEIKA
SRFDWRTSPG PDGIRSGQWR AVPVHLKAEM
FNAWMARGEI PEILRQCRTV FVPKVERPGG
PGEYRPISIA SIPLRHFHSI LARRLLACCP
PDARQRGFIC ADGTLENSAV LDAVLGDSRK
KLRECHVAVL DFAKAFDTVS HEALVELLRL
RGMPEQFCGY IAHLYDTAST TLAVNNEMSS
PVKVGRGVRQ GDPLSPILFN VVMDLILASL
PERVGYRLEM ELVSALAYAD DLVLLAGSKV
GMQESISAVD CVGRQMGLRL NCRKSAVLSM
IPDGHRKKHH YLTERTFNIG GKPLRQVSCV
ERWRYLGVDF EASGCVTLEH SISSALNNIS
RAPLKPQQRL EILRAHLIPR FQHGFVLGNI
SDDRLRMLDV QIRKAVGQWL RLPADVPKAY
YHAAVQDGGL AIPSVRATIP DLIVRRFGGL
DSSPWSVARA AAKSDKIRKK LRWAWKQLRR
FSRVDSTTQR PSVRLFWREH LHASVDGREL
RESTRTPTST KWIRERCAQI TGRDFVQFVH
THINALPSRI RGSRGRRGGG ESSLTCRAGC
KVRETTAHIL QQCHRTHGGR ILRHNKIVSF
VAKAMEENKW TVELEPRLRT SVGLRKPDII
ASRDGVGVIV DVQVVSGQRS LDELHREKRN
KYGNHGELVE LVAGRLGLPK AECVRATSCT
ISWRGVWSLT SYKELRSIIG LREPTLQIVP
ILALRGSHMN WTRFNQMTSV MGGGVGIEGR
HHHHHH

Band LE

MKKSNKENRP EASGLPLESE RTGDNPTVRG
SAGADPVGQD APGWTCQFCE RTFSTNRGLG
VHKRRAHPVE TNTDAAPMMV KRRWHGEEID
LLARTEARLL AERGQCSGGD LFGALPGFGR
TLEAIKGQRR REPYRALVQA HLARFGSQPG
PSSGGCSAEP DFRRASGAEE AGEERCAEDA
AAYDPSAVGQ MSPDAARVLS ELLEGAGRRR
ACRAMRPKTA GRRNDLHDDR TASAHKTSRQ
KRRAEYARVQ ELYKKCRSRA AAEVIDGACG
GVGHSLEEME TYWRPILERV SDAPGPTPEA
LHALGRAEWH GGNRDYTQLW KPISVEEIKA
SRFDWRTSPG PDGIRSGQWR AVPVHLKAEM
FNAWMARGEI PEILRQCRTV FVPKVERPGG
PGEYRPISIA SIPLRHFHSI LARRLLACCP
PDARQRGFIC ADGTLENSAV LDAVLGDSRK
KLRECHVAVL DFAKAFDTVS HEALVELLRL
RGMPEQFCGY IAHLYDTAST TLAVNNEMSS
PVKVGRGVRQ GDPLSPILFN VVMDLILASL
PERVGYRLEM ELVSALAYAD DLVLLAGSKV
GMQESISAVD CVGRQMGLRL NCRKSAVLSM
IPDGHRKKHH YLTERTFNIG GKPLRQVSCV
ERWRYLGVDF EASGCVTLEH SISSALNNIS
RAPLKPQQRL EILRAHLIPR FQHGFVLGNI
SDDRLRMLDV QIRKAVGQWL RLPADVPKAY
YHAAVQDGGL AIPSVRATIP DLIVRRFGGL
DSSPWSVARA AAKSDKIRKK LRWAWKQLRR
FSRVDSTTQR PSVRLFWREH LHASVDGREL
RESTRTPTST KWIRERCAQI TGRDFVQFVH
THINALPSRI RGSRGRRGGG ESSLTCRAGC
KVRETTAHIL QQCHRTHGGR ILRHNKIVSF
VAKAMEENKW TVELEPRLRT SVGLRKPDII
ASRDGVGVIV DVQVVSGQRS LDELHREKRN
KYGNHGELVE LVAGRLGLPK AECVRATSCT
ISWRGVWSLT SYKELRSIIG LREPTLQIVP
ILALRGSHMN WTRFNQMTSV MGGGVGIEGR
HHHHHH

Band LF

MKKSNKENRP EASGLPLESE RTGDNPTVRG
SAGADPVGQD APGWTCQFCE RTFSTNRGLG
VHKRRAHPVE TNTDAAPMMV KRRWHGEEID
LLARTEARLL AERGQCSGGD LFGALPGFGR
TLEAIKGQRR REPYRALVQA HLARFGSQPG
PSSGGCSAEP DFRRASGAEE AGEERCAEDA
AAYDPSAVGQ MSPDAARVLS ELLEGAGRRR
ACRAMRPKTA GRRNDLHDDR TASAHKTSRQ
KRRAEYARVQ ELYKKCRSRA AAEVIDGACG
GVGHSLEEME TYWRPILERV SDAPGPTPEA
LHALGRAEWH GGNRDYTQLW KPISVEEIKA
SRFDWRTSPG PDGIRSGQWR AVPVHLKAEM
FNAWMARGEI PEILRQCRTV FVPKVERPGG
PGEYRPISIA SIPLRHFHSI LARRLLACCP
PDARQRGFIC ADGTLENSAV LDAVLGDSRK
KLRECHVAVL DFAKAFDTVS HEALVELLRL
RGMPEQFCGY IAHLYDTAST TLAVNNEMSS
PVKVGRGVRQ GDPLSPILFN VVMDLILASL
PERVGYRLEM ELVSALAYAD DLVLLAGSKV
GMQESISAVD CVGRQMGLRL NCRKSAVLSM
IPDGHRKKHH YLTERTFNIG GKPLRQVSCV
ERWRYLGVDF EASGCVTLEH SISSALNNIS
RAPLKPQQRL EILRAHLIPR FQHGFVLGNI
SDDRLRMLDV QIRKAVGQWL RLPADVPKAY
YHAAVQDGGL AIPSVRATIP DLIVRRFGGL
DSSPWSVARA AAKSDKIRKK LRWAWKQLRR
FSRVDSTTQR PSVRLFWREH LHASVDGREL
RESTRTPTST KWIRERCAQI TGRDFVQFVH
THINALPSRI RGSRGRRGGG ESSLTCRAGC
KVRETTAHIL QQCHRTHGGR ILRHNKIVSF
VAKAMEENKW TVELEPRLRT SVGLRKPDII
ASRDGVGVIV DVQVVSGQRS LDELHREKRN
KYGNHGELVE LVAGRLGLPK AECVRATSCT
ISWRGVWSLT SYKELRSIIG LREPTLQIVP
ILALRGSHMN WTRFNQMTSV MGGGVGIEGR
HHHHHH

Band LG

MKKSNKENRP EASGLPLESE RTGDNPTVRG
SAGADPVGQD APGWTCQFCE RTFSTNRGLG
VHKRRAHPVE TNTDAAPMMV KRRWHGEEID
LLARTEARLL AERGQCSGGD LFGALPGFGR
TLEAIKGQRR REPYRALVQA HLARFGSQPG
PSSGGCSAEP DFRRASGAEE AGEERCAEDA
AAYDPSAVGQ MSPDAARVLS ELLEGAGRRR
ACRAMRPKTA GRRNDLHDDR TASAHKTSRQ
KRRAEYARVQ ELYKKCRSRA AAEVIDGACG
GVGHSLEEME TYWRPILERV SDAPGPTPEA
LHALGRAEWH GGNRDYTQLW KPISVEEIKA
SRFDWRTSPG PDGIRSGQWR AVPVHLKAEM
FNAWMARGEI PEILRQCRTV FVPKVERPGG
PGEYRPISIA SIPLRHFHSI LARRLLACCP
PDARQRGFIC ADGTLENSAV LDAVLGDSRK
KLRECHVAVL DFAKAFDTVS HEALVELLRL
RGMPEQFCGY IAHLYDTAST TLAVNNEMSS
PVKVGRGVRQ GDPLSPILFN VVMDLILASL
PERVGYRLEM ELVSALAYAD DLVLLAGSKV
GMQESISAVD CVGRQMGLRL NCRKSAVLSM
IPDGHRKKHH YLTERTFNIG GKPLRQVSCV
ERWRYLGVDF EASGCVTLEH SISSALNNIS
RAPLKPQQRL EILRAHLIPR FQHGFVLGNI
SDDRLRMLDV QIRKAVGQWL RLPADVPKAY
YHAAVQDGGL AIPSVRATIP DLIVRRFGGL
DSSPWSVARA AAKSDKIRKK LRWAWKQLRR
FSRVDSTTQR PSVRLFWREH LHASVDGREL
RESTRTPTST KWIRERCAQI TGRDFVQFVH
THINALPSRI RGSRGRRGGG ESSLTCRAGC
KVRETTAHIL QQCHRTHGGR ILRHNKIVSF
VAKAMEENKW TVELEPRLRT SVGLRKPDII
ASRDGVGVIV DVQVVSGQRS LDELHREKRN
KYGNHGELVE LVAGRLGLPK AECVRATSCT
ISWRGVWSLT SYKELRSIIG LREPTLQIVP
ILALRGSHMN WTRFNQMTSV MGGGVGIEGR
HHHHHH

Band LH

MKKSNKENRP EASGLPLESE RTGDNPTVRG
SAGADPVGQD APGWTCQFCE RTFSTNRGLG
VHKRRAHPVE TNTDAAPMMV KRRWHGEEID
LLARTEARLL AERGQCSGGD LFGALPGFGR
TLEAIKGQRR REPYRALVQA HLARFGSQPG
PSSGGCSAEP DFRRASGAEE AGEERCAEDA
AAYDPSAVGQ MSPDAARVLS ELLEGAGRRR
ACRAMRPKTA GRRNDLHDDR TASAHKTSRQ
KRRAEYARVQ ELYKKCRSRA AAEVIDGACG
GVGHSLEEME TYWRPILERV SDAPGPTPEA
LHALGRAEWH GGNRDYTQLW KPISVEEIKA
SRFDWRTSPG PDGIRSGQWR AVPVHLKAEM
FNAWMARGEI PEILRQCRTV FVPKVERPGG
PGEYRPISIA SIPLRHFHSI LARRLLACCP
PDARQRGFIC ADGTLENSAV LDAVLGDSRK
KLRECHVAVL DFAKAFDTVS HEALVELLRL
RGMPEQFCGY IAHLYDTAST TLAVNNEMSS
PVKVGRGVRQ GDPLSPILFN VVMDLILASL
PERVGYRLEM ELVSALAYAD DLVLLAGSKV
GMQESISAVD CVGRQMGLRL NCRKSAVLSM
IPDGHRKKHH YLTERTFNIG GKPLRQVSCV
ERWRYLGVDF EASGCVTLEH SISSALNNIS
RAPLKPQQRL EILRAHLIPR FQHGFVLGNI
SDDRLRMLDV QIRKAVGQWL RLPADVPKAY
YHAAVQDGGL AIPSVRATIP DLIVRRFGGL
DSSPWSVARA AAKSDKIRKK LRWAWKQLRR
FSRVDSTTQR PSVRLFWREH LHASVDGREL
RESTRTPTST KWIRERCAQI TGRDFVQFVH
THINALPSRI RGSRGRRGGG ESSLTCRAGC
KVRETTAHIL QQCHRTHGGR ILRHNKIVSF
VAKAMEENKW TVELEPRLRT SVGLRKPDII
ASRDGVGVIV DVQVVSGQRS LDELHREKRN
KYGNHGELVE LVAGRLGLPK AECVRATSCT
ISWRGVWSLT SYKELRSIIG LREPTLQIVP
ILALRGSHMN WTRFNQMTSV MGGGVGIEGR
HHHHHH

Band LI

MKKSNKENRP EASGLPLESE RTGDNPTVRG
SAGADPVGQD APGWTCQFCE RTFSTNRGLG
VHKRRAHPVE TNTDAAPMMV KRRWHGEEID
LLARTEARLL AERGQCSGGD LFGALPGFGR
TLEAIKGQRR REPYRALVQA HLARFGSQPG
PSSGGCSAEP DFRRASGAEE AGEERCAEDA
AAYDPSAVGQ MSPDAARVLS ELLEGAGRRR
ACRAMRPKTA GRRNDLHDDR TASAHKTSRQ
KRRAEYARVQ ELYKKCRSRA AAEVIDGACG
GVGHSLEEME TYWRPILERV SDAPGPTPEA
LHALGRAEWH GGNRDYTQLW KPISVEEIKA
SRFDWRTSPG PDGIRSGQWR AVPVHLKAEM
FNAWMARGEI PEILRQCRTV FVPKVERPGG
PGEYRPISIA SIPLRHFHSI LARRLLACCP
PDARQRGFIC ADGTLENSAV LDAVLGDSRK
KLRECHVAVL DFAKAFDTVS HEALVELLRL
RGMPEQFCGY IAHLYDTAST TLAVNNEMSS
PVKVGRGVRQ GDPLSPILFN VVMDLILASL
PERVGYRLEM ELVSALAYAD DLVLLAGSKV
GMQESISAVD CVGRQMGLRL NCRKSAVLSM
IPDGHRKKHH YLTERTFNIG GKPLRQVSCV
ERWRYLGVDF EASGCVTLEH SISSALNNIS
RAPLKPQQRL EILRAHLIPR FQHGFVLGNI
SDDRLRMLDV QIRKAVGQWL RLPADVPKAY
YHAAVQDGGL AIPSVRATIP DLIVRRFGGL
DSSPWSVARA AAKSDKIRKK LRWAWKQLRR
FSRVDSTTQR PSVRLFWREH LHASVDGREL
RESTRTPTST KWIRERCAQI TGRDFVQFVH
THINALPSRI RGSRGRRGGG ESSLTCRAGC
KVRETTAHIL QQCHRTHGGR ILRHNKIVSF
VAKAMEENKW TVELEPRLRT SVGLRKPDII
ASRDGVGVIV DVQVVSGQRS LDELHREKRN
KYGNHGELVE LVAGRLGLPK AECVRATSCT
ISWRGVWSLT SYKELRSIIG LREPTLQIVP
ILALRGSHMN WTRFNQMTSV MGGGVGIEGR
HHHHHH
