# Supplementary material for: Globular domain structure and function of restriction-like-endonuclease LINEs: similarities to eukaryotic splicing factor Prp8
Source: Mob DNA. 2017 Nov 7;8:16. doi: 10.1186/s13100-017-0097-9 (PMC5678591; doi:10.1186/s13100-017-0097-9)
Supplement: Supplementary file 2 — R2Bm sequence and domain boundaries. (DOCX 28 kb) [file 13100_2017_97_MOESM2_ESM.docx]

# Delta N R2Bm protein sequence

# MKKSNKENRPEASGLPLESERTGDNPTVRGSAGADPVGQDAPGWTCQFCERTFSTNRGLGVHKRRAHPVETNTDAAPMMVKRRWHGEEIDLLARTEARLLAERGQCSGGDLFGALPGFGRTLEAIKGQRRREPYRALVQAHLARFGSQPGPSSGGCSAEPDFRRASGAEEAGEERCAEDAAAYDPSAVGQMSPDAARVLSELLEGAGRRRACRAMRPKTAGRRNDLHDDRTASAHKTSRQKRRAEYARVQELYKKCRSRAAAEVIDGACGGVGHSLEEMETYWRPILERVSDAPGPTPEALHALGRAEWHGGNRDYTQLWKPISVEEIKASRFDWRTSPGPDGIRSGQWRAVPVHLKAEMFNAWMARGEIPEILRQCRTVFVPKVERPGGPGEYRPISIASIPLRHFHSILARRLLACCPPDARQRGFICADGTLENSAVLDAVLGDSRKKLRECHVAVLDFAKAFDTVSHEALVELLRLRGMPEQFCGYIAHLYDTASTTLAVNNEMSSPVKVGRGVRQGDPLSPILFNVVMDLILASLPERVGYRLEMELVSALAYADDLVLLAGSKVGMQESISAVDCVGRQMGLRLNCRKSAVLSMIPDGHRKKHHYLTERTFNIGGKPLRQVSCVERWRYLGVDFEASGCVTLEHSISSALNNISRAPLKPQQRLEILRAHLIPRFQHGFVLGNISDDRLRMLDVQIRKAVGQWLRLPADVPKAYYHAAVQDGGLAIPSVRATIPDLIVRRFGGLDSSPWSVARAAAKSDKIRKKLRWAWKQLRRFSRVDSTTQRPSVRLFWREHLHASVDGRELRESTRTPTSTKWIRERCAQITGRDFVQFVHTHINALPSRIRGSRGRRGGGESSLTCRAGCKVRETTAHILQQCHRTHGGRILRHNKIVSFVAKAMEENKWTVELEPRLRTSVGLRKPDIIASRDGVGVIVDVQVVSGQRSLDELHREKRNKYGNHGELVELVAGRLGLPKAECVRATSCTISWRGVWSLTSYKELRSIIGLREPTLQIVPILALRGSHMNWTRFNQMTSVMGGGVGIEGRHHHHHH

Blue = N-terminal zinc finger and myb DNA binding domain

Green = Reverse transcriptase

Brick red = Linker region

Orange red = Restriction like endonuclease

| Motif | Position in delta N R2Bm protein sequence (tentative) |
| --- | --- |
| Index finger | 276-288 |
| Palm traversing helix (pth) | 298-314 |
| RT0 (Pinky finger) | 325-367 |
| G-loop | 337-350 |
| RT1 | 373-399 |
| RT2 | 402-418 |
| RT2a | 419-445 |
| RT3 | 446-468 |
| RT3a | 471-490 |
| RT4 | 499-539 |
| RT5 | 555-566 |
| RT6 | 569-596 |
| RT6a | 597-616 |
| RT7 | 617-644 |
| Thumb | 645-730 |
| Linker | 730-892 |
| HINALP | 829-847 |
| CCHC zinc knuckle | 866-884 |

Figure: Resemblance of a hand like structure of the R2 reverse transcriptase model. Different subdomains of reverse transcriptase are positioned tentatively on corresponding fingers or palm area.
