# Supplementary material for: Globular domain structure and function of restriction-like-endonuclease LINEs: similarities to eukaryotic splicing factor Prp8
Source: Mob DNA. 2017 Nov 7;8:16. doi: 10.1186/s13100-017-0097-9 (PMC5678591; doi:10.1186/s13100-017-0097-9)

Supplementary data S2

Globular Domain Structure and Function of Restriction-Like-Endonuclease LINEs: Similarities to Eukaryotic Splicing Factor Prp8

M. Murshida Mahbub[1], Saiful M. Chowdhury[2]*, and Shawn M. Christensen1*

Figure: MS/MS spectra for N-term end peptides of GluC bands. The MS/MS spectra of peptide ions were matched to corresponding hypothetical sequences. Individual matched y-ions are indicated in blue and b-ions are indicated in red. The x-axis units are m/z and the y axes represent relative abundance normalized to the most intensive fragment ion.

GA (i) N-term peptide: L*YK*K*C!R; z= +2; Xcorr =2.69; Theo. [M+H]1+ = 993.4868; [M+2H]2+ = 497.45 ; #PSMs = 18; * = acetyl; ! = carbamidomethyl

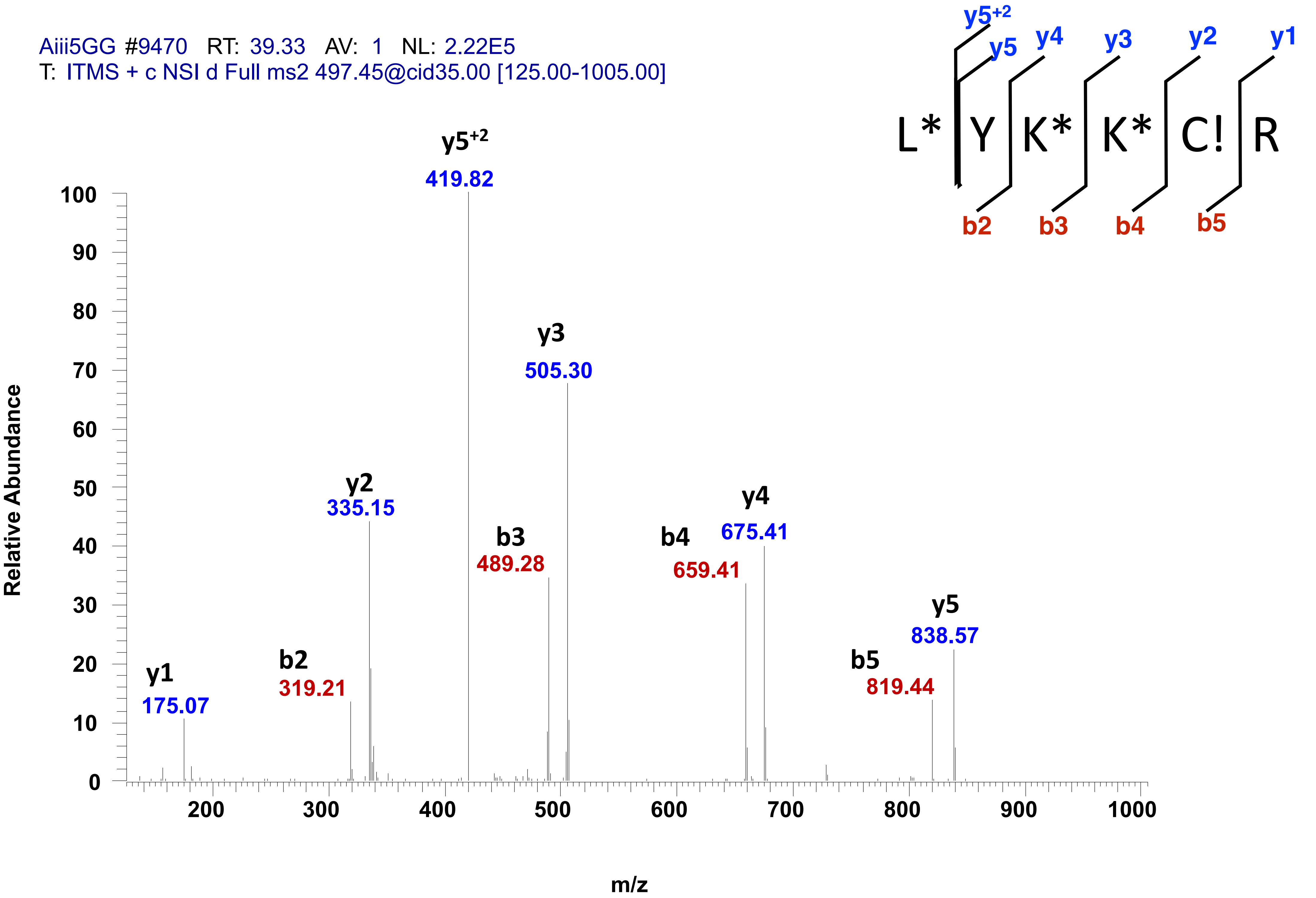

GA(ii) N-term peptide: M*ETYWRPILER; z= +2; Xcorr =1.5; Theo. [M+H]1+ = 1535.7569; [M+2H]2+ = 768.30; #PSMs = 3; * = acetyl

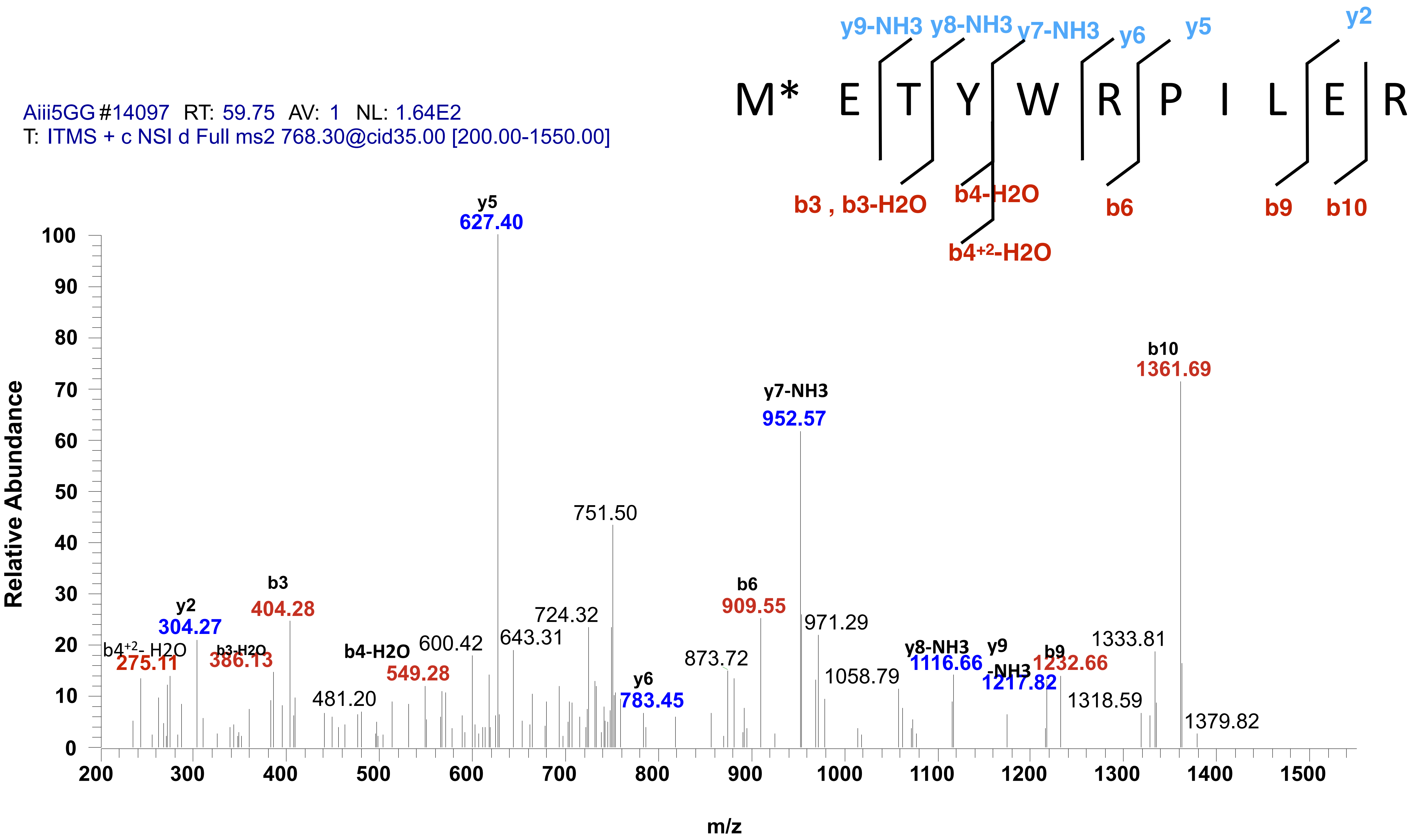

GA (iii) N-term peptide: T*YWRPILER; z= +2; Xcorr =2.12; Theo. [M+H]1+ = 1275.6738; [M+2H]2+ = 638.52; #PSMs = 4; * = acetyl

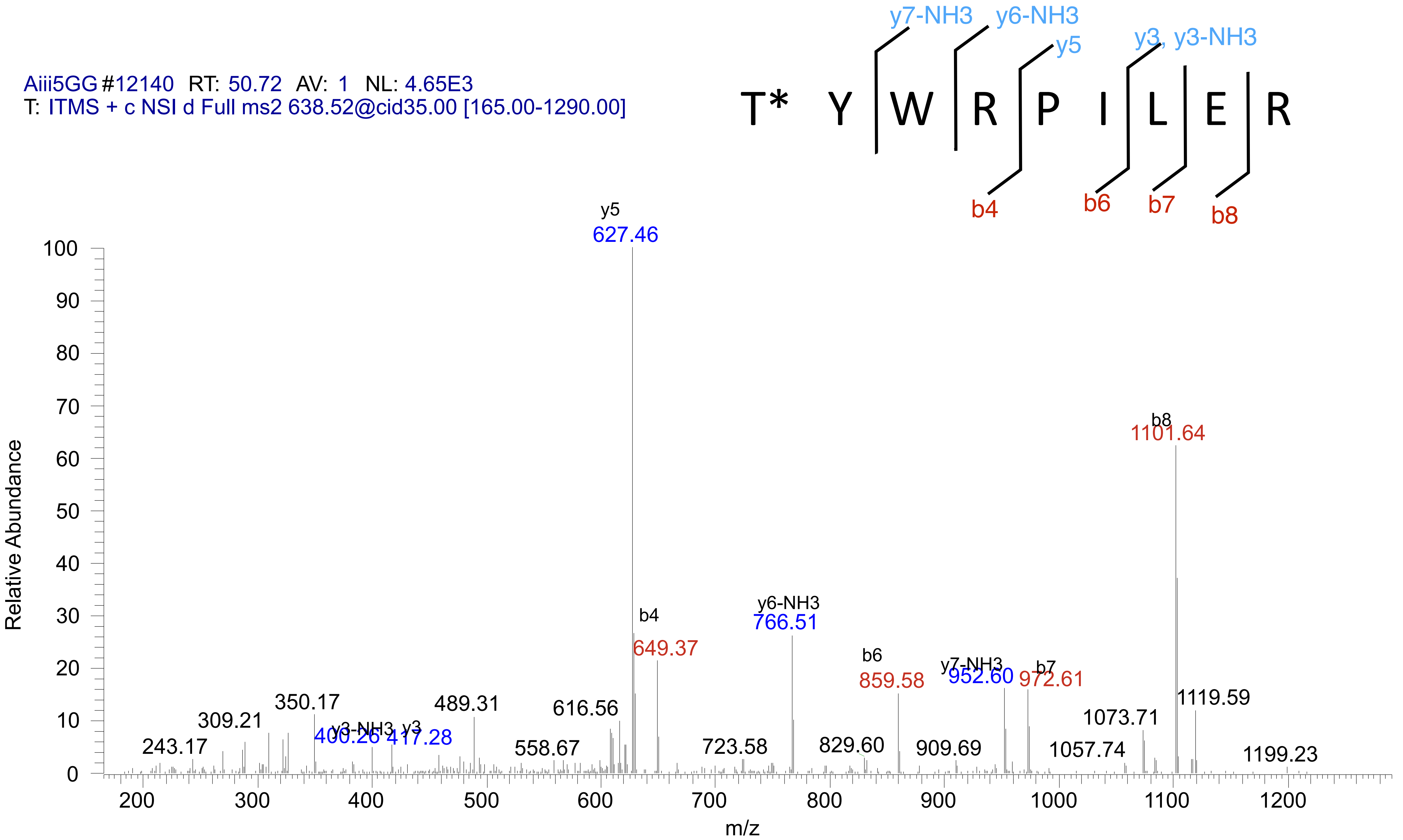

GA(iv) N-term peptide: A*LHALGR; z= +2; XCorr =2.41; Theo. [M+H]1+ = 779.4416; [M+2H]2+ = 390.41 ; #PSMs = 6; * = acetyl

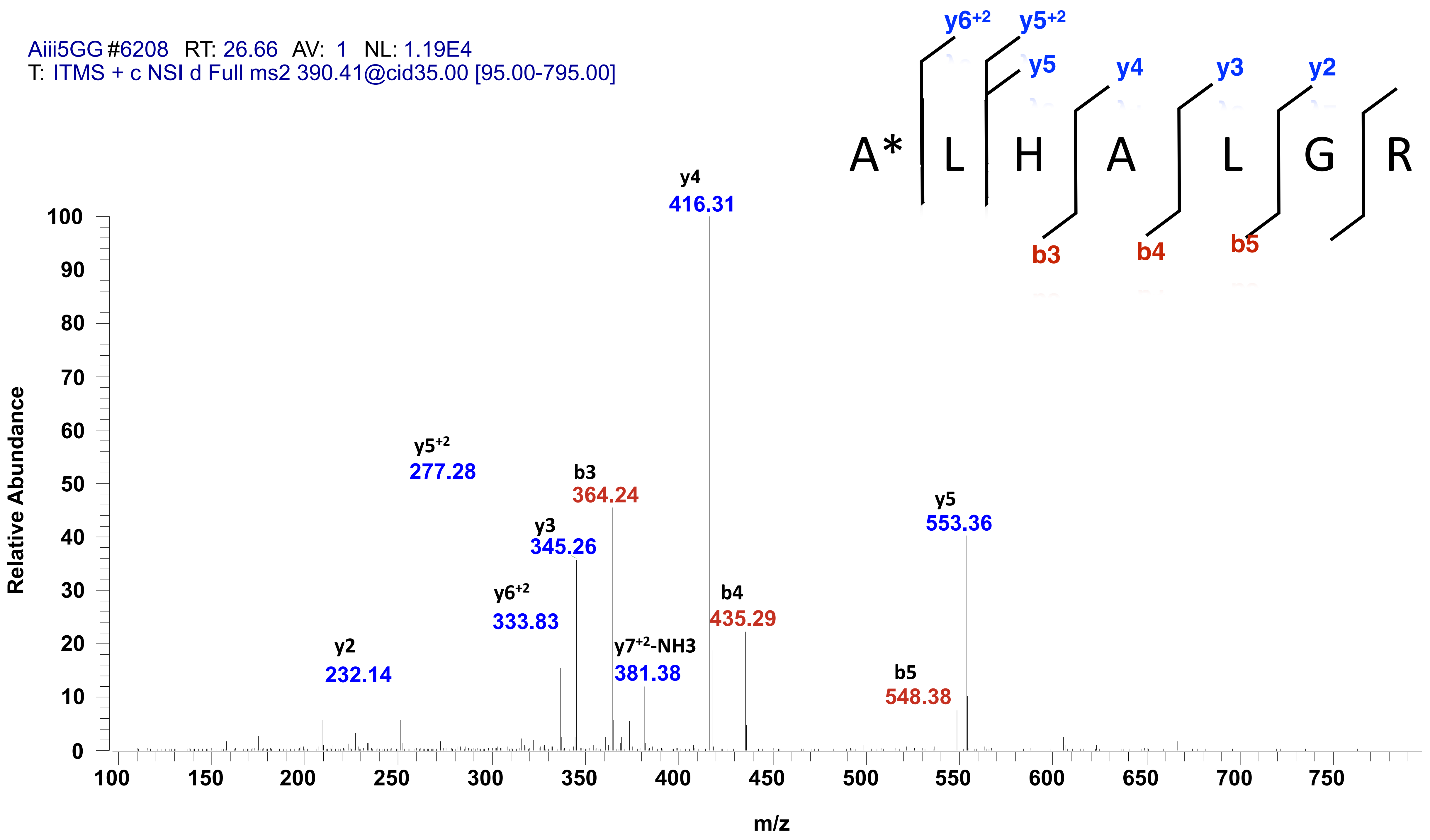

GB N-term peptide: N*SAVLDAVLGDSR; z= +2; XCorr =3.08; Theo. [M+H]1+ = 1358.6804; [M+2H]2+ = 679.75 ; #PSMs = 3; * = acetyl

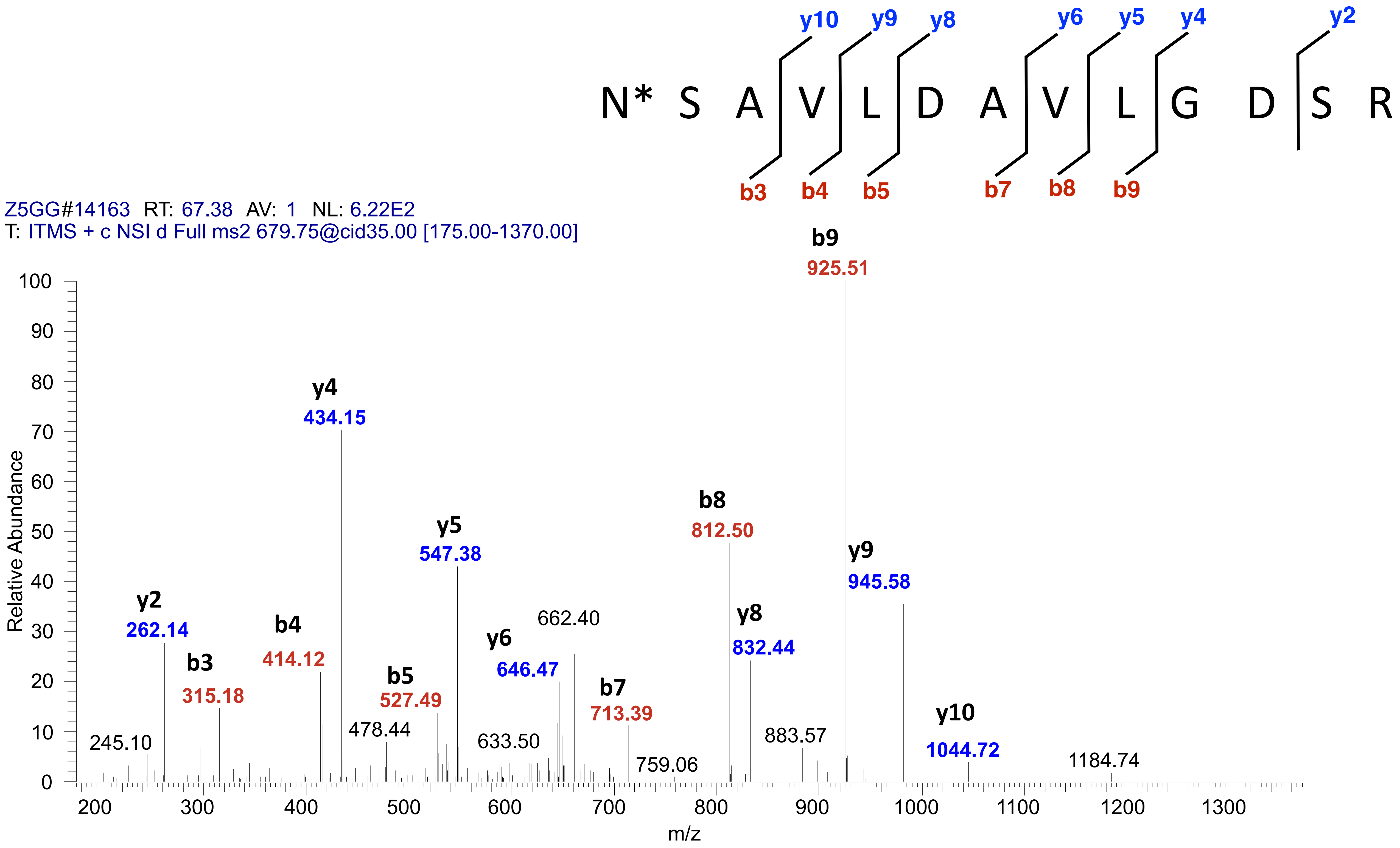

GC N-term peptide: M*SSPVK*VGR; z= +2; XCorr =2.90; Theo. [M+H]1+ = 1044.5294; [M+2H]2+ = 522.51 ; #PSMs = 2; * = acetyl

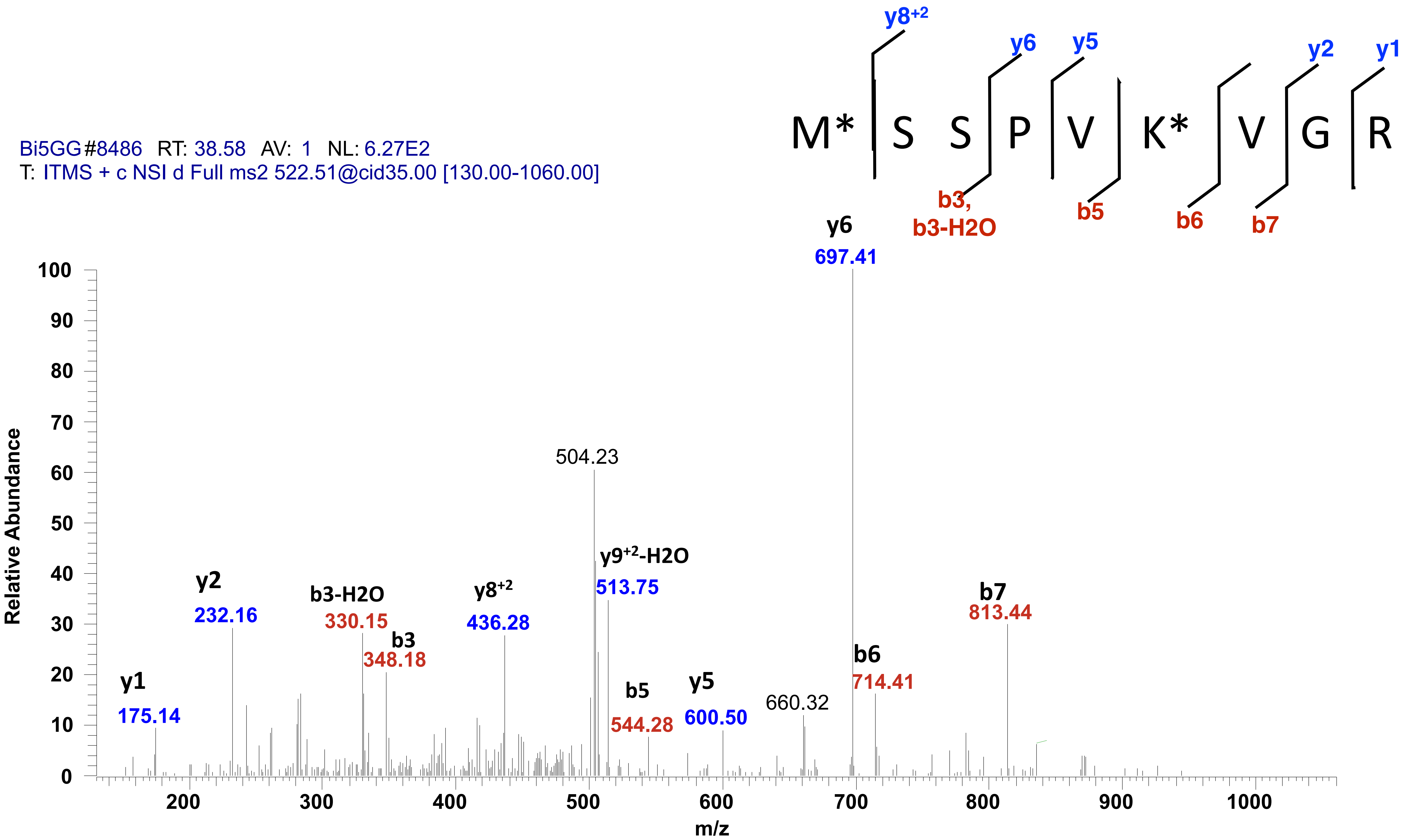

GD N-term peptide: S*ISAVDC!VGR; z= +2; XCorr = 3.67; Theo. [M+H]1+ = 1105.5200; [M+2H]2+ = 553.40; #PSMs = 31; * = acetyl; ! = carbamidomethyl

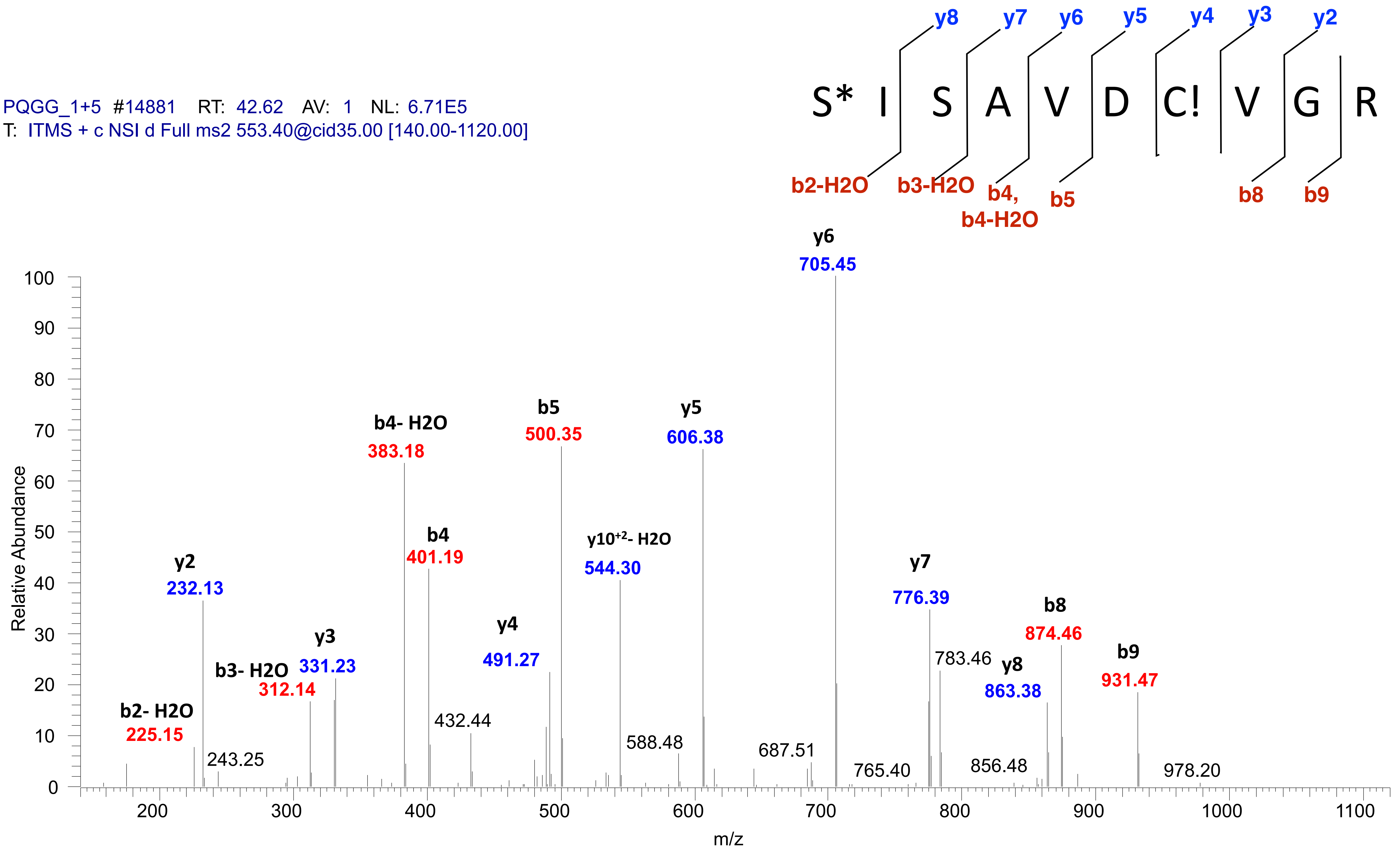

GE N-term peptide: R*TFNIGGK*PLR; z= +2; XCorr =2.65; Theo. [M+H]1+ = 1342.7378; [M+2H]2+ = 671.68 ; #PSMs = 4; * = acetyl

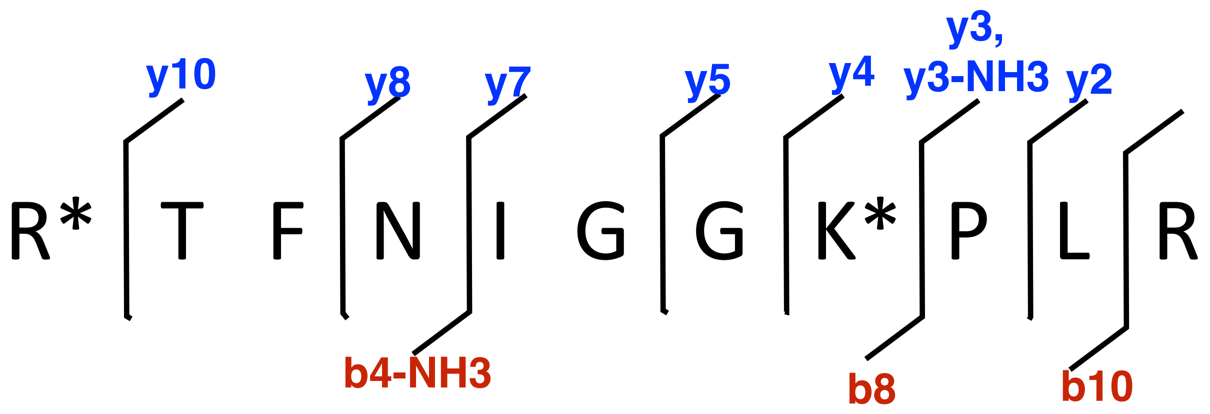

C9GG #8984 RT: 40.66 AV: 1 NL: 3.62E2
T: ITMS + c NSI d Full ms2 671.68@cid35.00 [170.00-1355.00]

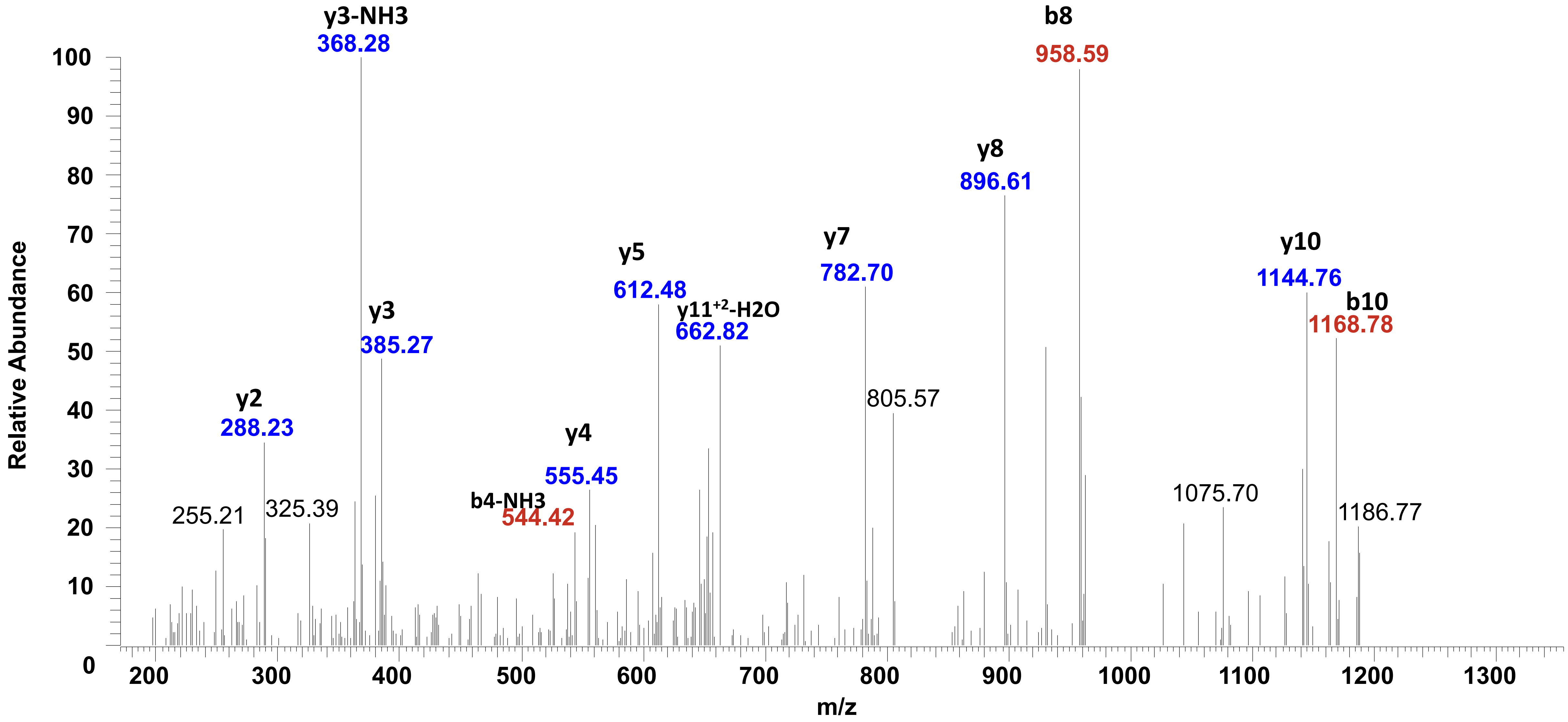

GF N-term peptide: H*SISSALNNISR; z= +2; XCorr =4.21; Theo. [M+H]1+ = 1340.6811; [M+2H]2+ = 670.98 ; #PSMs = 6; * = acetyl

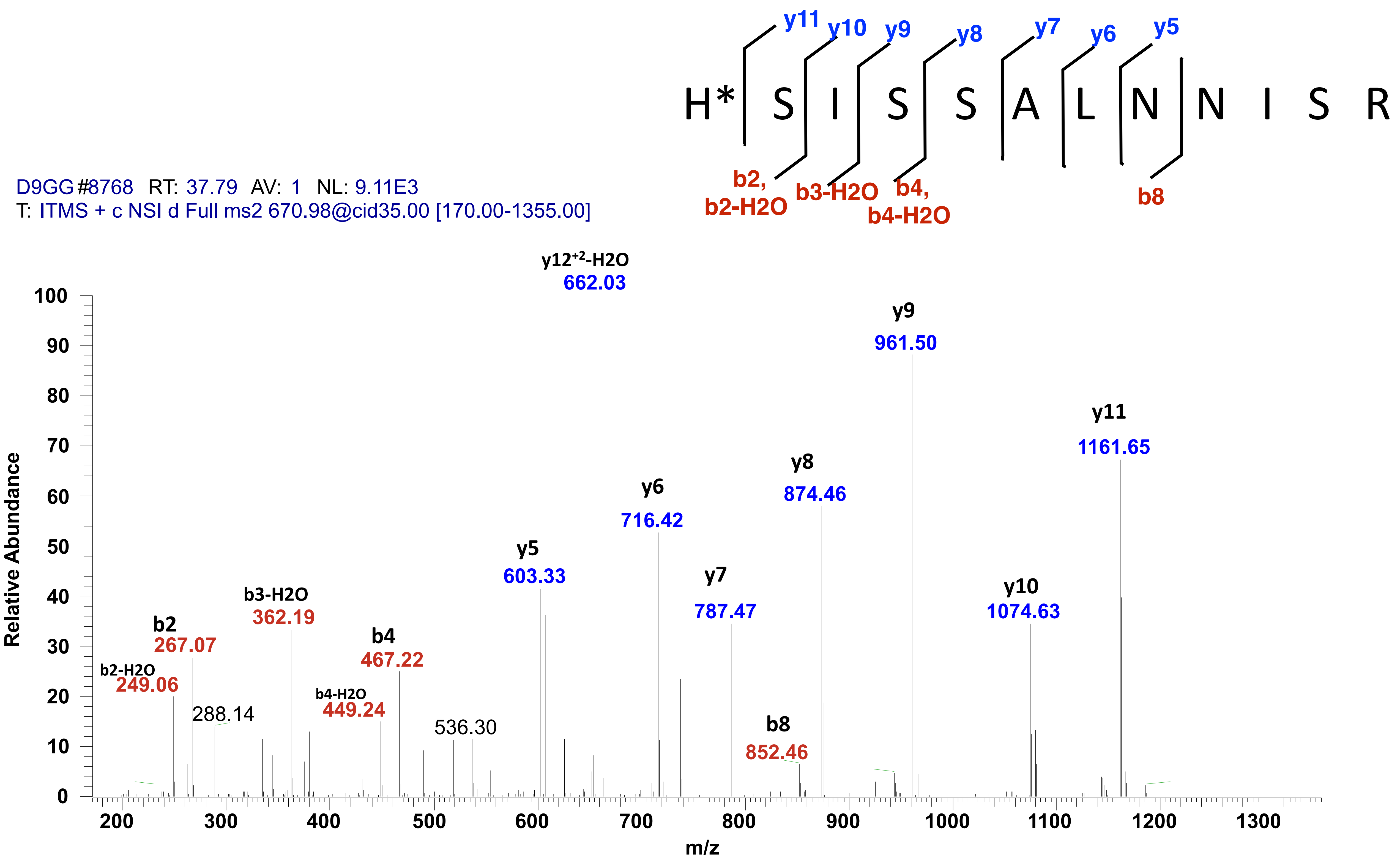

GH(i) N-term peptide: N*RPEASGLPLESER; z= +2; XCorr =3.68; Theo. [M+H]1+ = 1596.7870; [M+2H]2+ = 799.12 ; #PSMs = 2; * = acetyl

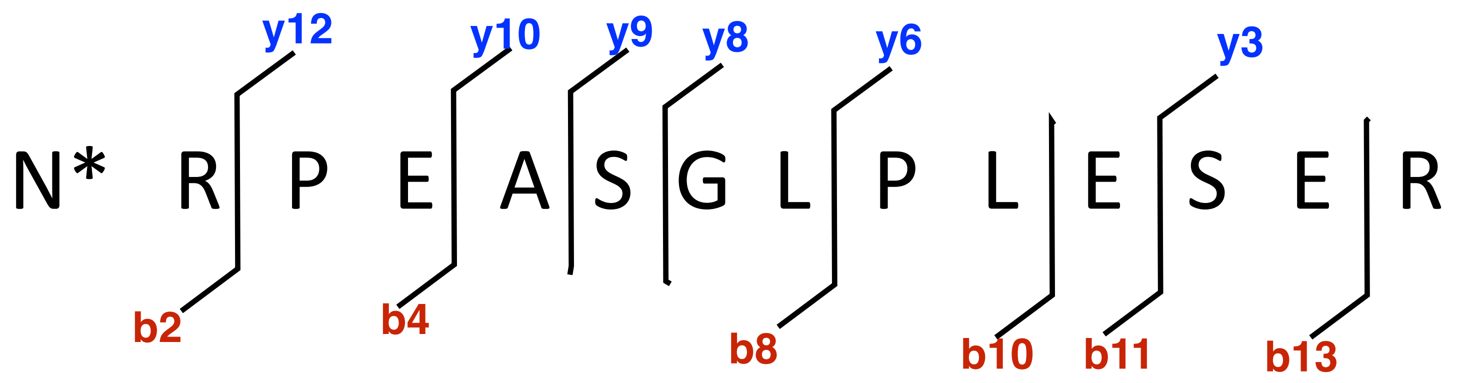

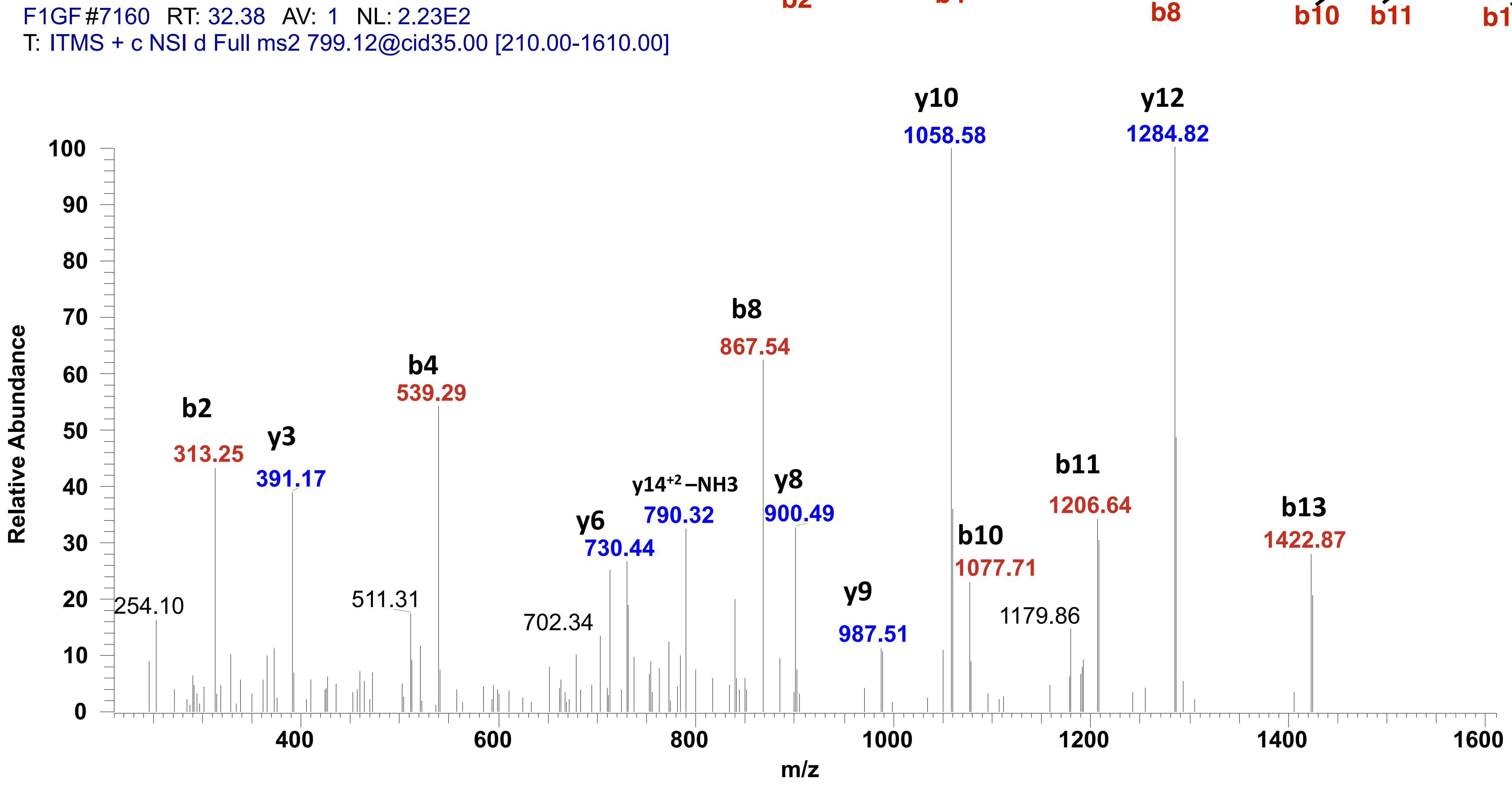

GH (ii) N-term peptide: A*SGLPLESER; z= +2; XCorr =2.47; Theo. [M+H]1+ = 1100.5476; [M+2H]2+ = 551.09 ; #PSMs = 13; * = acetyl

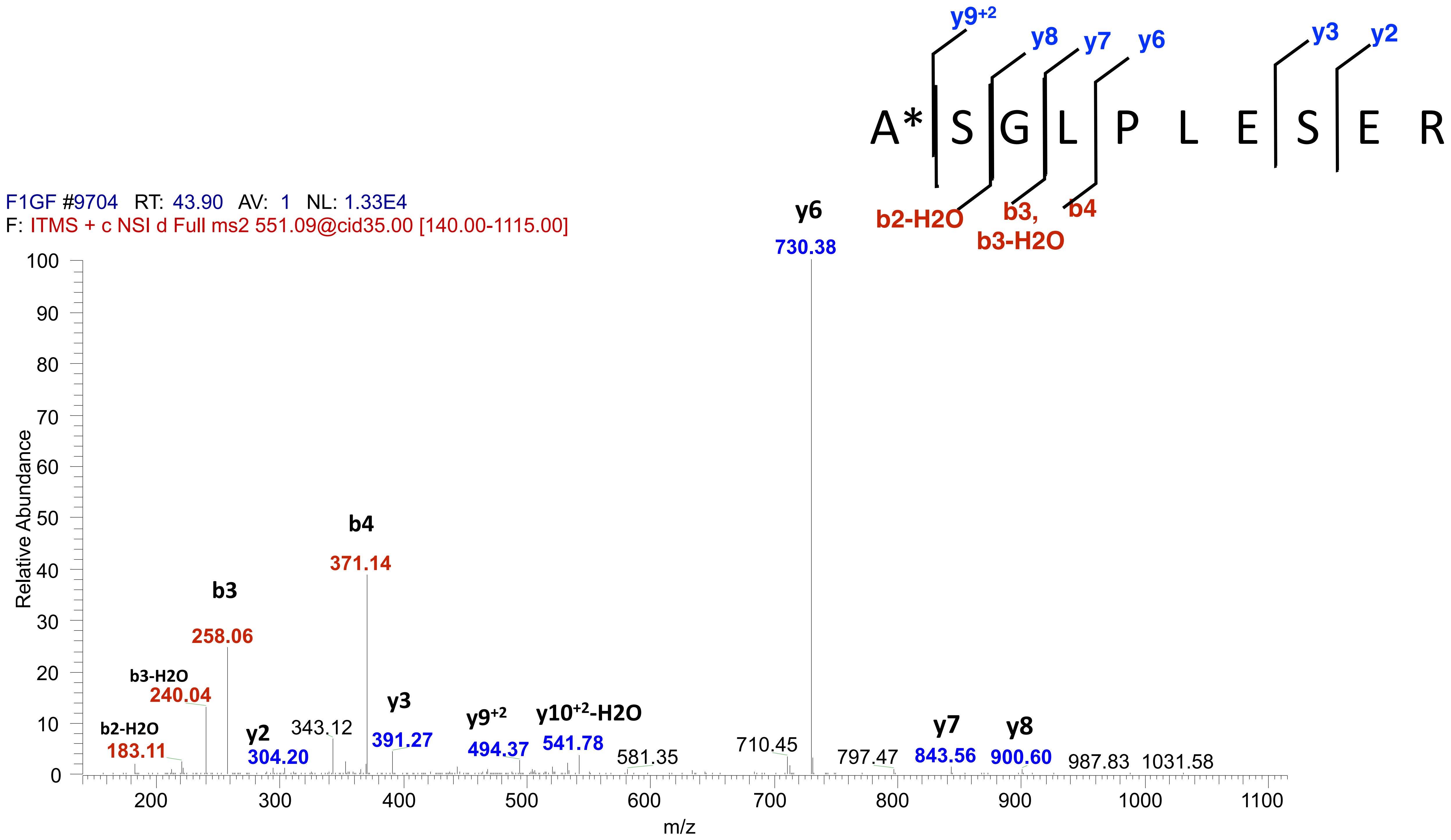

GH (iii) N-term peptide: R*TGDNPTVR; z= +2; XCorr =3.41; Theo. [M+H]1+ = 1057.5279; [M+2H]2+ = 529.37 ; #PSMs =5; * = acetyl

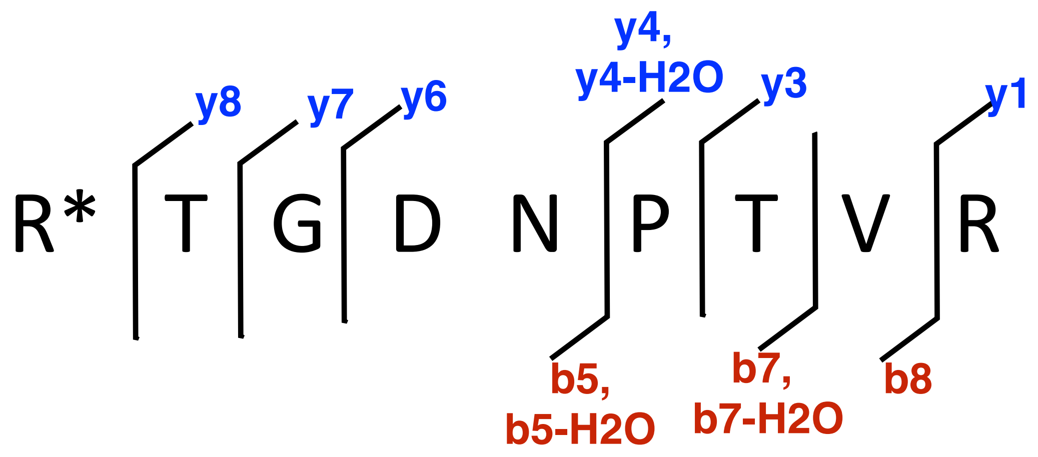

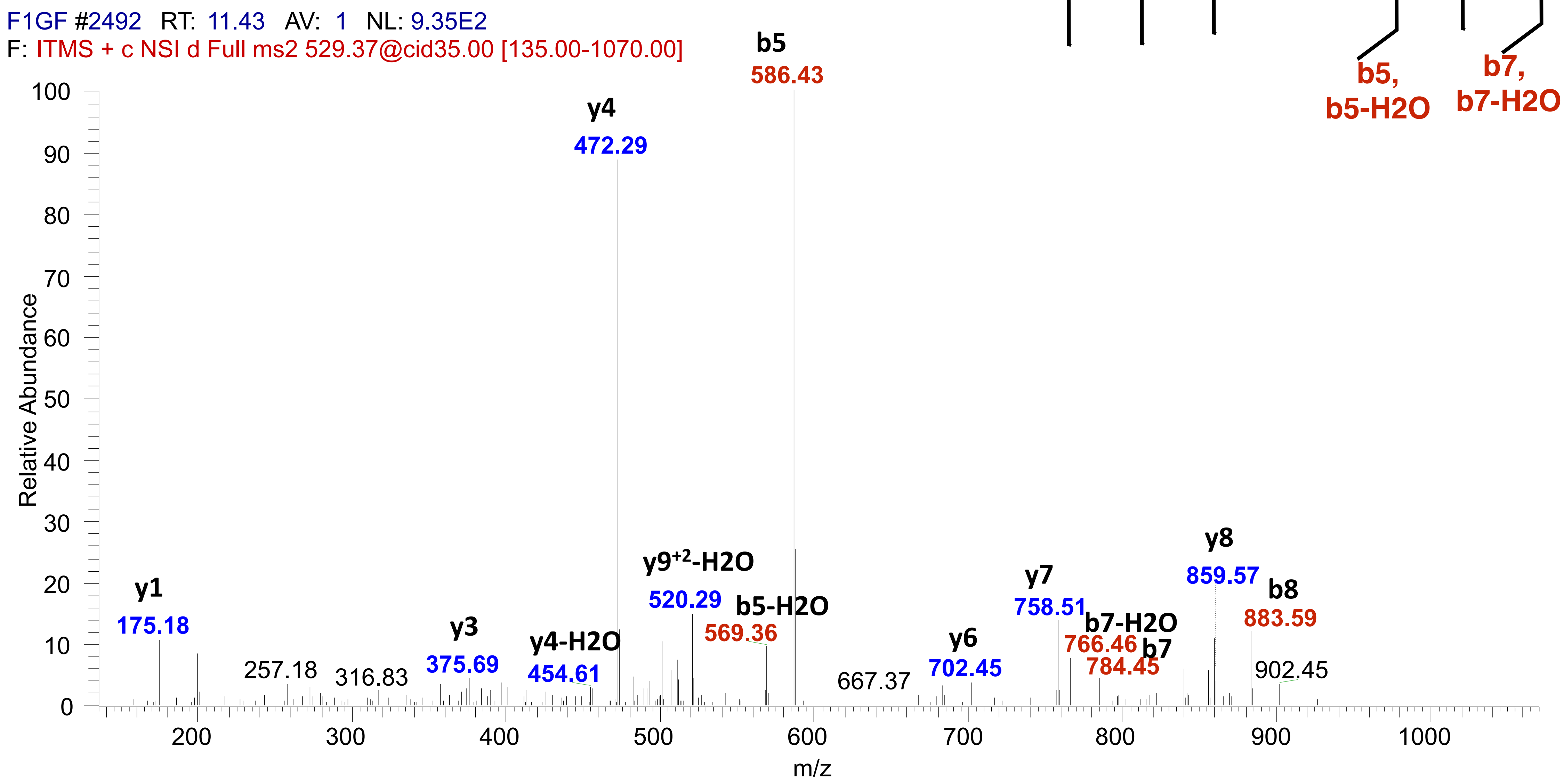

GJ (i) N-term peptide: N*RPEASGLPLESER; z= +2; XCorr =3.22; Theo. [M+H]1+ = 1596.7870; [M+2H]2+ = 799.14; # PSMs = 3; * = acetyl

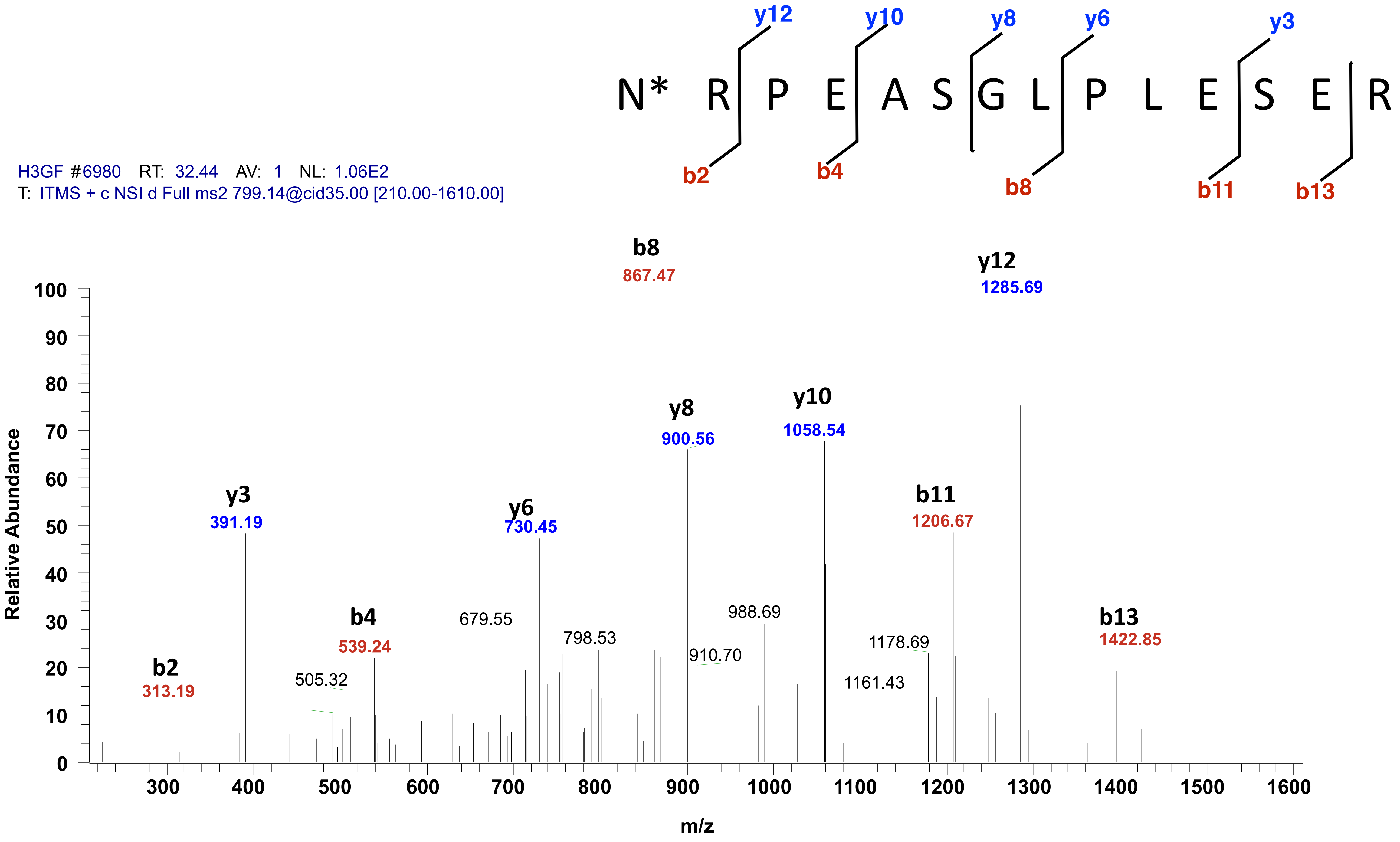

GJ (ii) N-term peptide: A*SGLPLESER; z= +2; XCorr =2.53; Theo. [M+H]1+ = 1100.5476; [M+2H]2+ = 551.91 ; #PSMs = 10; * = acetyl

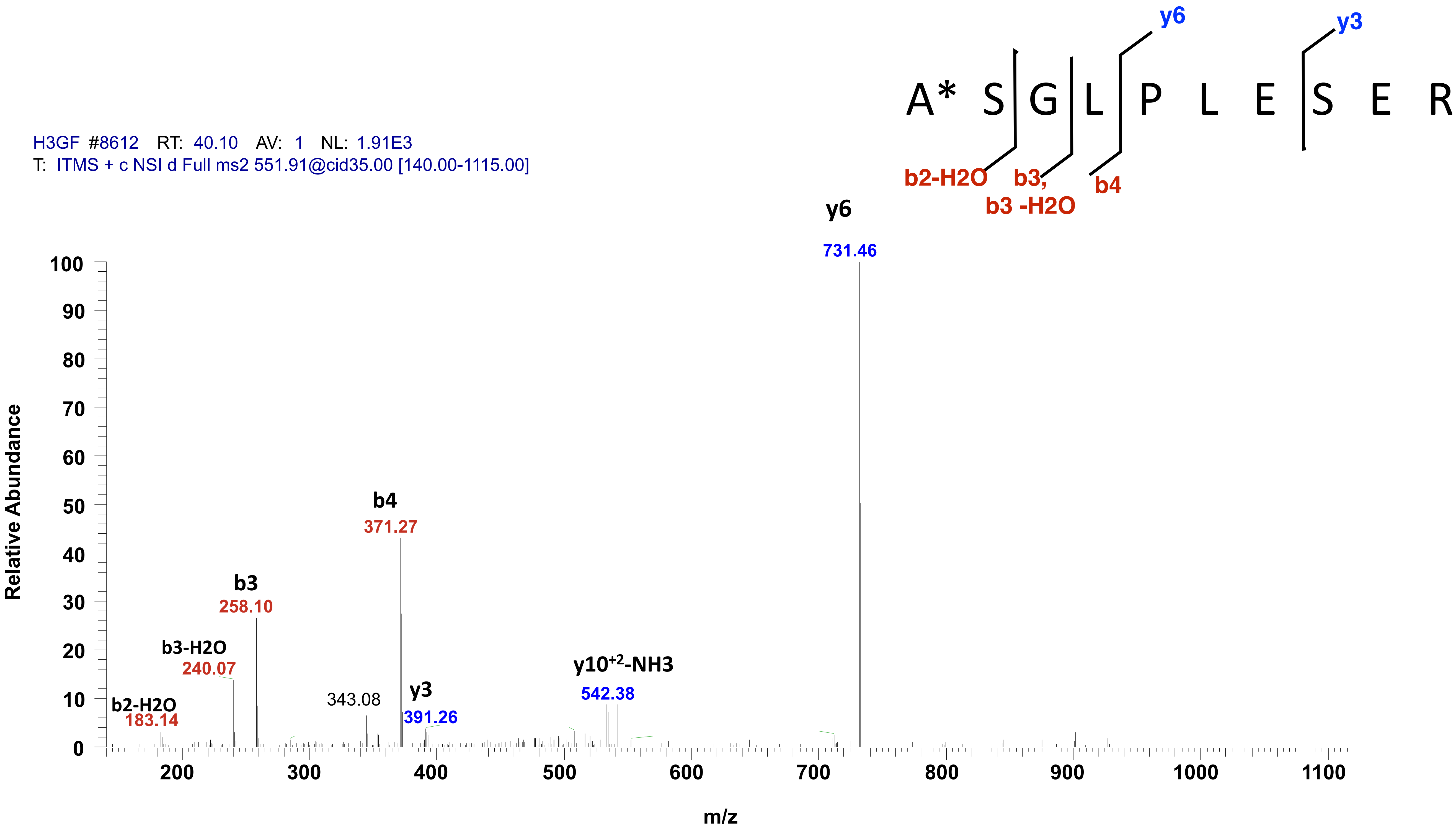

GJ (iii) N-term peptide: R*TGDNPTVR; z= +2; XCorr =2.83; Theo. [M+H]1+ = 1057.5279; [M+2H]2+ = 529.45 ; #PSMs =2; * = acetyl

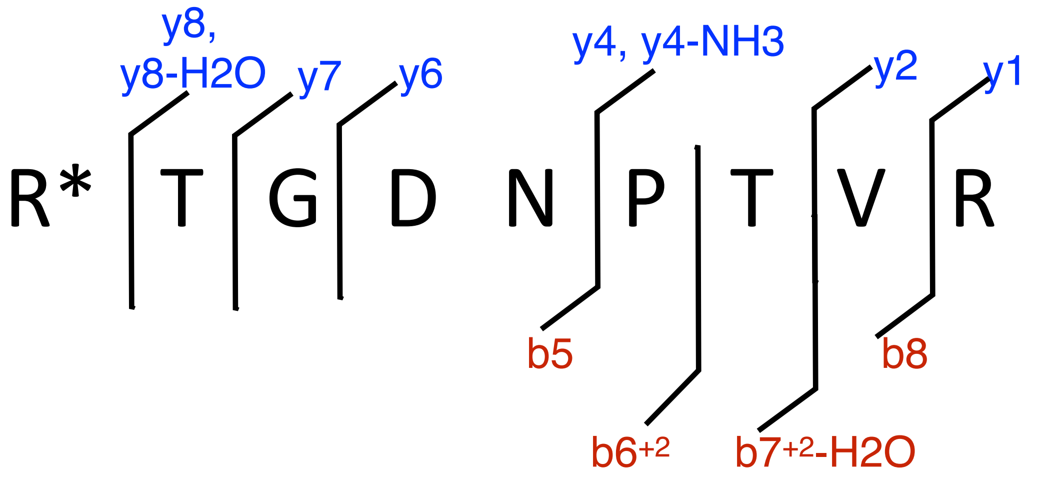

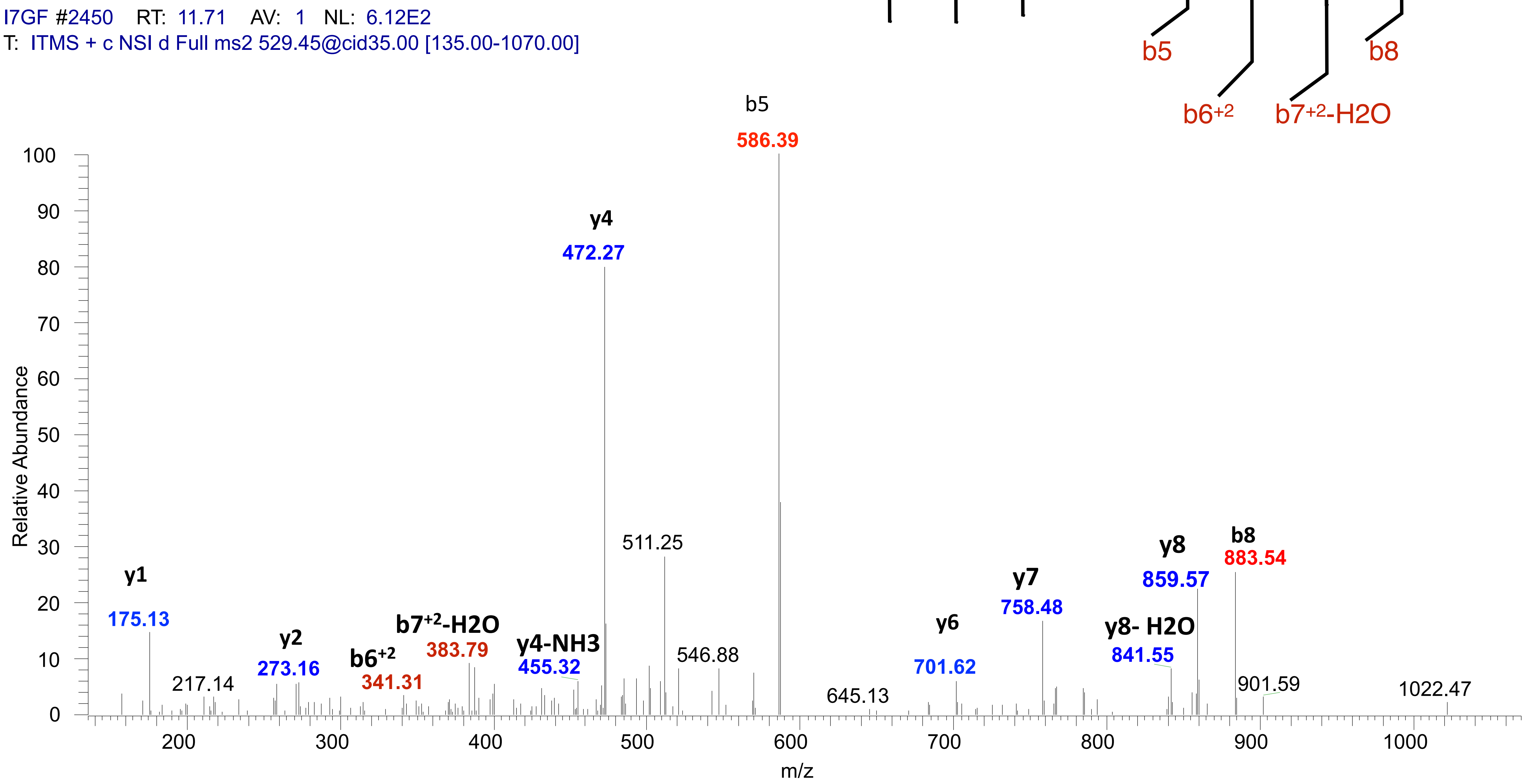

GK N-term peptide: R*TFSTNR; z= +2; XCorr =3.02; Theo. [M+H]1+ = 923.4587; [M+2H]2+ = 462.42 ; #PSMs = 2; * = acetyl

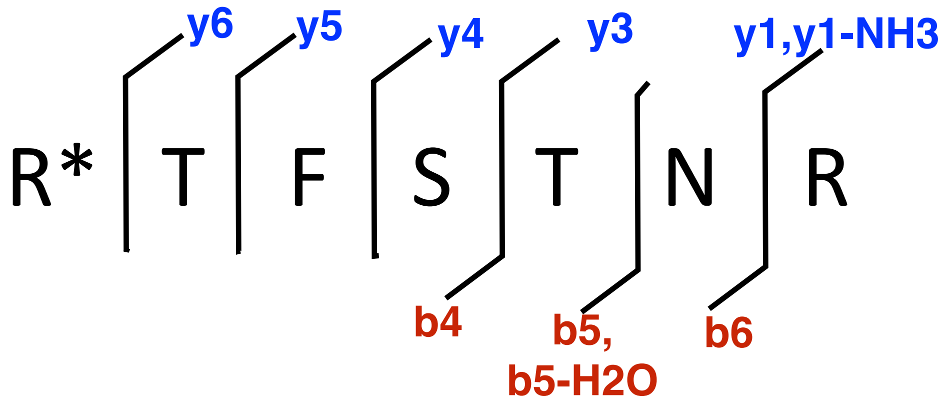

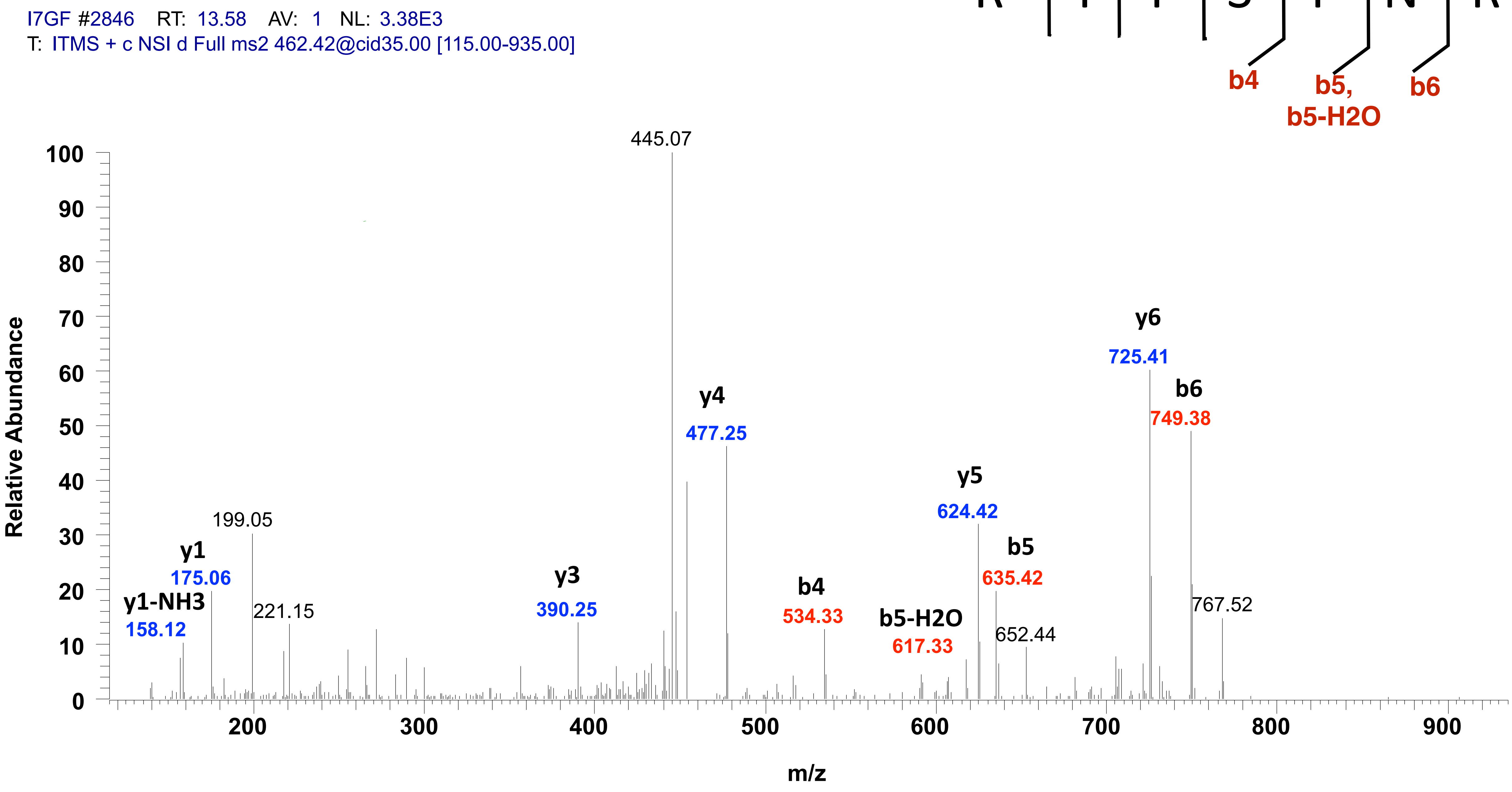

Supplement: Supplementary file 3 — Glu C mapping data (amino terminal identification). (PDF 287 kb) [file 13100_2017_97_MOESM3_ESM.pdf]
