# Supplementary material for: Globular domain structure and function of restriction-like-endonuclease LINEs: similarities to eukaryotic splicing factor Prp8
Source: Mob DNA. 2017 Nov 7;8:16. doi: 10.1186/s13100-017-0097-9 (PMC5678591; doi:10.1186/s13100-017-0097-9)
Supplement: Supplementary file 4 — Glu C mapping data (internal peptides). (PDF 541 kb) [file 13100_2017_97_MOESM4_ESM.pdf]

Supplemental data S3

Globular Domain Structure and Function of Restriction-Like Endonuclease LINEs: Similarities to Eukaryotic Splicing Factor Prp8

M. Murshida Mahbub[1], Saiful M. Chowdhury[2]*, and Shawn M. Christensen[1]*

Figure: Internal peptide mapping of GluC resistant R2 fragments (GA to GJ). Highlighted red texts are N-terminal end peptides for corresponding R2 fragments. Simple green texts represent peptide sequences obtained from GluC-trypsin digestion in a single run of ESI-MS/MS of the corresponding band. Green and italic texts indicate the peptide sequences obtained from GluC-trypsin digestion of the bands from second or following run of ESI-MS/MS. Underlined green texts represent GluC-GluC generated peptides. Blue texts are peptides common between two runs of ESI. Orange texts are peptides common among three runs of ESI.

Number of ESI run of the bands:

Band GA: 5 ESIs. 3 gluc-trp. 2 gluc-gluc. orange is common among 3 runs (2 gluc-trp (blue) &1 gluc-gluc).

Band GC: 5 ESIs. 4 gluc-trp. 1 gluc-gluc. orange is common among 3 runs (3 gluc-trp).

Band GE: 6 ESIs. 4 gluc-trp. 2 gluc-gluc. orange is common among 3 runs (3 gluc-trp)

Band GF: 4 ESIs. 3 gluc-trp.1 gluc-gluc. blue is common among 2 runs (2 gluc-trp)

Band GH: 2 ESIs. 2 gluc-trp. blue is common among 2 runs (2 gluc-trp)

Band GI: 2 ESIs. 1 gluc-trp 1 gluc-gluc. blue is common among 2 runs

Band GJ 2 ESIs. 1 gluc-trp 1 gluc-gluc. blue is common among 2 runs

[illegible]

Band GA

```
MKKSNKENRP EASGLPLESE RTGDNPTVRG
SAGADPVGQD APGWTCQFCE RTFSTNRGLG
VHKRRAHPVE TNTDAAPMMV KRRWHGEEID
LLARTEARLL AERGQCSGGD LFGALPGFGR
TLEAIKGQRR REPYRALVQA HLARFGSQPG
PSSGGCSAEP DFRRASGAEE AGEERCAEDA
AAYDPSAVGQ MSPDAARVLS ELLEGAGRRR
ACRAMRPKTA GRRNDLHDDR TASAHKTSRQ
KRRAEYARVQ ELYKKCRSRA AAEVIDGACG
GVGHSLEEME TYWRPILERV SDAPGPTPEA
LHALGRAEWH GGNRDYTQLW KPISVEEIKA
SRFDWRTSPG PDGIRSGQWR AVPVHLKAEM
FNAWMARGEI PEILRQCRTV FVPKVERPGG
PGEYRPISIA SIPLRHFHSI LARRLLACCP
PDARQRGFIC ADGTLENSAV LDAVLGDSRK
KLRECHVAVL DFAKAFDTVS HEALVELLRL
RGMPEQFCGY IAHLYDTAST TLAVNNEMSS
PVKVGRGVRQ GDPLSPILFN VVMDLILASL
PERVGYRLEM ELVSALAYAD DLVLLAGSKV
GMQESISAVD CVGRQMGLRL NCRKSAVLSM
IPDGHRKKHH YLTERTFNIG GKPLRQVSCV
ERWRYLGVDF EASGCVTLEH SISSALNNIS
RAPLKPQQRL EILRAHLIPR FQHGFVLGNI
SDDRLRMLDV QIRKAVGQWL RLPADVPKAY
YHAAVQDGGL AIPSVRATIP DLIVRRFGGL
DSSPWSVARA AAKSDKIRKK LRWAWKQLRR
FSRVDSTTQR PSVRLFWREH LHASVDGREL
RESTRTPTST KWIRERCAQI TGRDFVQFVH
THINALPSRI RGSRGRRGGG ESSLTCRAGC
KVRETTAHIL QQCHRTHGGR ILRHNKIVSF
VAKAMEENKW TVELEPRLRT SVGLRKPDII
ASRDGVGVIV DVQVVSGQRS LDELHREKRN
KYGNHGELVE LVAGRLGLPK AECVRATSCT
ISWRGVWSLT SYKELRSIIG LREPTLQIVP
ILALRGSHMN WTRFNQMTSV MGGGVGIEGR
HHHHHH
```

Band GB

```
MKKSNKENRP EASGLPLESE RTGDNPTVRG
SAGADPVGQD APGWTCQFCE RTFSTNRGLG
VHKRRAHPVE TNTDAAPMMV KRRWHGEEID
LLARTEARLL AERGQCSGGD LFGALPGFGR
TLEAIKGQRR REPYRALVQA HLARFGSQPG
PSSGGCSAEP DFRRASGAEE AGEERCAEDA
AAYDPSAVGQ MSPDAARVLS ELLEGAGRRR
ACRAMRPKTA GRRNDLHDDR TASAHKTSRQ
KRRAEYARVQ ELYKKCRSRA AAEVIDGACG
GVGHSLEEME TYWRPILERV SDAPGPTPEA
LHALGRAEWH GGNRDYTQLW KPISVEEIKA
SRFDWRTSPG PDGIRSGQWR AVPVHLKAEM
FNAWMARGEI PEILRQCRTV FVPKVERPGG
PGEYRPISIA SIPLRHFHSI LARRLLACCP
PDARQRGFIC ADGTLENSAV LDAVLGDSRK
KLRECHVAVL DFAKAFDTVS HEALVELLRL
RGMPEQFCGY IAHLYDTAST TLAVNNEMSS
PVKVGRGVRQ GDPLSPILFN VVMDLILASL
PERVGYRLEM ELVSALAYAD DLVLLAGSKV
GMQESISAVD CVGRQMGLRL NCRKSAVLSM
IPDGHRKKHH YLTERTFNIG GKPLRQVSCV
ERWRYLGVDF EASGCVTLEH SISSALNNIS
RAPLKPQQRL EILRAHLIPR FQHGFVLGNI
SDDRLRMLDV QIRKAVGQWL RLPADVPKAY
YHAAVQDGGL AIPSVRATIP DLIVRRFGGL
DSSPWSVARA AAKSDKIRKK LRWAWKQLRR
FSRVDSTTQR PSVRLFWREH LHASVDGREL
RESTRTPTST KWIRERCAQI TGRDFVQFVH
THINALPSRI RGSRGRRGGG ESSLTCRAGC
KVRETTAHIL QQCHRTHGGR ILRHNKIVSF
VAKAMEENKW TVELEPRLRT SVGLRKPDII
ASRDGVGVIV DVQVVSGQRS LDELHREKRN
KYGNHGELVE LVAGRLGLPK AECVRATSCT
ISWRGVWSLT SYKELRSIIG LREPTLQIVP
ILALRGSHMN WTRFNQMTSV MGGGVGIEGR
HHHHHH
```

Band GC

```
MKKSNKENRP EASGLPLESE RTGDNPTVRG
SAGADPVGQD APGWTCQFCE RTFSTNRGLG
VHKRRAHPVE TNTDAAPMMV KRRWHGEEID
LLARTEARLL AERGQCSGGD LFGALPGFGR
TLEAIKGQRR REPYRALVQA HLARFGSQPG
PSSGGCSAEP DFRRASGAEE AGEERCAEDA
AAYDPSAVGQ MSPDAARVLS ELLEGAGRRR
ACRAMRPKTA GRRNDLHDDR TASAHKTSRQ
KRRAEYARVQ ELYKKCRSRA AAEVIDGACG
GVGHSLEEME TYWRPILERV SDAPGPTPEA
LHALGRAEWH GGNRDYTQLW KPISVEEIKA
SRFDWRTSPG PDGIRSGQWR AVPVHLKAEM
FNAWMARGEI PEILRQCRTV FVPKVERPGG
PGEYRPISIA SIPLRHFHSI LARRLLACCP
PDARQRGFIC ADGTLENSAV LDAVLGDSRK
KLRECHVAVL DFAKAFDTVS HEALVELLRL
RGMPEQFCGY IAHLYDTAST TLAVNNEMSS
PVKVGRGVRQ GDPLSPILFN VVMDLILASL
PERVGYRLEM ELVSALAYAD DLVLLAGSKV
GMQESISAVD CVGRQMGLRL NCRKSAVLSM
IPDGHRKKHH YLTERTFNIG GKPLRQVSCV
ERWRYLGVDF EASGCVTLEH SISSALNNIS
RAPLKPQQRL EILRAHLIPR FQHGFVLGNI
SDDRLRMLDV QIRKAVGQWL RLPADVPKAY
YHAAVQDGGL AIPSVRATIP DLIVRRFGGL
DSSPWSVARA AAKSDKIRKK LRWAWKQLRR
FSRVDSTTQR PSVRLFWREH LHASVDGREL
RESTRTPTST KWIRERCAQI TGRDFVQFVH
THINALPSRI RGSRGRRGGG ESSLTCRAGC
KVRETTAHIL QQCHRTHGGR ILRHNKIVSF
VAKAMEENKW TVELEPRLRT SVGLRKPDII
ASRDGVGVIV DVQVVSGQRS LDELHREKRN
KYGNHGELVE LVAGRLGLPK AECVRATSCT
ISWRGVWSLT SYKELRSIIG LREPTLQIVP
ILALRGSHMN WTRFNQMTSV MGGGVGIEGR
HHHHHH
```

Band GD

MKKSNKENRP EASGLPLESE RTGDNPTVRG
SAGADPVGQD APGWTCQFCE RTFSTNRGLG
VHKRRAHPVE TNTDAAPMMV KRRWHGEEID
LLARTEARLL AERGQCSGGD LFGALPGFGR
TLEAIKGQRR REPYRALVQA HLARFGSQPG
PSSGGCSAEP DFRRASGAEE AGEERCAEDA
AAYDPSAVGQ MSPDAARVLS ELLEGAGRRR
ACRAMRPKTA GRRNDLHDDR TASAHKTSRQ
KRRAEYARVQ ELYKKCRSRA AAEVIDGACG
GVGHSLEEME TYWRPILERV SDAPGPTPEA
LHALGRAEWH GGNRDYTQLW KPISVEEIKA
SRFDWRTSPG PDGIRSGQWR AVPVHLKAEM
FNAWMARGEI PEILRQCRTV FVPKVERPGG
PGEYRPISIA SIPLRHFHSI LARRLLACCP
PDARQRGFIC ADGTLENSAV LDAVLGDSRK
KLRECHVAVL DFAKAFDTVS HEALVELLRL
RGMPEQFCGY IAHLYDTAST TLAVNNEMSS
PVKVGRGVRQ GDPLSPILFN VVMDLILASL
PERVGYRLEM ELVSALAYAD DLVLLAGSKV
GMQESISAVD CVGRQMGLRL NCRKSAVLSM
IPDGHRKKHH YLTERTFNIG GKPLRQVSCV
ERWRYLGVDF EASGCVTLEH SISSALNNIS
RAPLKPQQRL EILRAHLIPR FQHGFVLGNI
SDDRLRMLDV QIRKAVGQWL RLPADVPKAY
YHAAVQDGGL AIPSVRATIP DLIVRRFGGL
DSSPWSVARA AAKSDKIRKK LRWAWKQLRR
FSRVDSTTQR PSVRLFWREH LHASVDGREL
RESTRTPTST KWIRERCAQI TGRDFVQFVH
THINALPSRI RGSRGRRGGG ESSLTCRAGC
KVRETTAHIL QQCHRTHGGR ILRHNKIVSF
VAKAMEENKW TVELEPRLRT SVGLRKPDII
ASRDGVGVIV DVQVVSGQRS LDELHREKRN
KYGNHGELVE LVAGRLGLPK AECVRATSCT
ISWRGVWSLT SYKELRSIIG LREPTLQIVP
ILALRGSHMN WTRFNQMTSV MGGGVGIEGR
HHHHHH

Band GE

MKKSNKENRP EASGLPLESE RTGDNPTVRG
SAGADPVGQD APGWTCQFCE RTFSTNRGLG
VHKRRAHPVE TNTDAAPMMV KRRWHGEEID
LLARTEARLL AERGQCSGGD LFGALPGFGR
TLEAIKGQRR REPYRALVQA HLARFGSQPG
PSSGGCSAEP DFRRASGAEE AGEERCAEDA
AAYDPSAVGQ MSPDAARVLS ELLEGAGRRR
ACRAMRPKTA GRRNDLHDDR TASAHKTSRQ
KRRAEYARVQ ELYKKCRSRA AAEVIDGACG
GVGHSLEEME TYWRPILERV SDAPGPTPEA
LHALGRAEWH GGNRDYTQLW KPISVEEIKA
SRFDWRTSPG PDGIRSGQWR AVPVHLKAEM
FNAWMARGEI PEILRQCRTV FVPKVERPGG
PGEYRPISIA SIPLRHFHSI LARRLLACCP
PDARQRGFIC ADGTLENSAV LDAVLGDSRK
KLRECHVAVL DFAKAFDTVS HEALVELLRL
RGMPEQFCGY IAHLYDTAST TLAVNNEMSS
PVKVGRGVRQ GDPLSPILFN VVMDLILASL
PERVGYRLEM ELVSALAYAD DLVLLAGSKV
GMQESISAVD CVGRQMGLRL NCRKSAVLSM
IPDGHRKKHH YLTERTFNIG GKPLRQVSCV
ERWRYLGVDF EASGCVTLEH SISSALNNIS
RAPLKPQQRL EILRAHLIPR FQHGFVLGNI
SDDRLRMLDV QIRKAVGQWL RLPADVPKAY
YHAAVQDGGL AIPSVRATIP DLIVRRFGGL
DSSPWSVARA AAKSDKIRKK LRWAWKQLRR
FSRVDSTTQR PSVRLFWREH LHASVDGREL
RESTRTPTST KWIRERCAQI TGRDFVQFVH
THINALPSRI RGSRGRRGGG ESSLTCRAGC
KVRETTAHIL QQCHRTHGGR ILRHNKIVSF
VAKAMEENKW TVELEPRLRT SVGLRKPDII
ASRDGVGVIV DVQVVSGQRS LDELHREKRN
KYGNHGELVE LVAGRLGLPK AECVRATSCT
ISWRGVWSLT SYKELRSIIG LREPTLQIVP
ILALRGSHMN WTRFNQMTSV MGGGVGIEGR
HHHHHH

Band GF

MKKSNKENRP EASGLPLESE RTGDNPTVRG
SAGADPVGQD APGWTCQFCE RTFSTNRGLG
VHKRRAHPVE TNTDAAPMMV KRRWHGEEID
LLARTEARLL AERGQCSGGD LFGALPGFGR
TLEAIKGQRR REPYRALVQA HLARFGSQPG
PSSGGCSAEP DFRRASGAEE AGEERCAEDA
AAYDPSAVGQ MSPDAARVLS ELLEGAGRRR
ACRAMRPKTA GRRNDLHDDR TASAHKTSRQ
KRRAEYARVQ ELYKKCRSRA AAEVIDGACG
GVGHSLEEME TYWRPILERV SDAPGPTPEA
LHALGRAEWH GGNRDYTQLW KPISVEEIKA
SRFDWRTSPG PDGIRSGQWR AVPVHLKAEM
FNAWMARGEI PEILRQCRTV FVPKVERPGG
PGEYRPISIA SIPLRHFHSI LARRLLACCP
PDARQRGFIC ADGTLENSAV LDAVLGDSRK
KLRECHVAVL DFAKAFDTVS HEALVELLRL
RGMPEQFCGY IAHLYDTAST TLAVNNEMSS
PVKVGRGVRQ GDPLSPILFN VVMDLILASL
PERVGYRLEM ELVSALAYAD DLVLLAGSKV
GMQESISAVD CVGRQMGLRL NCRKSAVLSM
IPDGHRKKHH YLTERTFNIG GKPLRQVSCV
ERWRYLGVDF EASGCVTLEH SISSALNNIS
RAPLKPQQRL EILRAHLIPR FQHGFVLGNI
SDDRLRMLDV QIRKAVGQWL RLPADVPKAY
YHAAVQDGGL AIPSVRATIP DLIVRRFGGL
DSSPWSVARA AAKSDKIRKK LRWAWKQLRR
FSRVDSTTQR PSVRLFWREH LHASVDGREL
RESTRTPTST KWIRERCAQI TGRDFVQFVH
THINALPSRI RGSRGRRGGG ESSLTCRAGC
KVRETTAHIL QQCHRTHGGR ILRHNKIVSF
VAKAMEENKW TVELEPRLRT SVGLRKPDII
ASRDGVGVIV DVQVVSGQRS LDELHREKRN
KYGNHGELVE LVAGRLGLPK AECVRATSCT
ISWRGVWSLT SYKELRSIIG LREPTLQIVP
ILALRGSHMN WTRFNQMTSV MGGGVGIEGR
HHHHHH

Band GH

MKKSNKENRP EASGLPLESE RTGDNPTVRG
SAGADPVGQD APGWTCQFCE RTFSTNRGLG
VHKRRAHPVE TNTDAAPMMV KRRWHGEEID
LLARTEARLL AERGQCSGGD LFGALPGFGR
TLEAIKGQRR REPYRALVQA HLARFGSQPG
PSSGGCSAEP DFRRASGAEE AGEERCAEDA
AAYDPSAVGQ MSPDAARVLS ELLEGAGRRR
ACRAMRPKTA GRRNDLHDDR TASAHKTSRQ
KRRAEYARVQ ELYKKCRSRA AAEVIDGACG
GVGHSLEEME TYWRPILERV SDAPGPTPEA
LHALGRAEWH GGNRDYTQLW KPISVEEIKA
SRFDWRTSPG PDGIRSGQWR AVPVHLKAEM
FNAWMARGEI PEILRQCRTV FVPKVERPGG
PGEYRPISIA SIPLRHFHSI LARRLLACCP
PDARQRGFIC ADGTLENSAV LDAVLGDSRK
KLRECHVAVL DFAKAFDTVS HEALVELLRL
RGMPEQFCGY IAHLYDTAST TLAVNNEMSS
PVKVGRGVRQ GDPLSPILFN VVMDLILASL
PERVGYRLEM ELVSALAYAD DLVLLAGSKV
GMQESISAVD CVGRQMGLRL NCRKSAVLSM
IPDGHRKKHH YLTERTFNIG GKPLRQVSCV
ERWRYLGVDF EASGCVTLEH SISSALNNIS
RAPLKPQQRL EILRAHLIPR FQHGFVLGNI
SDDRLRMLDV QIRKAVGQWL RLPADVPKAY
YHAAVQDGGL AIPSVRATIP DLIVRRFGGL
DSSPWSVARA AAKSDKIRKK LRWAWKQLRR
FSRVDSTTQR PSVRLFWREH LHASVDGREL
RESTRTPTST KWIRERCAQI TGRDFVQFVH
THINALPSRI RGSRGRRGGG ESSLTCRAGC
KVRETTAHIL QQCHRTHGGR ILRHNKIVSF
VAKAMEENKW TVELEPRLRT SVGLRKPDII
ASRDGVGVIV DVQVVSGQRS LDELHREKRN
KYGNHGELVE LVAGRLGLPK AECVRATSCT
ISWRGVWSLT SYKELRSIIG LREPTLQIVP
ILALRGSHMN WTRFNQMTSV MGGGVGIEGR
HHHHHH

Band GJ

MKKSNKENRP EASGLPLESE RTGDNPTVRG
SAGADPVGQD APGWTCQFCE RTFSTNRGLG
VHKRRAHPVE TNTDAAPMMV KRRWHGEEID
LLARTEARLL AERGQCSGGD LFGALPGFGR
TLEAIKGQRR REPYRALVQA HLARFGSQPG
PSSGGCSAEP DFRRASGAEE AGEERCAEDA
AAYDPSAVGQ MSPDAARVLS ELLEGAGRRR
ACRAMRPKTA GRRNDLHDDR TASAHKTSRQ
KRRAEYARVQ ELYKKCRSRA AAEVIDGACG
GVGHSLEEME TYWRPILERV SDAPGPTPEA
LHALGRAEWH GGNRDYTQLW KPISVEEIKA
SRFDWRTSPG PDGIRSGQWR AVPVHLKAEM
FNAWMARGEI PEILRQCRTV FVPKVERPGG
PGEYRPISIA SIPLRHFHSI LARRLLACCP
PDARQRGFIC ADGTLENSAV LDAVLGDSRK
KLRECHVAVL DFAKAFDTVS HEALVELLRL
RGMPEQFCGY IAHLYDTAST TLAVNNEMSS
PVKVGRGVRQ GDPLSPILFN VVMDLILASL
PERVGYRLEM ELVSALAYAD DLVLLAGSKV
GMQESISAVD CVGRQMGLRL NCRKSAVLSM
IPDGHRKKHH YLTERTFNIG GKPLRQVSCV
ERWRYLGVDF EASGCVTLEH SISSALNNIS
RAPLKPQQRL EILRAHLIPR FQHGFVLGNI
SDDRLRMLDV QIRKAVGQWL RLPADVPKAY
YHAAVQDGGL AIPSVRATIP DLIVRRFGGL
DSSPWSVARA AAKSDKIRKK LRWAWKQLRR
FSRVDSTTQR PSVRLFWREH LHASVDGREL
RESTRTPTST KWIRERCAQI TGRDFVQFVH
THINALPSRI RGSRGRRGGG ESSLTCRAGC
KVRETTAHIL QQCHRTHGGR ILRHNKIVSF
VAKAMEENKW TVELEPRLRT SVGLRKPDII
ASRDGVGVIV DVQVVSGQRS LDELHREKRN
KYGNHGELVE LVAGRLGLPK AECVRATSCT
ISWRGVWSLT SYKELRSIIG LREPTLQIVP
ILALRGSHMN WTRFNQMTSV MGGGVGIEGR
HHHHHH

Band GK

MKKSNKENRP EASGLPLESE RTGDNPTVRG
SAGADPVGQD APGWTCQFCE RTFSTNRGLG
VHKRRAHPVE TNTDAAPMMV KRRWHGEEID
LLARTEARLL AERGQCSGGD LFGALPGFGR
TLEAIKGQRR REPYRALVQA HLARFGSQPG
PSSGGCSAEP DFRRASGAEE AGEERCAEDA
AAYDPSAVGQ MSPDAARVLS ELLEGAGRRR
ACRAMRPKTA GRRNDLHDDR TASAHKTSRQ
KRRAEYARVQ ELYKKCRSRA AAEVIDGACG
GVGHSLEEME TYWRPILERV SDAPGPTPEA
LHALGRAEWH GGNRDYTQLW KPISVEEIKA
SRFDWRTSPG PDGIRSGQWR AVPVHLKAEM
FNAWMARGEI PEILRQCRTV FVPKVERPGG
PGEYRPISIA SIPLRHFHSI LARRLLACCP
PDARQRGFIC ADGTLENSAV LDAVLGDSRK
KLRECHVAVL DFAKAFDTVS HEALVELLRL
RGMPEQFCGY IAHLYDTAST TLAVNNEMSS
PVKVGRGVRQ GDPLSPILFN VVMDLILASL
PERVGYRLEM ELVSALAYAD DLVLLAGSKV
GMQESISAVD CVGRQMGLRL NCRKSAVLSM
IPDGHRKKHH YLTERTFNIG GKPLRQVSCV
ERWRYLGVDF EASGCVTLEH SISSALNNIS
RAPLKPQQRL EILRAHLIPR FQHGFVLGNI
SDDRLRMLDV QIRKAVGQWL RLPADVPKAY
YHAAVQDGGL AIPSVRATIP DLIVRRFGGL
DSSPWSVARA AAKSDKIRKK LRWAWKQLRR
FSRVDSTTQR PSVRLFWREH LHASVDGREL
RESTRTPTST KWIRERCAQI TGRDFVQFVH
THINALPSRI RGSRGRRGGG ESSLTCRAGC
KVRETTAHIL QQCHRTHGGR ILRHNKIVSF
VAKAMEENKW TVELEPRLRT SVGLRKPDII
ASRDGVGVIV DVQVVSGQRS LDELHREKRN
KYGNHGELVE LVAGRLGLPK AECVRATSCT
ISWRGVWSLT SYKELRSIIG LREPTLQIVP
ILALRGSHMN WTRFNQMTSV MGGGVGIEGR
HHHHHH
