# Supplementary material for: Globular domain structure and function of restriction-like-endonuclease LINEs: similarities to eukaryotic splicing factor Prp8
Source: Mob DNA. 2017 Nov 7;8:16. doi: 10.1186/s13100-017-0097-9 (PMC5678591; doi:10.1186/s13100-017-0097-9)

Supplemental data S5 A

Globular Domain Structure and Function of Restriction-Like-Endonuclease LINEs: Similarities to Eukaryotic Splicing Factor Prp8

M. Murshida Mahbub[1], Saiful M. Chowdhury[2*], and Shawn M. Christensen[1*]

Figure: Multiple sequence alignments (MSA) of protein sequences encoded by R2 elements, RLE LINEs, APE LINEs, RVT genes, group II introns, nMat and Prp8. MSAs were generated in PROMALS3D server. Secondary structure information was plotted on MSA using Ali2D tool of the MPI bioinformatic toolkits. The alignments begin at alpha helix of RT0 and do not include alpha helix of the index finger and that traverses the palm. The alpha helices are colored in red and beta strands are colored in blue. Tentative positions of reverse transcriptase subdomains are indicated. Sequences were obtained from NCBI, Uniprot or Repbase database.

Confidence:
0123456789
HHHHHHHHHH
EEEEEEEEEE

R2

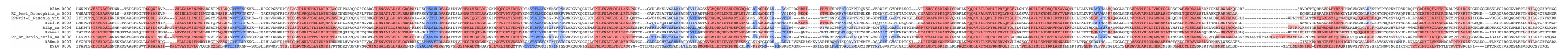

RLE LINEs

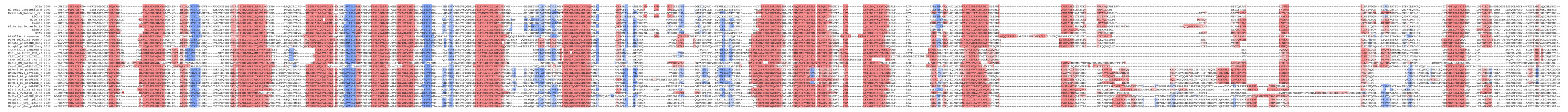

APE LINEs

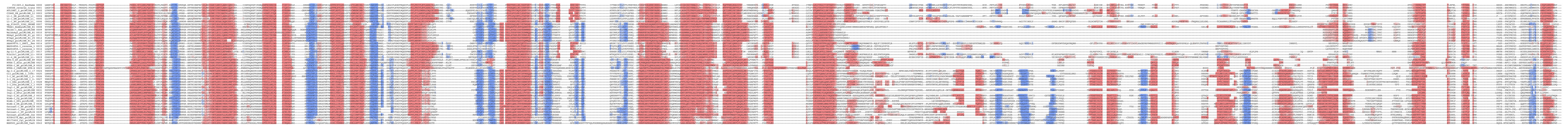

RVT

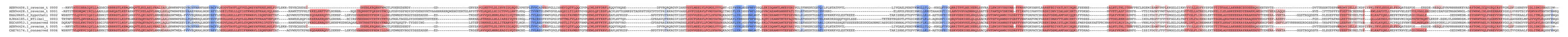

group II introns and nMat proteins

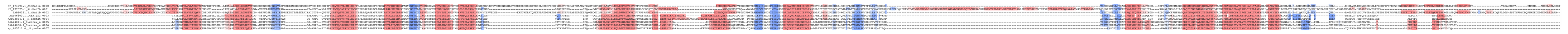

Prp8

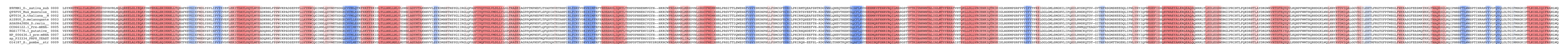

100 sequences_RT through CCHC

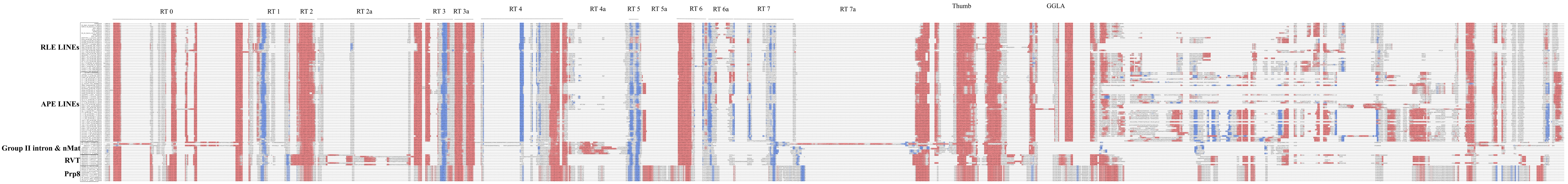

100 sequences_GGLG through CCHC

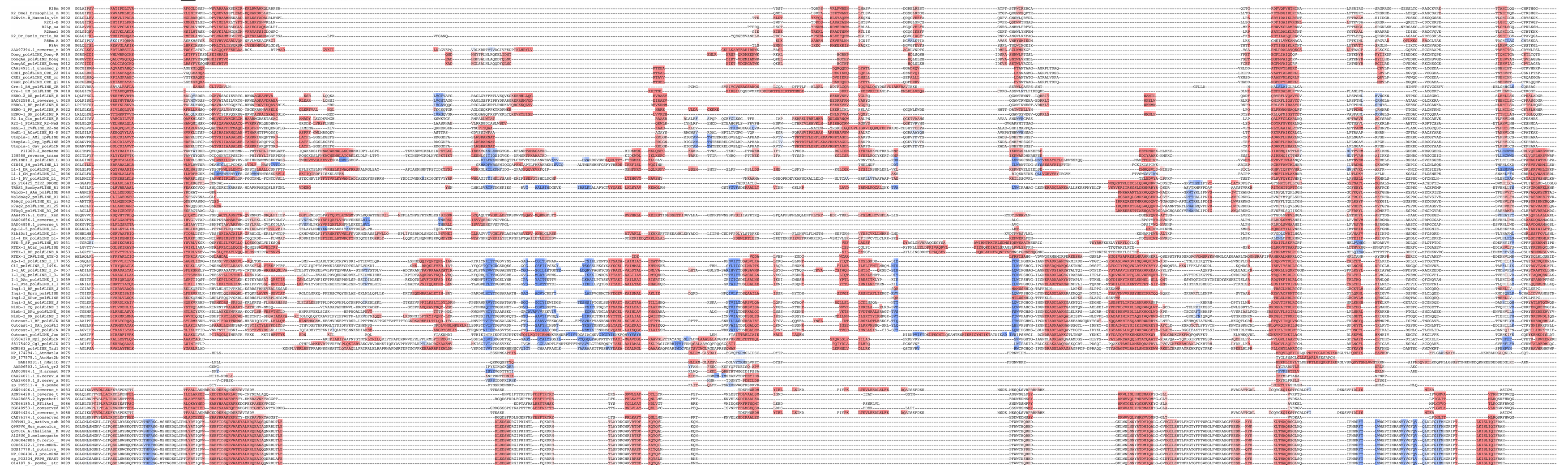

Supplement: Supplementary file 7 — Alignment files. (ZIP 5764 kb) [file supplemental_S5A.pdf › supplemental_S5A.pdf]
